# Untargeted Metabolomic Profiling and Antioxidant Capacities of Different Solvent Crude Extracts of *Ephedra foeminea*

**DOI:** 10.3390/metabo12050451

**Published:** 2022-05-17

**Authors:** Ruba Al-Nemi, Arwa A. Makki, Khaled Sawalha, Dina Hajjar, Mariusz Jaremko

**Affiliations:** 1 Department of Biochemistry, College of Science, University of Jeddah, P.O. Box 80327, Jeddah 21589, Saudi Arabia; rubamunemi@gmail.com (R.A.-N.); amaki@uj.edu.sa (A.A.M.); 2 Department of Biological Sciences, Al Quds University, P.O. Box 51000, Jerusalem 9103401, Palestine; ksawalha@staff.alquds.edu; 3 Smart-Health Initiative and Red Sea Research Center, Division of Biological and Environmental Sciences and Engineering, King Abdullah University of Science and Technology, P.O. Box 4700, Thuwal 23955-6900, Saudi Arabia

**Keywords:** *Ephedra foeminea*, Ephedraceae, NMR, GC–MS, LC–MS, untargeted metabolomics, antioxidant activity, crude extracts

## Abstract

*Ephedra foeminea* is a traditional medicinal plant used in the Eastern Mediterranean region. This study aims to investigate the chemical profiles of different solvent extracts of *E. foeminea* via an untargeted metabolomics approach, alongside determining their antioxidant capacities. *E. foeminea* samples collected from Jordan were macerated in solvents of varying polarities; dichloromethane/methanol, methanol, ethanol, ethyl acetate, and acetone. The crude extracts were subjected to comprehensive chemical profiling and metabolomics study using Gas chromatography–Mass spectrometry (GC–MS), Liquid chromatography–Mass spectrometry (LC–MS), and Nuclear Magnetic Resonance (NMR). The obtained data were analyzed using Venn diagrams, Principle Component Analysis (PCA), and Metabolite Enrichment Set Analysis (MESA). ABTS assay was performed to measure the crude extracts’ antioxidant activity. MESA revealed the dominant chemical groups as amino acids, fatty acids, carboxylic acids, and carbohydrates. Results indicated that dichloromethane/methanol and methanolic extracts had the most distinct composition as well as the most unique compounds. The methanolic extract had the most potency (IC_50_ 249.6 µg/mL) in the ABTS assay. However, no significant differences were found. In conclusion, solvents influenced the recovery of metabolites in *E. foeminea* and the antioxidant activity of the *E. foeminea* methanolic extract could be correlated to the abundant presence of diverse bioactive compounds.

## 1. Introduction

The East Mediterranean region harbors an abundance of traditionally medicinal plants often used in folk medicine to treat various diseases and conditions [1,2,3,4]. *Ephedra foeminea*, also known as “Alandah” or “Qudab” in Arabic, is a common and widespread plant in the East Mediterranean habitats which is similar to the popular Chinese species *Ephedra sinica*, or “Ma Huang” as it is known in Traditional Chinese Medicine. *E. foeminea* a member of the family Ephedraceae, is a shrub and a perennial climber with multi branches, stems, and fruits. The leaves are small in size and whorled at nodes of cylindrical stems. A published study indicated that *E. foeminea* is the only Ephedra species which does not contain ephedrine and pseudoephedrine [5], which may have adverse effects on the central nervous system, blood pressure, and pulse, making it a relatively safe therapeutic candidate. Ephedra’s use in folk medicine is as a remedy for a wide range of illnesses including allergies, bronchial asthma, chills, colds, coughs, edema, fever, flu, headaches, and nasal congestion [6]. A recent study of the traditional usage of *E. foeminea* in Arabic, Hebrew, and Judeo-Arabic medical literature reported the plant’s application in traditional Arab medicine as a treatment for many ailments [7]. The components of plants can vary between species and origins and may lead to diverse metabolic phenotypes [8,9]. The extraction solvent and drying process used can also have a significant impact on the biological activities and metabolite contents of plant materials because distinct bioactive constituents with different chemical properties and polarities may have varying solubility in different solvents [10,11].

Metabolomics has evolved in recent years as a vital method for comprehensively evaluating all metabolites in a crude extract while offering a reasonable metabolic snapshot of a plant, aiding the development of natural product-derived therapeutic agents [12]. Several Ephedra species have previously been shown to be rich in antioxidant phenolic compounds such as transcinnamic acid, catechin, syringin, epicatechin, symplocoside, kaempferol 3-O-rhamnoside-7-O-glucoside, and isovitexin-O-rhamnoside, which contributed significantly to the antioxidant potential of the plant [5,13,14,15]. These constituents could prevent many diseases when used in food, cosmetics, and pharmaceutical products. A recent study by Soumaya et al. conducted on two Ephedra species (*E. alata* and *E. fragilis*) collected from Tunisia revealed that both plants had significant antioxidant activities, particularly the ethanol extract of *E. alata*, which exhibited stronger activity in comparison to the synthetic antioxidant butylated hydroxytoluene (BHT) [16].

We have found no reported paper for extracting metabolites in *E. foeminea* with different solvent extractions using maceration to investigate their metabolomic profiles and antioxidant activities. Thus, as metabolomics enables a comprehensive approach to examine a complex mixture of molecules that may be connected to observations obtained through biological activity tests and assays without the need for isolation procedures, the characterization, quantification and maximizing of active metabolites of *E. foeminea* crude extracts using different solvents of varying polarities; dichloromethane/methanol (DCM/MeOH), methanol (MeOH), ethanol (EtOH), acetone (Ace), and ethyl acetate (EA), through Nuclear Magnetic Resonance (NMR), gas-chromatography mass-spectrometry (GC–MS), and liquid-chromatography mass-spectrometry (LC–MS) coupled with an evaluation of each crude extracts’ antioxidant capacity is described. We will gain a better understanding of the properties of each extract by mapping the chemical profile and antioxidant activity of the different *E. foeminea* crude extracts. This study will employ untargeted metabolomics to examine and uncover the impact of various polar solvents on the preservation of molecules as well as the active compounds of this plant and their subsequent antioxidant activities.

## 2. Results

### 2.1. Qualitative and Quantitative Analyses of E. foeminea Crude Extracts Using Liquid-State NMR

**Figure 1:**
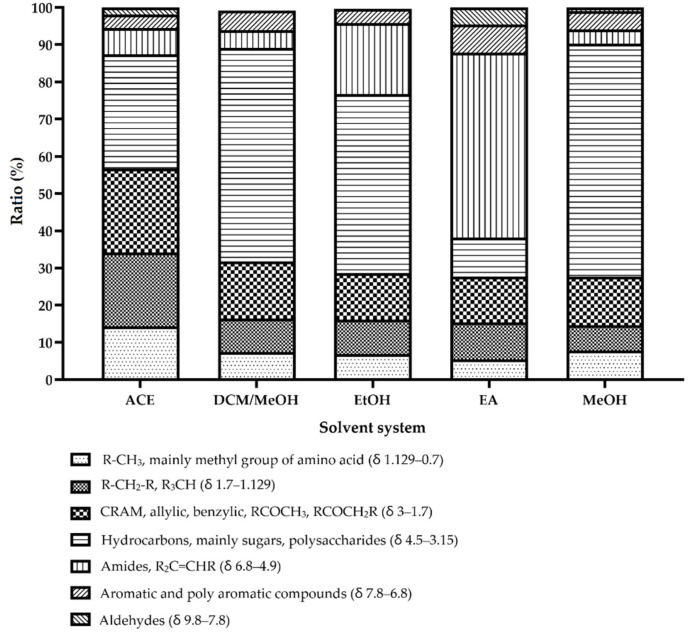
Functional group ratio comparison of different solvent extractions of *E. foeminea*. MeOH, methanolic extract; DCM/MeOH, dichloromethane/methanol extract; Ace, acetone extract; EA, ethyl; EtOH, ethanolic extract.

NMR is an inherently quantitative tool where the intensity of NMR signals is directly correlated with the concentration levels of detected molecules and the number of equivalent nuclei within the same molecule [17,18,19]. It has been routinely used for identification and for structural elucidation of molecules in mixture samples such as natural product extracts [20,21,22]. In this study, we divided the NMR spectrum into different regions associated with specific functional groups (Figure 1) and measured the analytes through signal intensities and their linear relationship with analytes concentration (%). The determination of functional groups in natural products such as heavy fuel oil, natural gas, and other similar natural products samples using NMR has been recently studied [23,24,25,26]; it has also been reported that ^1^H NMR can be used to predict the chemical and physical properties of analytes and thus functional groups [27,28,29,30]. NMR spectroscopy offers many experiments in both solid state and solution to study the chemical and structural properties of investigated molecules [31,32,33]. One of the most significant advantages of NMR is the ability to study the molecules at their atomic levels, where the chemical shift of different functional groups can be observed at different chemical shifts [34]. This is an approximate method where we estimated the CH_3_, which most likely came from amino acids in the case of plant extracts. However, that does not rule out the possibility that CH_3_ from other hydrocarbons may appear around δ 1. Nonetheless, these signals most likely come from amino acids such as leucine, valine, glycine, etc. Therefore, to validate our assumption, we utilized Chenomx Profiler (Chenomx Suite 9.0. Edmonton, AB, Canada) software as is shown in the Supplementary Figures to highlight several amino acids and polysaccharides as examples of the related peak (Figures S19–S26).

Results revealed that the different solvent extractions resulted in substantial variation in the extracted metabolites. It is well known that the water peak is observed at 4.7 ppm, which is why, in our figure, we excluded the region between δ 4.5 to 4.9 ppm. A figure showing the HDO peak is available as Supplementary Figure (Figure S29). The polysaccharides region showed the highest value at 62.59% using methanol, while ethyl acetate showed the lowest at only 10.38%, and the amino acids regional signals were the highest in the acetone extract at 14.36%. The aromatic portion of the signals was found to be quite similar across all extracts, with the highest value at 7.48% using ethyl acetate and the lowest values at 3.51% using acetone. The aliphatic region, while only making up 6.83% of the methanolic extract, was found to be the richest in the acetone extraction at 19.90%. As for carboxylic and carboxyl-rich alicyclic molecules (CRAM), the extract with highest signals was the acetone extract at 22.53% and the lowest was revealed to be the ethyl acetate extract at 12.48%. Furthermore, the amides region was significantly higher in the ethyl acetate extract compared to other extracts, comprising 49.70% of total extracted metabolites, while the extract with the lowest amides signals was the methanol extract at 3.77%. Aldehyde signals were revealed to be generally low across all extracts, where a negligible number of signals were detected in the ethanol extract and with the ethyl acetate extract spectrum displaying the most intense signals at 4.65%.

**Figure 2:**
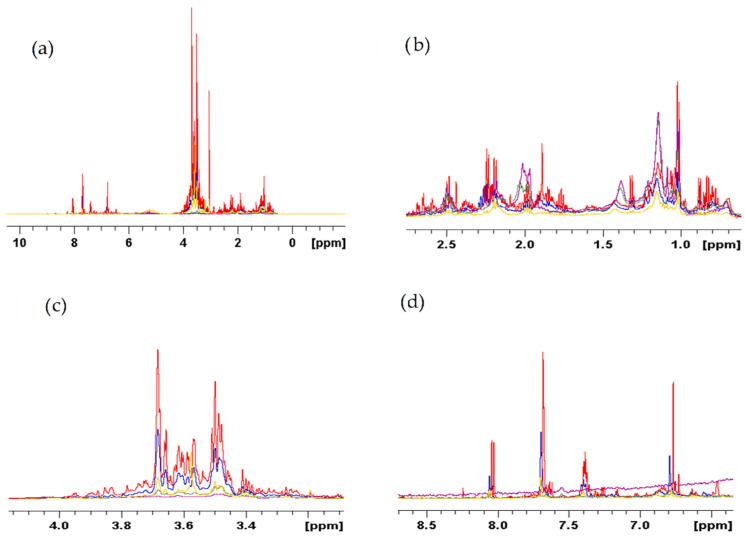
(**a**) Stack plot comparison of the 1D ^1^H spectra of different solvent extracts of *E. foeminea* recorded using 600 MHz solution NMR and dissolved in D_2_O; (**b**) extended region of CH_3_ and CH_2_ mainly CH_3_ of amino acids (δ 3–0); (**c**) polysaccharides extended region (δ 4.5–3); and (**d**) aromatic region. Yellow, ethanolic extract; purple, ethyl acetate extract; green, acetone extract; red, methanolic extract; blue, dichloromethane/methanol extract.

**Figure 3:**
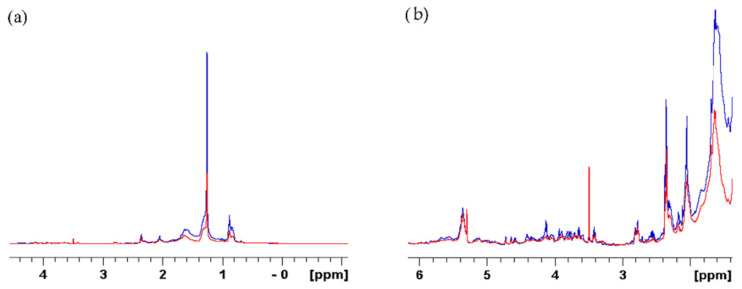
(**a**) Stack plot comparison of 1D ^1^H spectra of *E. foeminea* methanolic (red) and ethyl acetate (blue) extracts; (**b**) extended region (δ 4–3.2) dissolved in CDCl3 and recorded using 500 MHz NMR.

Figure 2a shows a 1D ^1^H spectra comparison between the five different solvent crude extracts of *E. foeminea*. Around the high-field region of δ 1.5 to 0.5 ppm, it is suggested that the methanolic extract has the largest number of amino acids. As expected, the acetone extract showed the highest peak at the lipid region around δ 1.25 as this is typically associated with the CH_2_ of lipids (Figure 3b). Figure 2c shows the polysaccharides and sugar resonance, which is present in the high to mid-field extended region of δ 4 to 3.2 ppm which clearly demonstrated that the methanolic extract had the most intense peaks in that region. The low-field region of δ 8.5 to 6.5 ppm generally shows the aromatic, substituted aromatic, phenolic, and multicyclic compound peaks. The methanolic extract showed pronounced aromatic resonance (Figure 2d) while the lipid pattern dominated the ethyl acetate extract spectrum. The ethanolic extract spectrum was found to be generally similar to the methanolic extract spectrum, which is expected from solvents with similar polarities, although the methanolic extract spectrum showed stronger intensities and more varied peaks. Individual typical ^1^H 1D spectra recorded by 600 MHz liquid-state NMR of each extract dissolved in D_2_O is shown in Supplementary Figures S1–S5.

Figure 3a shows a stack plot comparison between two *E. foeminea* extracts, methanol and ethyl acetate, spectra. The figure demonstrates that more lipids using ethyl acetate were extracted compared to the methanol extract spectrum. Figure 3b shows the extended region from δ 4 to 3.2, this is the typical polysaccharides and sugars region. Due to the region being polar, we see more pronounced resonance peaks from the methanolic extract spectrum, the more polar solvent, compared to the ethyl acetate extract spectrum. Individual typical ^1^H 1D spectra recorded by 500 MHz liquid-state NMR of each extract dissolved in CDCl3 is displayed in Supplementary Figures S6–S10.

### 2.2. Qualitative and Quantitative Analyses of E. foeminea Powder Using Solid-State NMR

**Figure 4:**
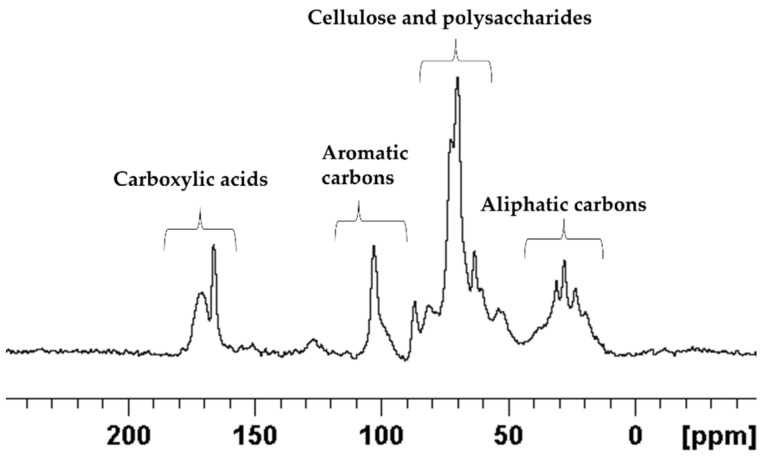
1D ^13^C spectrum of *E. foeminea* plant powder recorded using 600 MHZ solid-state NMR.

Figure 4 shows the ^13^C spectrum of solid-state NMR. The spectrum can be divided into four main regions, each corresponding to a different class of functional group: aliphatic carbons, polysaccharides and cellulose, aromatic carbons, and carboxylic phenols and carbons. As expected, the polysaccharides region is dominating the spectrum, shown in the mid to high-field region δ 90 to 45 ppm [35,36]. The high-field region from δ 45 to 10 ppm showed peaks mainly associated with aliphatic carbons. The low-field region beyond δ 90 ppm contained mainly aromatic and carboxylic resonances.

**Figure 5:**
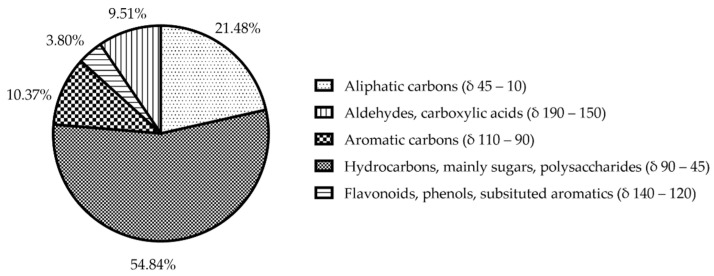
Pie diagram showcasing the signals ratio of different functional groups calculated from *E. foeminea* ^13^C solid-State NMR spectrum.

The assignments of the *E. foeminea* solid-state NMR spectrum and the calculated ratios of the groups are summarized in Figure 5.

**Figure 6:**
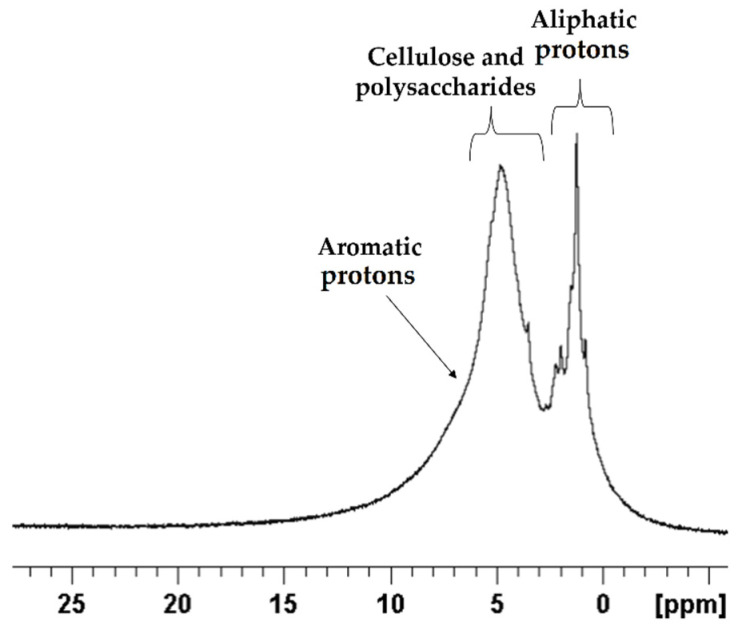
1D ^1^H spectrum of *E. foeminea* plant powder recorded using 600 MHz solid-state NMR.

To validate our findings in ^13^C solid-state NMR spectrum, ^1^H solid-state NMR spectrum was obtained using the same powder sample as shown in Figure 6.

The spectrum obtained verified the ^13^C results, where the δ 10 to 3 ppm mid-field sugars and polysaccharides region peaks are dominating the spectrum, while the aromatics region is shown as a small shoulder around δ 7.5 ppm. On the other hand, the aliphatic resonance peaks were found to be pronounced as shown in the high-field region of δ 3 ppm.

### 2.3. Chemical Profiling of E. foeminea Crude Extracts Using GC–MS and LC–MS

**Figure 7:**
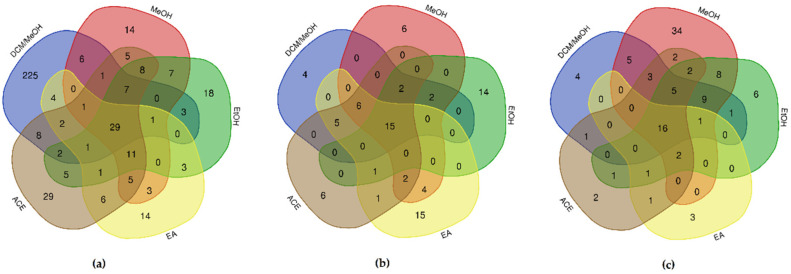
Venn diagrams showing overlapping and unique compounds of *E. foeminea* crude extracts using different solvents analyzed by (**a**) positive ion mode and (**b**) negative ion mode LC–MS scans, and (**c**) GC–MS scan. Ace, acetone; DCM/MeOH, dichloromethane/methanol; EtOH, ethanol; EA, ethyl acetate; MeOH, methanol.

The data resulting from further analysis of the *E. foeminea* crude extracts revealed a significant disparity in the number of the identified metabolites according to solvent and instrument used (Figure 7).

For example, the *E. foeminea* crude extracts analyzed by LC–MS revealed a variation between the metabolites identified in the positive ion mode scan and in the negative ion scan mode. The positive ion scan LC–MS data revealed a total of 401 metabolites in the DCM/MeOH crude extract sample, 159 metabolites in the Ace sample, 132 metabolites in the MeOH sample, 130 metabolites in the EtOH sample, and 107 in the EA sample (Supplementary Table S1). Unique compounds were detected using ESI+ mode LC–MS as a combined total of 300 metabolites in all five extracts, with the DCM/MeOH sample displaying the largest amount of identified unique compounds at 225 metabolites. The samples also had 29 identified metabolites in common (Figure 7a). The unique and shared compounds are listed in Supplementary Table S2. The major constituents of each extract analyzed by ESI+ mode LC–MS, determined by calculating the peak area (%), are described in Table 1b. Typical positive mode LC–MS total ion chromatograms (TIC) of different *E. foeminea* crude extracts are shown in Supplementary Figure S11.

Furthermore, data acquired through negative ion LC–MS scan of the five extracts detected 62 metabolites in the EA sample, 46 metabolites in the Ace sample, 43 metabolites in the MeOH sample, 41 metabolites in the DCM/MeOH sample, and 39 metabolites in the EtOH sample (Table S3). A sum of 45 unique metabolites were detected in all five extracts, where the highest number of identified unique compounds was found in the EA crude extract sample at 15 metabolites. Meanwhile, the total number of common metabolites shared by all the samples was also found to be 15 metabolites (Figure 7b). The unique and shared compounds analyzed by negative ion LC–MS are listed in Supporting Material (Table S4). The major constitutes of each extract analyzed by negative ion LC–MS are described in Table 1a, determined by calculating the peak area (%). Typical negative mode LC–MS TIC of different *E. foeminea* crude extracts are shown in Supplementary Figure S12.

Derivatized (TMS) metabolites were also tentatively identified in the *E. foeminea* crude by GC–MS scan; however, some compounds could not be determined by mass spectral matching due to the match being less than 65%. Results revealed a difference in the number of identified metabolites; for instance, a total of 113 metabolites were tentatively identified in the *E. foeminea* MeOH crude extract sample, followed by 66 metabolites in the EtOH extract sample, 58 metabolites in the DCM/MeOH extract sample, 47 metabolites in the Ace extract sample, and 31 metabolites in the EA extract sample (Table S5). The combined number of unique compounds detected by the GC–MS scan in all extracts totaled 49 compounds, with the MeOH extract sample containing the largest number of unique compounds at 34 (Table S6). Conversely, the extracts collectively shared 16 of the identified compounds. The main constituents of each extract were determined by calculating the peak area (%) Table 2 Typical GC–MS TIC of derivatized *E. foeminea* crude extracts using different solvents are exhibited in Supplementary Figure S13.

### 2.4. Untargeted Metabolomics Study of E. foeminea Crude Extracts

**Figure 8:**
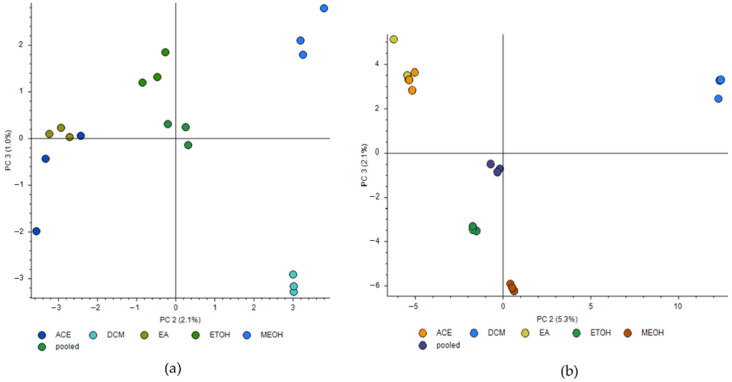
Principal component analysis (PCA) score plots of different solvent *E. foeminea* crude extract LC–MS data per injection. (**a**) negative mode scan; (**b**) positive mode scan. Ace, acetone extract; DCM/MeOH, dichloromethane/methanol extract; EA, ethyl acetate; EtOH, ethanol; MeOH, methanol. Samples were analyzed in triplicates.

The untargeted metabolomics profile of the different solvent *E. foeminea* crude extracts was performed using GC–MS and LC–MS. The data obtained from positive and negative ion LC–MS scans were subjected to the principal component analysis (PCA) in order to underline the *E. foeminea* crude extracts’ similarities and differences based on their chemical compositions (Figure 8). In this regard, a matrix containing the five different solvent crude extract samples and one pooled sample, and six variables for each sample according to the solvent used was computed. Analysis was carried out in triplicates, where the pooled samples clustered in the middle in both PCA plots. The second and third principal components (PC2 and PC3) were chosen. In the negative mode scan, the second and third principal components explained 2.1% and 1.0% of the variation, respectively (Figure 8a). The solvent extracts EtOH, EA, and Ace clustered roughly on one side of the scatter plot; meanwhile, the MeOH and DCM/MeOH extracts were highly separated from each other as well as from the other three extracts. The positive scan mode score plot showcased some differences where the second component explained 5.3% of the variation, while the third component explained 2.1% (Figure 8b). The Ace and EA extracts were very closely clustered, while the EtOH, and DCM/MeOH, and MeOH extract samples had distinct features compared to the other extracts, with the latter clustering more closely to the EtOH extract sample.

**Figure 9:**
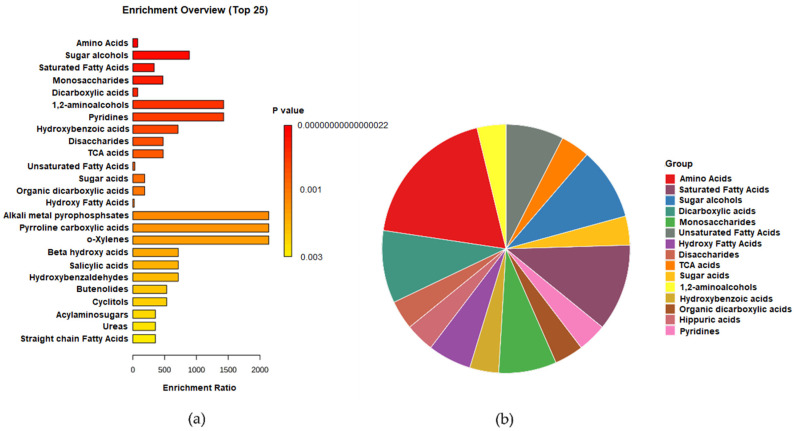
(**a**) Over Representation Analysis (ORA) Bar chart and (**b**) pie chart of the chemical classification of the total metabolites identified in the five *E. foeminea* crude extract samples using GC–MS via metabolite set enrichment analysis (MSEA). Colors in the bar plot describe the *p*-value. The red and orange colors signify the high and low values, respectively. The lines indicate the enrichment ratio, which was computed by hits/expected, where hits = observed hits and expected = expected hits. The colors in the pie chart designate each chemical group relative to the total number of compounds.

A total of 320 compounds were identified in all samples using ESI+ mode GC–MS scan. Metabolite set enrichment analysis (MSEA) was conducted to classify the chemical groups of all identified compounds using GC–MS (Figure 9). The bar chart represents the chemical classifications of the identified metabolite sets (top 25) under the chemical structure metabolite set library category (Figure 9a). As shown in the bar chart, among the top 25 chemical classes, the primary chemical groups with a higher *p*-value were mainly amino acids, sugar alcohols, saturated fatty acids, monosaccharides, dicarboxylic acids, 1,2-aminoalcohols, pyridines, hydroxy benzoic acids, disaccharides, and trichloroacetic acids. The colors in the pie chart designate each chemical group relative to the total number of compounds (Figure 9b). Among the 15 chemical groups, the highest number of compounds were mainly described by amino acids (red), saturated fatty acids (deep purple), sugar alcohols (blue), dicarboxylic acids (turquoise blue), monosaccharides (green), unsaturated fatty acids (grey), and hydroxy fatty acids (violet), consecutively. Detailed results of this analysis are displayed in Supplementary Table S7. Individual MESA pie charts and ORA bars analysis of each extract is shown in Supplementary Figure S14–S18. Alongside the detailed results shown in Supplementary Table S8.

The described top compound classes were detected in some extract samples but were found to be absent in others (Table 3).

### 2.5. Effect of E. foeminea Crude Extracts on ABTS Antioxidant Assay

**Figure 10:**
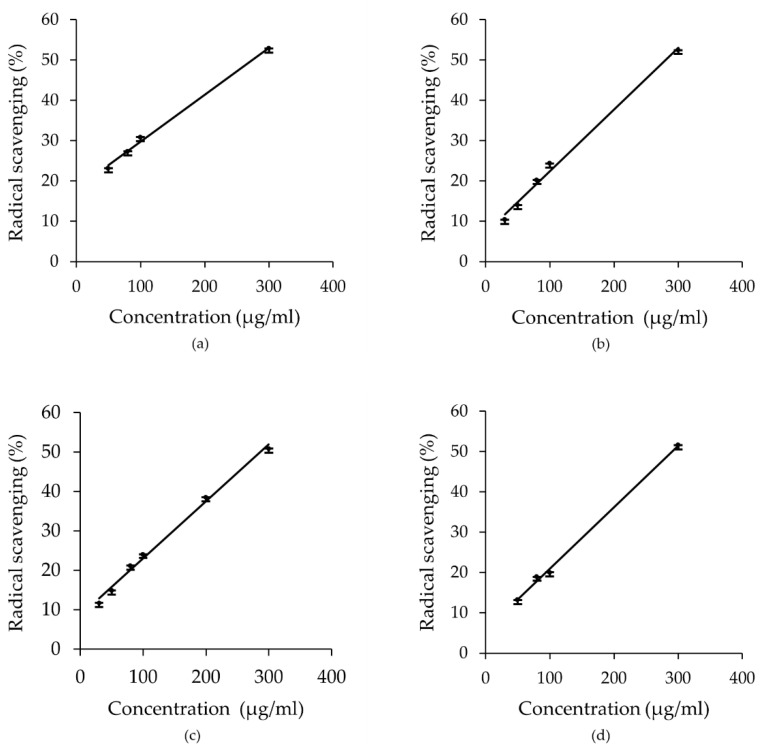

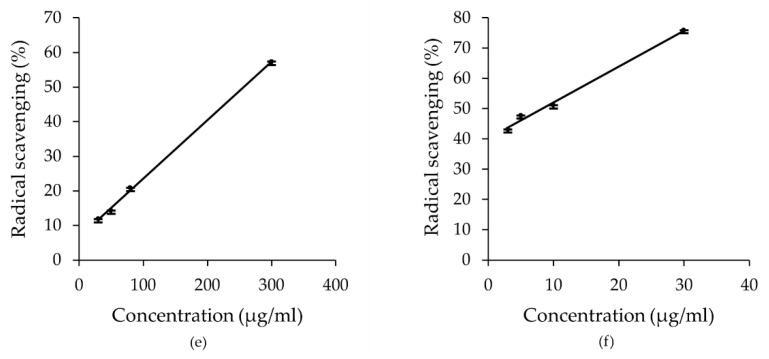
Dose-dependent ABTS (%) radical scavenging activity of *E. foeminea* crude extracts. (**a**) Acetone; (**b**) dichloromethane/methanol; (**c**) ethanol; (**d**) ethyl acetate; (**e**) methanol; (**f**) ascorbic acid as control. Pearson correlation coefficients (r) between concentrations and % radical scavenging activity were found to be 0.9735, 0.9969, 0.9970, 0.9988, and 0.9995, respectively. Values are the means of triplicate analyses; error bars denote the relative standard deviation.

Several concentrations (30–300 µg/mL) of the different solvent extracts of *E. foeminea* were tested for antioxidant activity. Dose dependent calibration curves were constructed and carried out and IC_50_ values were determined for each extract (Figure 10). The ABTS radical scavenging effects of the different solvent extracts are presented, alongside ascorbic acid as the positive control. The lower the IC_50_ value of plant extracts used, the higher was their free radical scavenging activity. Antioxidant activity for *E. foeminea* crude extracts was observed in the following order: methanol extract (IC_50_ 249.6 µg/mL) > acetone extract (IC_50_ 275.2 µg/mL) > dichloromethane/methanol extract (IC_50_ 280 µg/mL) > ethanol extract (IC_50_ 285.9 µg/mL) > ethyl acetate extract (IC_50_ 289.8 µg/mL). The IC_50_ values for all five extractions of *E. foeminea* were generally within a similar range at (249.6 to 289.8 µg/mL), and the IC_50_ of the pure ascorbic acid standard was significantly lower than all tested extracts (IC_50_ 5.974 µg/mL), which indicates that the plant possesses moderate to low antioxidant ability, with the methanolic extraction having the lowest IC_50_ value at 249.6 µg/mL.

## 3. Discussion

Plants native to the Eastern Mediterranean region have long been used in medicine [37]. However, few studies have been conducted to investigate the antioxidant activity and chemical composition of medicinal plants in that region, specifically in the Arab regions of the East Mediterranean. Our study aimed to obtain a comprehensive snapshot of *E. foeminea* chemical composition and detect bioactive metabolites with reported antioxidant potential. Therefore, we subjected the different solvent crude extracts to extensive chemical analysis and metabolomics study using GC–MS, LC–MS, and NMR instruments. Many studies have reported the impact of different solvents on the content of secondary metabolites and/or their antioxidant activity [10,11,38]. NMR spectroscopy offers a potent and versatile analytical platform that has been widely used for molecular identification and quantification [17]. Several advantageous features, such as non-destructive and non-biased methods, make NMR spectroscopy superior in investigating the specific and general chemical composition of studied samples, as a ^1^H NMR spectrum provides a single snapshot of all the detectable compounds present in the sample.

Although liquid-state NMR is very informative in showing this ratio of the chemical composition of the extracted samples, there are several molecules that play an interesting functional and structural role in the plant. However, some of these important compounds can be insoluble in solvents; thus, we decided to run solid-state NMR for the plant powder alongside liquid-state NMR for the different extracts. To the best of our knowledge, this is the first time that solid-state NMR is used to evaluate the chemical composition of *E. foeminea*. Visual inspection of the typical liquid-state NMR spectra revealed obvious visual differences between the different solvent extracts of *E. foeminea*, especially in the methanolic extract spectrum where the most pronounced and varied peaks were seen, suggesting a variation in metabolic makeup according to the solvent used. Generally, the results obtained from both analyses have shown that the polysaccharides region dominated the spectra; most likely attributed to cellulose, which is known as the main structural fiber in plants. Furthermore, elevated carboxylic, phenolic, and substituted aromatic associated signals were detected, particularly in the methanolic extract, indicating that *E. foeminea* possesses bioactive molecules such as flavonoids, alkaloids, and phenolic compounds, among others. These findings were found to be in accordance with previous reports [5]. Ultimately, our liquid-state NMR analyses results indicated that the methanolic extract showed the most variance in composition compared to the other extracts.

To further investigate and maximize the detection of these molecules, ESI+ mode GC–MS and ESI+, ESI− mode LC–MS analyses of *E. foeminea* crude extracts were conducted. Generally, our major constitute analyses implied that fatty acids, particularly palmitic acid, and carbohydrates are the majority metabolites in *E. foeminea*. A Venn diagram is a schematic representation of the elements in a set or a group, where it demonstrates all the possible relationships between a finite assemblage of sets or groups. Our analysis revealed variations in the extracted metabolites according to the solvent used, as well as demonstrated differences and similarities between each extract. A total of 521 metabolites were identified in DCM/MeOH extract, 272 in the MeOH extract, 260 in the Ace extract, 258 in the EtOH extract, and 195 in the EA extract. The DCM/MeOH extract was shown to extract the most unique compounds in the LC–MS Venn analyses. These results suggest that utilizing a solvent mixture is advantageous in maximizing the extraction of metabolites with varying structures and capabilities, due to the difference in polarities. Similarly, the GC–MS Venn analysis results demonstrated that MeOH extracted a large number of unique compounds in comparison to the other extracts, indicating that methanol is an efficient solvent for extracting diverse plant metabolites. Furthermore, the qualitative screening of the five crude extracts demonstrated that the MeOH extract contained the most varied number of chemical classes. The results also confirmed the presence of different known bioactive metabolites which may have contributed to the antioxidant capacity of *E. foeminea*.

PCA is a projection method used for overviewing and explaining clusters and trends within multivariate data where the resulting score plot highlights clustering or pattern formations in a 2D space, which provides a view of similarities and dissimilarities between samples. The PCA score plots also further confirmed variations in the chemical profiles of the *E. foeminea* crude extracts according to the solvent used. The plots suggest a close similarity in the chemical composition of the acetone and ethyl acetate extracts, while the dichloromethane/methanolic extract and the methanolic extract were found to be highly separated. This implies the possession of distinctive features and further supports our findings indicating a diverse chemical makeup in both these extracts.

Metabolite Set Enrichment Analysis (MESA) is a method to identify biologically meaningful patterns that are significantly enriched in quantitative metabolomic data. Inputting the acquired GC–MS identified compound name lists revealed that amino acids were among the largely identified compound classes in the *E. foeminea* extracts, followed by saturated fatty acids. This supports our previous findings. Several of the identified amino acids have previously been reported to possess protective antioxidant and radical scavenging abilities, including valine, serine, leucine, proline, threonine, and γ-aminobutyric acid (GABA), a naturally occurring non-protein amino acid [38,39,40,41,42]. Our analysis also identified several polyols with antioxidant capabilities; mannitol, myo-inositol, and meso-erythritol, all of which have not yet been reported in Ephedra species, to the best of our knowledge. Mannitol is a polyol and a known diuretic that was recently proposed to be an oxygen radical scavenger, myo-inositol is a naturally occurring sugar alcohol that has demonstrated superoxide scavenging abilities, and Meso-erythritol has been revealed to be an excellent OH radical scavenger [43,44,45]. Other previously reported carboxylic acids in Ephedra species with potential antioxidant abilities were detected in our study; they included kynurenic, malic, oxalic, and citric acid. Succinic acid and azelaic acid were also detected in this study; however, their detection had not yet been reported in Ephedra species [5,46,47,48]. Another highly identified class was saturated and unsaturated fatty acids. Among the identified fatty acids was oleic acid, which exhibited a strong DPPH radicals scavenging activity in a recent study [49], as well as myristic acid, which reportedly had antioxidant, COX-I, and COX-II enzymes inhibitory activities [50]. These findings are in line with previous reports on Ephedra species including *E. foeminea* [13,51]. However, our study identified another fatty acid with antioxidant capabilities, α-eleostearic acid, which had not yet been detected in previous Ephedra studies [52]. Several vitamins were also detected in *E. foeminea* as potential antioxidant metabolites, among them was pantothenic acid (vitamin B5), which had not yet been reported in the Ephedra species. A study published in 2020 demonstrated that pantothenic acid, among other molecules in the leaves and petioles of four celery cultivars, contributed significantly to O2—scavenging activity [53]. Lower levels of ascorbic acid (vitamin C), which is well-known for its powerful radical scavenging activities [54], were also detected in our *E. foeminea* crude extracts. A study by Harisaranraj et al. had previously reported the identification of ascorbic acid in *E. vulgaris*, another member of the Ephedra species [55]. The Ephedra species is also well known for its vast alkaloid content. Among the alkaloids which showed some antioxidant activity detected in this study are trigonelline and stachydrine [56,57], both of which had not yet been reported in *E. foeminea*. Phenolic compounds, which are well-known for their strong radical scavenging abilities, were detected in *E. foeminea* through GC–MS and LC–MS analyses. They included benzoic, salicylic and protocatechuic acid, coumarin, kaempferol, caffeic acid, and o-coumaric acid, which were previously reported in several Ephedra species [58,59,60], as well as phloretin, shogaol, and vitexin 2″-O-p-coumarate, which are reported for the first time in this study. These compounds have all been shown to possess antioxidant and radical scavenging abilities in past studies [61,62,63,64,65,66]. However, a study by Ibragic and Sofić found that *E. foeminea* had the lowest total phenolic content (TPC), and had among the lowest total alkaloids content (TAC) and total flavonoids content (TFC) compared to the other tested Ephedra species [5]. A diverse number of antioxidant terpenoids have also been identified in our study, some of which have been detected in *E. sinica* in a previous study by Qingbiao et al., such as β-ionone, cumin aldehyde, geranyl acetate, and p-cymene [67]. Other detected terpenoids with antioxidant potential included andrographolide and cafestol, both of which have yet to be reported in previous studies [68,69]. Our analysis also revealed the presence of pheophorbide A and pheophorbide B in all the extracts, which had not been previously reported in *E. foeminea* or any other Ephedra study. Pheophorbides are plant products related to the chlorophyll molecule and has been reported to possess potential antioxidant activities [70,71].

The crude extracts obtained from different solvents were studied for their antioxidant activity by using ABTS scavenging activity assays. The methanolic extract showed the highest potency expressed as IC_50_ value of ABTS scavenging activity (249.6 µg/mL). This could be linked to the presence of pheophorbide A and kynurenic acid as major constituents of the methanolic extract, as well as the possibility that methanol extracted the highest level of phenols, flavonoids, alkaloids, and terpenoids as differences in the polarity of the extraction solvents could cause a wide variation in the level and class of bioactive compounds in the extract [72,73]. However, no significant differences were found in the ABTS radical scavenging activity of the five *E. foeminea* crude extracts. The DPPH, ABTS, FRAP antioxidant activity expressed as Trolox equivalents of *E. foeminea* collected from South Lebanon was reported in a recent study, where the radical scavenging activities varied according to solvent system used [74]. Our results concluded that methanol is the most relatively efficient solvent for extracting diverse bioactive metabolites from *E. foeminea*. More research should be conducted to optimize the identification and isolation of bioactive compounds from *E. foeminea.*

## 4. Materials and Methods

### 4.1. Plant Material

Whole plant samples of *Ephedra foeminea* were identified and collected from Jordan in 2013. The dried plant sample was ground using an electrical grinder into fine powder, then labeled and stored in an air sealed plastic bag at −20 °C.

### 4.2. Preparation of E. foeminea Crude Extracts

Four grams of *E. foeminea* plant powder was macerated on a shaker overnight (approximately 17 h) at room temperature in 40 mL of each different solvent; dichloromethane/methanol (1:1) mixture was prepared from methanol (purity ≥ 99.8%) and dichloromethane (purity ≥ 99.8%) purchased from Honeywell Riedel-de Haen (Seelze, Germany), ethyl acetate (purity ≥ 99.8%) purchased from VWR Chemicals (Radnor, PA, USA), acetone (purity ≥ 99.8%) purchased from Fisher Chemical (Waltham, MA, USA), ethanol (purity ≥ 99.8%), and methanol (purity ≥ 99.8%) purchased from Honeywell Riedel-de Haen. The extracts were centrifuged the next day at 14,000× *g* for 2 min to pellet down all solid material, and the supernatant was transferred into fresh, properly labeled tubes. Centrifugation is a more efficient and a less time- and resource-consuming method of separating extracts from particles, it is an accelerated form of sedimentation. This process was repeated a few times to obtain clear crude extracts. The extracts were then stored at −20 °C.

### 4.3. NMR Analyses of E. foeminea

#### 4.3.1. Liquid-State NMR Analysis

To prepare NMR samples for each extract, 1.5 mL of the crude extracts was transferred to 2 mL Eppendorf tubes using pipettes, then dried using speed vacuum for approximately 14 h. Each NMR sample was prepared by dissolving the dried extract in 600 µL of deuterium oxide (purity 99.9 Atom % D) purchased from Sigma-Aldrich (Saint Louis, MO, USA) in 2 mL Eppendorf tubes, vigorously vortexed for 30 s, then 550 µL of the solution was transferred to 5 mm NMR tubes. NMR spectra were recorded using a Bruker 600 MHz AVANACE III NMR spectrometer equipped with a Bruker BBOF probe (BrukerBioSpin, Rheinstetten, Germany). For quantitative analysis, all ^1^H NMR spectra were recorded by collecting 128 scans with a recycle delay time of 5 s using one 90° pulse sequence (zg) program from the Bruker pulse library. To create comparable data, all liquid-state NMR spectra were recorded under the same conditions using identical parameters. Chemical shifts were adjusted using the 4,4-dimethyl-4-Silapentane-1-Sulfonic acid (DSS) signal at 0.0 ppm as an internal chemical shift reference. The pulse duration was set to 10.5 microseconds at 35.5 watts with the spectral width set to 16 ppm, digitized with 32K points. The FID signals were amplified by an exponential line-Broadening factor of 1–5 Hz before Fourier transformation. Before recording the integration values, the phase of each spectrum was corrected manually, and the baseline was adjusted automatically using the “abs n” command.

NMR spectra of the samples dissolved in Chloroform D (purity 99.8 atom % D) purchased from Sigma-Aldrich were recorded using a Bruker 500 MHz AVANACE III NMR spectrometer equipped with Bruker CPTCI multinuclear CryoProbe (BrukerBioSpin, Rheinstetten, Germany) operating on fully automated mode. The ^1^H NMR spectrum was recorded by collecting 128 scans with a recycle delay time of 5 s, using one pulse sequence through a standard (zg) program from the Bruker pulse library. Chemical shifts were corrected using Tetramethylsilane (TMS) signal at 0.0 pp, as internal chemical shift reference. The duration of 90 pulse is optimized to 12 microseconds at 13.734 watts. The phase of each spectrum was corrected manually, and the baseline was adjusted automatically using “abs n” command. DSS was used for the analyses an internal reference (0.5 mM) as recommended in literature [75].

#### 4.3.2. Solid-State NMR Analysis

For solid-state NMR analysis, plant powder was packed evenly into 4 mm zirconia rotor and sealed at the open end with a Vespel cap. The ^13^C solid-state NMR spectra were acquired using Bruker 600 MHz AVANACE III spectrometer equipped with a 4 mm double resonance MAS Bruker Probe (BrukerBioSpin, Rheinstetten, Germany). The spectrum was measured with a 14 kHz spinning rate using cross polarization experiment cp pulse program from the Bruker pulse library with 3ms contact time and recycle delay time of 5 s. To achieve high signal to noise ratio, the spectra were recorded by collecting at least 18k scans with recycle delay time of 30 s. The chemical shifts were corrected using adamantane signal at δ 37.7 for ^13^C spectra and δ 1.7 for ^1^H spectra as external chemical shift reference. The ^1^H NMR spectrum was recorded using a Bruker 600 MHz AVANACE III NMR spectrometer equipped with a 3.2 mm Bruker MAS probe (BrukerBioSpin, Rheinstetten, Germany). The spectrum was recorded with 22 kHz and 20 kHz spinning rate using adiabatic double echo refocusing (zgse.ajr) pulse program with recycle delay time of 5 s. The functional group ratios (%) were calculated for each extract based on the total sum of the following spectral integrals: (a) δ 0.7–1.129, (b) δ 1.129–1.7, (c) δ 1.7–3, (d) δ 3.15–4.5, (e) δ 4.9–6.8, (f) δ 6.8–7.8, and (g) δ 7.8–9.8.

### 4.4. GC–MS Analysis of E. foeminea Crude Extracts

Extract samples were centrifuged for 5 min at 4 °C and 14,000× *g* to remove particles, then transferred 10 μL of the supernatants in fresh tubes and dried in speed vacuum. In a fume hood, 30 μL of MOX reagent (methoxyamine HCl in pyridine, 15 mg/mL) was added to the dried samples and thermomixed (mixed with heat) at 30 °C and 600 rpm for 90 min. For extract derivatization, 50 μL of a mixture of BSTFA spiked with n-alkanes (C 8 to C 40) purchased from Supelco was added as an internal standard and thermomixed at 37 °C and 600 rpm for 30 min to form trimethylsilyl (TMS) derivatives. The samples were then centrifuged at room temperature for 5 min at 14,000× *g* to sediment any particles, and 30 μL of the supernatants was then transferred into amber GC vials for analysis.

The triple quadrupole mass spectrometer system (Agilent 7010B) was coupled with GC (Agilent 7890). The electron ion (EI) source was operated at electron energy of 70 ev with temperature set at 230 °C and mass analyzer temperature set at 150 °C. The scan range was set 50–700 Da with scan time of 106 ms and the GC inlet and transfer line temperatures were set at 250 °C and 320 °C, respectively. Helium was utilized as the carrier gas at a constant flow rate of 1.0 mL min^−^^1^. The derivatized solution of each sample (1 µL) was injected in a splitless mode into the GC inlet using an autosampler and analyzed in full MS scan with a solvent delay of 8 min. Compounds were separated on a DB-5MS fused silica capillary column (30 m × 0.25 mm I.D., 0.25-µm film thickness; Agilent J&W Scientific, Folsom, CA, USA) with 5% phenyl methylpolysiloxane cross-linked stationary phase. The initial oven temperature was held at 70 °C for 4 min, then ramped to 330 °C at a rate of 6.0 °C min^−1^, and, finally, held at 330 °C for 5 min. The total analysis time was 52 min.

### 4.5. LC–MS Analysis of E. foeminea Crude Extracts

The untargeted screening and metabolomics study of the five different solvent *E. foeminea* crude extracts were performed using ultra-high-pressure liquid chromatography coupled to Orbitrap ID-X mass spectrometer (UHPLC-Orbitrap ID-X MS). Samples were prepared for analysis by transferring 0.5 mL of each extract into an Eppendorf tube and completely dried under speed vacuum before reconstituting the dried extracts in 1 mL of Acetonitrile (purity ≥ 99.9%) purchased from Sigma-Aldrich for analysis. Reserpine was used as ACL quality control. The Orbitrap ID-X is a mass spectrometer that contains three mass analyzers, which was used to analyze the mass-to-charge ratio (*m*/*z*) of the studied molecules. The Orbitrap IDX spectrometer could reach a high resolution (>120,000) and reliable mass accuracy (<3 ppm mass error). The Mass spectrometer was calibrated using a purchasable “Calibration Mix ESI (Thermo Scientific)” in accordance with the manufacturer’s guidelines. Electrospray ionization in positive (ESI+) and negative (ESI−) modes were applied for the studied compounds and the following parameters were applied: vaporized temperature = 100 °C, voltage = 3500 V, sheath gas = 30, auxiliary gas: 15, ion source fragmentation = 35 V, and capillary temperature = 300 °C. An amount of 10 µL of each sample was injected through a loop injection to a C18 column (Acquity CSH 100 × 2.1 mm, 1.7 µm) using an independent UPLC pump. The samples were automatically infused (5 µL each) through the UHPLC system with the use of the C18 column for the separation. The flow rate was set to 0.5 mL/min and a gradient of 15 min was applied for the separation.

### 4.6. The 2,2′-azino-bis(3-ethylbenzothiazoline-6-sulfonic acid (ABTS) Radical Cation Assay of E. foeminea Crude Extracts

ABTS (Diammonium 2,2′-Azinobis [3-ethyl-2,3-dihydrobenzothiazole-6-sulfonate]) Ultra Pure (purity ≥ 98.5%) purchased from VWR Chemicals and potassium persulfate (purity ≥ 99.0%) purchased from Honeywell Fluka were dissolved in deionized water to make a 7 mM solution and 2.45 mM solution, respectively. To produce the ABTS free radical cation, the solutions were mixed and incubated in the dark at room temperature for 12 to 16 h. The free radical solution of ABTS was diluted before use with ethanol (purity ≥ 99.8%) purchased from Riedel-de Haen to an absorbance of 0.7 at 734 nm for the assay. Reaction mixtures of 200 µL volume were formed in the wells of 96 well microplates, by mixing 10 µL of each plant extract in different concentrations with 190 µL diluted ABTS working solution as triplicates and incubated for 10 min in the dark at room temperature. After incubation, the absorbance was recorded at 734 nm. The results were compared with the control wells which contained 200 µL of the diluted ABTS working solution instead of plant extracts. The positive control was ascorbic acid in concentrations (1, 3, 5, 10, and 30 µg/mL). The ABTS radical scavenging was calculated as percent (%) disappearance of the deep blue color as follows:

{(absorbance of control − absorbance of samples)/absorbance of the control} × 100.

The IC_50_ of the plant extracts were calculated from a linear regression curve of (%) radical scavenging against concentrations of the extracts (µg/mL), and extrapolation from the equation of the curve, y = mx + c. Where y is IC_50_ = 50, m is the gradient of the curve, x is the concentration and c is the intercept on y axis.

### 4.7. Statistical and Multivariate Analysis and Data Processing

For LC–MS, AcquireX Deep Scan on pooled sample workflow was used as an intelligent data-dependent Automated MSn data acquisition of all precursor ions. Automated AcquireX background exclusion and inclusion lists were generated. MS/MS data were automatically acquired on the pooled sample for identification only for comprehensive fragmentation of sample relevant compounds. Compound discoverer version 3.1 (Thermo Fisher Scientific, Bremen, Germany) was used to treat and process the LC–MS data using the following workflow: Untargeted Metabolomics workflow to find and identify the differences between samples, visualized by the multivariate statistical tool PCA. Retention time alignment and unknown compound detection was performed using mzcloud spectral library (ddMS2) and local compound databases (exact mass or formula). Metabolite set enrichment analysis (MSEA) including Over Representation Analysis (ORA) carried out using on GC–MS data using MetaboAnalyst 5.0 online software (https://www.metaboanalyst.ca/MetaboAnalyst/home.xhtml, accessed on 20 February 2022) based on enrichment analysis by inputting the compound names lists, which were identified through raw data processing by the open-access metabolomics software MSDIAL 4.80 (http://prime.psc.riken.jp/compms/msdial/main.html, accessed on 20 February 2022). The raw data was converted from the original file format (.raw) into the common file format (.abf) which could better match the MSDIAL software by a Reifycs Abf (Analysis Base File) Converter. Moreover, the compound name lists obtained through MSDIAL were also analyzed using Venn diagrams via the open-source Venn diagram creation tool (https://bioinformatics.psb.ugent.be/webtools/Venn/, accessed on 19 March 2022). Agilent MassHunter Workstation software (B.08.00) was used to tentatively identify any remaining unknown compounds from the GC–MS data by matching the spectra to those stored in National Institute of Standards and Technology library (NIST14). ChemOnt (ClassyFire) (http://classyfire.wishartlab.com/tax_nodes, accessed on 22 April 2022) ontology was used to classify and reference chemical classes. Bruker Topspin 3.5pl7 software (Bruker BioSpin, Rheinstetten, Germany) was used for ^1^H and ^13^C NMR data collection and spectral post-processing. Pearson correlation coefficients (r) between concentrations and % radical scavenging activity were calculated. The graphical abstract was created using web-based application BioRender (https://www.biorender.com, accessed on 24 April 2022). GraphPad Prism version 9.3. for Windows (GraphPad Software, San Diego, California USA) was used to create figures. Chenomx Profiler (Chenomx Suite 9.0, Edmonton, AB, Canada) software was used to highlight several NMR peak assignments.

## Figures and Tables

**Table 1:** Major constitutes of different solvent *E. foeminea* crude extracts analyzed by (a) ESI− and (b) ESI+ mode LC–MS.

**(a) ESI–**						
**S. No.**	**Compound Name**	**RT (Min)**	**Formula**	**M/Z**	**Peak Area (%) ^1^**	**InChI Key**
Ethanol extract						
1	(9*Z*)-(13*S*)-12,13-Epoxyoctadeca-9,11-dienoicacid	10.809	C_18_H_30_O_3_	294.2198	16.82	ZFVKKBAQVWQQHP-ALADIWIOSA-N
2	(9*Z*)-(12*S*,13*R*)-12,13-epoxyoctadecenoic acid	10.578	C_18_H_32_O_3_	296.2354	13.195	CCPPLLJZDQAOHD-GJGKEFFFSA-N
3	8-Hydroxyluteolin 4′-methyl ether 7-(6′-acetylallosyl) (1->2) (6′-acetylglucoside)	9.179	C_32_H_36_O_19_	724.1829	10.772	ZXQCHXLLIKMUTB-WIFPZZFDSA-N
4	(9*Z*,15*Z*)-(13*S*)-12,13-Epoxyoctadeca-9,11,15-trienoicacid	10.277	C_18_H_28_O_3_	292.2042	8.94	YZBZORUZOSCZRN-DCUPSMFCSA-N
5	Pregna-4,9(11)-diene-3,20-dione	11.455	C_2_^1^_H28_O_2_	312.2093	5.052	LCXMRSLFWMMCAS-WRJHFWDFSA-N
6	(7*S*,8*S*,9*Z*,12*Z*)-7,8-dihydroxyoctadeca-9,12-dienoic acid	10.811	C_18_H_32_O_4_	312.2305	4.518	NMONGVDUESEHOK-MPOZZNMKSA-N
7	Hexadecanedioic acid	7.543	C_16_H_30_O_4_	286.2146	3.635	QQHJDPROMQRDLA-UHFFFAOYSA-N
8	9,10,13-trihydroxy-11-octadecenoicacid	7.418	C_18_H_34_O_5_	330.2411	3.445	NTVFQBIHLSPEGQ-BUHFOSPRSA-N
9	(9*Z*)-(13*S*)-12,13-Epoxyoctadeca-9,11-dienoicacid	10.075	C_18_H_30_O_3_	294.2198	3.254	ZFVKKBAQVWQQHP-ALADIWIOSA-N
10	(9*Z*)-(13*S*)-12,13-Epoxyoctadeca-9,11-dienoicacid	10.008	C_18_H_30_O_3_	294.2198	2.933	ZFVKKBAQVWQQHP-ALADIWIOSA-N
11	[(2*S*,3*R*,4*S*,5*S*,6*R*)-2-[5,7-dihydroxy-2-(4-hydroxyphenyl)-4-oxochromen-8-yl]-4,5-dihydroxy-6-(hydroxymethyl)oxan-3-yl] (E)-3-(4-hydroxyphenyl)prop-2-enoate	7.8	C_30_H_26_O_12_	578.1448	2.71	FJGOEBQRHWKKJH-HORBVDEJSA-N
Ethyl acetate extract						
1	(9*Z*)-(12*S*,13*R*)-12,13-epoxyoctadecenoic acid	10.557	C_18_H_32_O_3_	296.2354	11.215	CCPPLLJZDQAOHD-GJGKEFFFSA-N
2	8-Hydroxyluteolin 4′-methyl ether 7-(6′-acetylallosyl) (1->2) (6′-acetylglucoside)	9.158	C_32_H_36_O_19_	724.1828	10.9	ZXQCHXLLIKMUTB-WIFPZZFDSA-N
3	(9*Z*)-(13*S*)-12,13-Epoxyoctadeca-9,11-dienoicacid	10.788	C_18_H_30_O_3_	294.2198	10.268	ZFVKKBAQVWQQHP-ALADIWIOSA-N
4	(9*Z*,15*Z*)-(13*S*)-12,13-Epoxyoctadeca-9,11,15-trienoicacid	10.261	C_18_H_28_O_3_	292.2041	7.105	YZBZORUZOSCZRN-DCUPSMFCSA-N
5	(9*Z*)-(13*S*)-12,13-Epoxyoctadeca-9,11-dienoicacid	10.741	C_18_H_30_O_3_	294.2198	3.744	ZFVKKBAQVWQQHP-ALADIWIOSA-N
6	Hexadecanedioic acid	7.527	C_16_H_30_O_4_	286.2146	3.195	QQHJDPROMQRDLA-UHFFFAOYSA-N
7	[(2*S*,3*R*,4*S*,5*S*,6*R*)-2-[5,7-dihydroxy-2-(4-hydroxyphenyl)-4-oxochromen-8-yl]-4,5-dihydroxy-6-(hydroxymethyl)oxan-3-yl] (E)-3-(4-hydroxyphenyl)prop-2-enoate	7.781	C_30_H_26_O_12_	578.1448	3.176	FJGOEBQRHWKKJH-HORBVDEJSA-N
8	(7*S*,8*S*,9*Z*,12*Z*)-7,8-dihydroxyoctadeca-9,12-dienoic acid	10.795	C_18_H_32_O_4_	312.2305	2.66	NMONGVDUESEHOK-MPOZZNMKSA-N
9	9,10,13-trihydroxy-11-octadecenoicacid	7.407	C_18_H_34_O_5_	330.2411	2.317	NTVFQBIHLSPEGQ-BUHFOSPRSA-N
10	(9*Z*)-(13*S*)-12,13-Epoxyoctadeca-9,11-dienoicacid	9.992	C_18_H_30_O_3_	294.2198	2.239	ZFVKKBAQVWQQHP-ALADIWIOSA-N
Acetone extract						
1	(9*Z*)-(13*S*)-12,13-Epoxyoctadeca-9,11-dienoicacid	10.81	C_18_H_30_O_3_	294.2198	14.915	ZFVKKBAQVWQQHP-ALADIWIOSA-N
2	8-Hydroxyluteolin 4′-methyl ether 7-(6′-acetylallosyl) (1->2) (6′-acetylglucoside)	9.171	C_32_H_36_O_19_	724.1829	11.41	ZXQCHXLLIKMUTB-WIFPZZFDSA-N
3	(9*Z*)-(12*S*,13*R*)-12,13-epoxyoctadecenoic acid	10.58	C_18_H_32_O_3_	296.2355	10.974	CCPPLLJZDQAOHD-GJGKEFFFSA-N
4	(9*Z*,15*Z*)-(13*S*)-12,13-Epoxyoctadeca-9,11,15-trienoicacid	10.274	C_18_H_28_O_3_	292.2042	7.2721	YZBZORUZOSCZRN-DCUPSMFCSA-N
5	(7*S*,8*S*,9*Z*,12*Z*)-7,8-dihydroxyoctadeca-9,12-dienoic acid	10.813	C_18_H_32_O_4_	312.2305	4.0297	NMONGVDUESEHOK-MPOZZNMKSA-N
6	[(2*S*,3*R*,4*S*,5*S*,6*R*)-2-[5,7-dihydroxy-2-(4-hydroxyphenyl)-4-oxochromen-8-yl]-4,5-dihydroxy-6-(hydroxymethyl)oxan-3-yl] (E)-3-(4-hydroxyphenyl)prop-2-enoate	7.798	C_30_H_26_O_12_	578.1447	3.1966	FJGOEBQRHWKKJH-HORBVDEJSA-N
7	Hexadecanedioic acid	7.543	C_16_H_30_O_4_	286.2147	3.035	QQHJDPROMQRDLA-UHFFFAOYSA-N
8	(9*Z*)-(13*S*)-12,13-Epoxyoctadeca-9,11-dienoicacid	10.078	C_18_H_30_O_3_	294.2198	2.6986	ZFVKKBAQVWQQHP-ALADIWIOSA-N
9	9,10,13-trihydroxy-11-octadecenoicacid	7.418	C_18_H_34_O_5_	330.241	2.3583	NTVFQBIHLSPEGQ-BUHFOSPRSA-N
Methanol extract						
1	(9*Z*)-(12*S*,13*R*)-12,13-epoxyoctadecenoic acid	10.576	C_18_H_32_O_3_	296.2354	18.507	CCPPLLJZDQAOHD-GJGKEFFFSA-N
2	(9*Z*)-(13*S*)-12,13-Epoxyoctadeca-9,11-dienoicacid	10.805	C_18_H_30_O_3_	294.2198	12.096	ZFVKKBAQVWQQHP-ALADIWIOSA-N
3	9,10,13-trihydroxy-11-octadecenoicacid	7.416	C_18_H_34_O_5_	330.2411	7.071	NTVFQBIHLSPEGQ-BUHFOSPRSA-N
4	(9*Z*,15*Z*)-(13*S*)-12,13-Epoxyoctadeca-9,11,15-trienoicacid	10.274	C_18_H_28_O_3_	292.2042	6.918	YZBZORUZOSCZRN-DCUPSMFCSA-N
5	8-Hydroxyluteolin 4′-methyl ether 7-(6′-acetylallosyl) (1->2) (6′-acetylglucoside)	9.172	C_32_H_36_O_19_	724.1828	5.26	ZXQCHXLLIKMUTB-WIFPZZFDSA-N
6	(9*Z*)-(13*S*)-12,13-Epoxyoctadeca-9,11-dienoicacid	10.003	C_18_H_30_O_3_	294.2198	4.116	ZFVKKBAQVWQQHP-ALADIWIOSA-N
7	Hexadecanedioic acid	7.541	C_16_H_30_O_4_	286.2146	3.969	QQHJDPROMQRDLA-UHFFFAOYSA-N
8	(9*Z*)-(13*S*)-12,13-Epoxyoctadeca-9,11-dienoicacid	10.084	C_18_H_30_O_3_	294.2198	3.547	ZFVKKBAQVWQQHP-ALADIWIOSA-N
9	(7*S*,8*S*,9*Z*,12*Z*)-7,8-dihydroxyoctadeca-9,12-dienoic acid	10.804	C_18_H_32_O_4_	312.2304	3.513	NMONGVDUESEHOK-MPOZZNMKSA-N
Dichloromethane/methanol extract						
1	(9*Z*)-(12*S*,13*R*)-12,13-epoxyoctadecenoic acid	10.583	C_18_H_32_O_3_	296.2354	13.566	CCPPLLJZDQAOHD-GJGKEFFFSA-N
2	(9*Z*)-(13*S*)-12,13-Epoxyoctadeca-9,11-dienoicacid	10.812	C_18_H_30_O_3_	294.2198	10.277	ZFVKKBAQVWQQHP-ALADIWIOSA-N
3	8-Hydroxyluteolin 4′-methyl ether 7-(6′-acetylallosyl) (1->2) (6′-acetylglucoside)	9.18	C_32_H_36_O_19_	724.183	9.5114	ZXQCHXLLIKMUTB-WIFPZZFDSA-N
4	9,10,13-trihydroxy-11-octadecenoicacid	7.424	C_18_H_34_O_5_	330.2411	7.2406	NTVFQBIHLSPEGQ-BUHFOSPRSA-N
5	(9*Z*,15*Z*)-(13*S*)-12,13-Epoxyoctadeca-9,11,15-trienoicacid	10.279	C_18_H_28_O_3_	292.2042	5.3214	YZBZORUZOSCZRN-DCUPSMFCSA-N
6	(7*S*,8*S*,9*Z*,12*Z*)-7,8-dihydroxyoctadeca-9,12-dienoic acid	10.814	C_18_H_32_O_4_	312.2305	3.017	NMONGVDUESEHOK-MPOZZNMKSA-N
7	Hexadecanedioic acid	7.544	C_16_H_30_O_4_	286.2146	2.5871	QQHJDPROMQRDLA-UHFFFAOYSA-N
8	(9*Z*)-(13*S*)-12,13-Epoxyoctadeca-9,11-dienoicacid	10.083	C_18_H_30_O_3_	294.2198	2.5798	ZFVKKBAQVWQQHP-ALADIWIOSA-N
**(b) ESI+**						
**S. No.**	**Compound Name**	**RT (Min)**	**Formula**	**M/Z**	**Peak Area (%) ^1^**	**InChI Key**
Methanol extract						
1	2-Hydroxy-N,N,N-trimethylethan-1-aminium	0.583	C_5_H_13_NO	103.0995	8.911	OEYIOHPDSNJKLS-UHFFFAOYSA-N
2	3-[(3*S*,4*S*,21*R*)-14-Ethyl-21-(methoxycarbonyl)-4,8,13,18-tetramethyl-20-oxo-9-vinyl-3-phorbinyl]propanoic acid	12.36	C_35_H_36_N_4_O_5_	592.2683	7.014	RKEBXTALJSALNU-LDCXZXNSSA-N
3	Hexadecanoic acid	8.206	C_16_H_32_O_2_	273.2665	4.653	IPCSVZSSVZVIGE-UHFFFAOYSA-N
4	(2*R*,3*S*,4*S*,5*S*,6*R*)-2-({7-hydroxy-4-[(3*Z*)-5-hydroxy-3-methylpent-3-en-1-yl]-4a,8,8-trimethyl-3-methylidene-decahydronaphthalen-2-yl}oxy)-6-(hydroxymethyl)oxane-3,4,5-triol	4.895	C_26_H_44_O_8_	506.2886	3.283	
5	(9*Z*)-Hexadec-9-enoic acid	10.037	C_16_H_30_O_2_	276.2089	2.004	SECPZKHBENQXJG-UHFFFAOYSA-N
Acetone extract						
1	3-[(3*S*,4*S*,21*R*)-14-Ethyl-21-(methoxycarbonyl)-4,8,13,18-tetramethyl-20-oxo-9-vinyl-3-phorbinyl]propanoic acid	12.365	C_35_H_36_N_4_O_5_	592.2684	9.229	RKEBXTALJSALNU-LDCXZXNSSA-N
2	methyl (3β,16β,17α,18β,20α)-11,17-dimethoxy-18-[(3,4,5-trimethoxybenzoyl)oxy]yohimban-16-carboxylate	12.152	C_33_H_40_N_2_O_9_	608.2622	5.105	QEVHRUUCFGRFIF-MDEJGZGSSA-N
3	2-Hydroxy-N,N,N-trimethylethan-1-aminium	0.583	C_5_H_13_NO	103.0995	3.663	OEYIOHPDSNJKLS-UHFFFAOYSA-N
4	Hexadecanoic acid	8.206	C_16_H_32_O_2_	273.2668	3.301	IPCSVZSSVZVIGE-UHFFFAOYSA-N
5	3-[(3*S*,4*S*,21*R*)-14-Ethyl-21-(methoxycarbonyl)-4,8,13,18-tetramethyl-20-oxo-9-vinyl-3-phorbinyl]propanoic acid	12.549	C_35_H_36_N_4_O_5_	592.2684	2.713	RKEBXTALJSALNU-LDCXZXNSSA-N
6	(2*R*,3*S*,4*S*,5*S*,6*R*)-2-({7-hydroxy-4-[(3*Z*)-5-hydroxy-3-methylpent-3-en-1-yl]-4a,8,8-trimethyl-3-methylidene-decahydronaphthalen-2-yl}oxy)-6-(hydroxymethyl)oxane-3,4,5-triol	4.887	C_26_H_44_O_8_	506.2885	2.119	
Ethyl acetate extract						
1	3-[(3*S*,4*S*,21*R*)-14-Ethyl-21-(methoxycarbonyl)-4,8,13,18-tetramethyl-20-oxo-9-vinyl-3-phorbinyl]propanoic acid	12.367	C_35_H_36_N_4_O_5_	592.2681	12.56	RKEBXTALJSALNU-LDCXZXNSSA-N
2	methyl (3β,16β,17α,18β,20α)-11,17-dimethoxy-18-[(3,4,5-trimethoxybenzoyl)oxy]yohimban-16-carboxylate	12.152	C_33_H_40_N_2_O_9_	608.2624	5.663	QEVHRUUCFGRFIF-MDEJGZGSSA-N
3	Hexadecanoic acid	8.208	C_16_H_32_O_2_	273.2667	5.587	IPCSVZSSVZVIGE-UHFFFAOYSA-N
4	Bis(methylbenzylidene)sorbitol	7.809	C_22_H_26_O_6_	386.1725	3.821	WOOQSKAMMPIQIW-HCXPZJNHSA-N
5	Unknown	12.549	C_35_H_36_N_4_O_5_	592.2681	3.169	OINDWIFDMFYGDX-LDCXZXNSSA-N
Ethanol extract						
1	Unknown	12.363	C_38_H_35_F_3_N_2_O	592.2682	8.414	
2	2-Hydroxy-N,N,N-trimethylethan-1-aminium	0.584	C_5_H_13_NO	103.0995	6.534	OEYIOHPDSNJKLS-UHFFFAOYSA-N
3	(2*R*,3*S*,4*S*,5*S*,6*R*)-2-({7-hydroxy-4-[(3*Z*)-5-hydroxy-3-methylpent-3-en-1-yl]-4a,8,8-trimethyl-3-methylidene-decahydronaphthalen-2-yl}oxy)-6-(hydroxymethyl)oxane-3,4,5-triol	4.898	C_26_H_44_O_8_	506.2887	4.339	
4	Hexadecanoic acid	8.21	C_16_H_32_O_2_	273.2668	3.981	IPCSVZSSVZVIGE-UHFFFAOYSA-N
5	methyl (3β,16β,17α,18β,20α)-11,17-dimethoxy-18-[(3,4,5-trimethoxybenzoyl)oxy]yohimban-16-carboxylate	12.152	C_33_H_40_N_2_O_9_	608.2623	3.057	QEVHRUUCFGRFIF-MDEJGZGSSA-N
6	3-[(3*S*,4*S*,21*R*)-14-Ethyl-21-(methoxycarbonyl)-4,8,13,18-tetramethyl-20-oxo-9-vinyl-3-phorbinyl]propanoic acid	12.55	C_35_H_36_N_4_O_5_	592.2682	2.644	RKEBXTALJSALNU-LDCXZXNSSA-N
Dichloromethane/methanol extract						
1	Unknown	13.965	C_23_H_30_ClN_7_O_2_S_2_	535.1584	23.76	
2	(Z)-docos-13-enamide	12.806	C_22_H_43_NO	337.3346	4.218	UAUDZVJPLUQNMU-KTKRTIGZSA-N
3	2-[[(5*S*,6*R*,9*S*)-14-[[(2-fluoroanilino)-oxomethyl]amino]-5-methoxy-3,6,9-trimethyl-2-oxo-11-oxa-3,8-diazabicyclo[10.4.0]hexadeca-1(12),13,15-trien-8-yl]methyl]benzoic acid	11.623	C_32_H_37_FN_4_O_6_	592.2678	2.383	
4	Unknown	13.966	C_21_H_35_ClFN_7_S_3_	535.1805	2.087	

^1^ Peak area (%) is based on the total sum.

**Table 2:** Major derivatized constitutes of different solvent *E. foeminea* extracts analyzed by GC–MS.

S. No.	Compound Name	RT (Min)	Formula	Peak Area (%) ^1^	Inchi Key
Methanol extract					
1	Propane-1,2,3-triol (3TMS)	15.69	C_12_H_32_O_3_Si_3_	3.5288	PEDCQBHIVMGVHV-UHFFFAOYSA-N
2	Hexadecanoic acid (TMS)	30.66	C_19_H_40_O_2_Si	2.9008	IPCSVZSSVZVIGE-UHFFFAOYSA-N
3	(3*S*,4*R*,5*R*)-1,3,4,5,6-Pentahydroxyhexan-2-one (methyloxime 5TMS)	27.72	C_22_H_55_NO_6_S_i5_	2.7562	RFSUNEUAIZKAJO-VRPWFDPXSA-N
4	(3*S*,4*S*,5*R*)-1,3,4,5,6-Pentahydroxy-hexan-2-one	27.90	C_6_H_12_O_6_	2.1988	RFSUNEUAIZKAJO-VRPWFDPXSA-N
5	4-Hydroxyquinoline-2-carboxylic acid (2TMS)	30.97	C_16_H_23_NO_3_Si_2_	2.1261	HCZHHEIFKROPDY-UHFFFAOYSA-N
6	(3*S*,4*S*,5*S*,6*R*)-6-(hydroxymethyl)oxane-2,3,4,5-tetrol (methyloxime 5TMS)	28.17	C_22_H_55_NO_6_Si_5_	2.0817	WQZGKKKJIJFFOK-UHFFFAOYNA-N
Dichloromethane/methanol extract					
1	Octadecanoic acid (TMS)	33.55	C_2_^1^_H44_O_2_Si	3.7416	QIQXTHQIDYTFRH-UHFFFAOYSA-N
2	Propane-1,2,3-triol (3TMS)	15.33	C_12_H_32_O_3_Si3	3.5086	PEDCQBHIVMGVHV-UHFFFAOYSA-N
3	Hexadecanoic acid (TMS)	30.58	C_19_H_40_O_2_Si	3.5085	IPCSVZSSVZVIGE-UHFFFAOYSA-N
4	Tetradecanoic acid	33.56	C_14_H_28_O_2_	2.4111	TUNFSRHWOTWDNC-UHFFFAOYSA-N
5	1,3-Diazinane-2,4-dione (2TMS)	12.26	C_10_H_22_N_2_O_2_Si_2_	2.098	OIVLITBTBDPEFK-UHFFFAOYSA-N
Ethanol extract					
1	Propane-1,2,3-triol (3TMS)	15.69	C_12_H_32_O_3_Si_3_	3.705	PEDCQBHIVMGVHV-UHFFFAOYSA-N
2	Hexadecanoic acid (TMS)	30.66	C_19_H_40_O_2_Si	3.3054	IPCSVZSSVZVIGE-UHFFFAOYSA-N
3	Octadecanoic acid (TMS)	33.64	C_21_H_44_O_2_Si	2.649	QIQXTHQIDYTFRH-UHFFFAOYSA-N
4	(2*S*,3*R*,4*S*,5*R*,6*R*)-6-(hydroxymethyl)oxane-2,3,4,5-tetrol	28.85	C_6_H_12_O_6_	2.4626	WQZGKKKJIJFFOK-UHFFFAOYNA-N
Acetone extract					
1	Hexadecanoic acid (TMS)	30.66	C_19_H_40_O_2_Si	4.107	IPCSVZSSVZVIGE-UHFFFAOYSA-N
2	Octadecanoic acid (TMS)	33.644	C_2_^1^_H44_O_2_Si	2.7569	QIQXTHQIDYTFRH-UHFFFAOYSA-N
3	Propane-1,2,3-triol (3TMS)	15.69	C_12_H_32_O_3_Si_3_	2.3189	PEDCQBHIVMGVHV-UHFFFAOYSA-N
4	1,3-Diazinane-2,4-dione (2TMS)	12.30	C_10_H_22_N_2_O_2_Si_2_	1.9999	OIVLITBTBDPEFK-UHFFFAOYSA-N
Ethyl acetate extract					
1	1,3-Diazinane-2,4-dione (2TMS)	12.33	C_10_H_22_N_2_O_2_Si_2_	7.8011	OIVLITBTBDPEFK-UHFFFAOYSA-N
2	Hexadecanoic acid (TMS)	30.66	C_19_H_40_O_2_Si	3.6583	IPCSVZSSVZVIGE-UHFFFAOYSA-N

^1^ Peak area (%) is based on the total sum.

**Table 3:** Qualitative screening of the top compound classes present in the different solvent crude extracts of *E. foeminea* analyzed by GC–MS.

Class Group	Ace	DCM/MeOH	EA	EtOH	MeOH	ChemOnt ID
1,2-aminoalcohols	−	+	−	+	+	0001897
1,2-diols	−	−	−	+	−	0002467
Acylaminosugars	−	−	−	−	+	0000146
Alkali metal pyrophosphates	−	−	−	−	+	0000657
Amino acids	−	+	−	+	+	0004176
Amino Fatty Acids	−	+	−	−	+	0000489
Benzoic acids	+	+	+	+	+	0002565
Beta hydroxy acids	−	−	−	+	+	0001713
Branched Fatty Acids	−	−	−	−	+	0000338
Butenolides	−	+	−	−	+	0002223
Cyclitols	+	+	−	+	+	0004344
Dicarboxylic acids	+	+	+	+	+	0000346
Disaccharides	−	+	−	−	+	0001542
Fatty acids and conjugates	+	+	+	+	+	0000262
Hippuric acids	+	+	−	+	+	0001318
Hydroxy Fatty Acids	+	+	+	+	+	0000341
Hydroxybenzaldehydes	−	−	−	−	+	0003978
Hydroxybenzoic acids	−	+	−	+	+	0001248
Monoalkylamines	−	−	−	+	+	0000469
Monosaccharides	+	+	−	+	+	0001540
o-Xylenes	+	−	−	−	−	0004210
Organic acids	+	+	+	+	+	0000264
Pyridines	+	+	+	+	+	0000089
Pyrimidine ribonucleoside diphosphates	−	−	−	−	+	0001621
Pyrimidines	+	+	+	+	+	0000075
Pyrroline carboxylic acids	+	+	−	+	+	0002417
Quinoline carboxylic acids	−	−	−	−	+	0002552
Salicylic acids	−	−	−	−	+	0002514
Sugar acids	−	+	−	−	+	0000215
Sugar alcohols	+	+	+	+	+	0002210
Ureas	−	−	−	+	+	0000517

+ = present, − = absent. Ace: acetone; DCM/MeOH: dichloromethane/methanol; EA: ethyl acetate; EtOH: ethanol; MeOH: methanol.

## Data Availability

Data is contained within the article or Supplementary Material.

## Supplementary Materials

The following supporting information can be downloaded at: https://www.mdpi.com/article/10.3390/metabo12050451/s1, Figure S1: (a) ^1^H NMR 1D spectrum of *E. foeminea* acetone extract recorded using 600 MHz liquid-state NMR and dissolved in D_2_O; (b) extended region of CH_3_ and CH_2_ mainly CH_3_ of amino acids (δ 3–0); (c) polysaccharides extended region (δ 4.5–3); (d) aromatic region.; Figure S2: (a) ^1^H NMR 1D spectrum of *E. foeminea* dichloromethane/methanol extract recorded using 600 MHz liquid-state NMR and dissolved in D_2_O; (b) extended region of CH_3_ and CH_2_ mainly CH_3_ of amino acids (δ 3–0); (c) polysaccharides extended region (δ 4.5–3); (d) aromatic region; Figure S3: (a) ^1^H NMR 1D spectrum of *E. foeminea* ethanol extract recorded using 600 MHz liquid-state NMR and dissolved in D_2_O; (b) extended region of CH_3_ and CH_2_ mainly CH_3_ of amino acids (δ 3–0); (c) polysaccharides extended region (δ 4.5–3); (d) aromatic region; Figure S4: (a) ^1^H NMR 1D spectrum of *E. foeminea* ethyl acetate extract recorded using 600 MHz liquid-state NMR and dissolved in D_2_O; (b) extended region of CH_3_ and CH_2_ mainly CH_3_ of amino acids (δ 3–0); (c) polysaccharides extended region (δ 4.5–3); (d) aromatic region; Figure S5: (a) ^1^H NMR 1D spectrum of *E. foeminea* methanol extract recorded using 600 MHz liquid-state NMR and dissolved in D_2_O; (b) extended region of CH_3_ and CH_2_ mainly CH_3_ of amino acids (δ 3–0); (c) polysaccharides extended region (δ 4.5–3); (d) aromatic region; Figure S6: (a) ^1^H NMR 1D spectrum of *E. foeminea* acetone extract recorded using 500 MHz liquid-state NMR and dissolved in CDCl3; (b) extended region of CH_3_ and CH_2_ mainly CH_3_ of amino acids (δ 3–0); (c) polysaccharides extended region (δ 4.5–3); (d) aromatic region; Figure S7: (a) ^1^H NMR 1D spectrum of *E. foeminea* dichloromethane/methanol extract recorded using 500 MHz liquid-state NMR and dissolved in CDCl3; (b) extended region of CH_3_ and CH_2_ mainly CH_3_ of amino acids (δ 3–0); (c) polysaccharides extended region (δ 4.5–3); (d) aromatic region; Figure S8: (a) ^1^H NMR 1D spectrum of *E. foeminea* ethanol extract recorded using 500 MHz liquid-state NMR and dissolved in CDCl3; (b) extended region of CH_3_ and CH_2_ mainly CH_3_ of amino acids (δ 3–0); (c) polysaccharides extended region (δ 4.5–3); (d) aromatic region; Figure S9: (a) ^1^H NMR 1D spectrum of *E. foeminea* ethanol extract recorded using 500 MHz liquid-state NMR and dissolved in CDCl3; (b) extended region of CH_3_ and CH_2_ mainly CH_3_ of amino acids (δ 3–0); (c) polysaccharides extended region (δ 4.5–3); (d) aromatic region; Figure S10: (a) ^1^H NMR 1D spectrum of *E. foeminea* methanol extract recorded using 500 MHz liquid-state NMR and dissolved in CDCl3; (b) extended region of CH_3_ and CH_2_ mainly CH_3_ of amino acids (δ 3–0); (c) polysaccharides extended region (δ 4.5–3); (d) aromatic region; Figure S11: Typical positive mode LC–MS total ion chromatograms (TIC) of different *E. foeminea* crude extracts. (a) acetone; (b) dichloromethane/methanol; (c) ethanol; (d) ethyl acetate; (e) methanol; Figure S12: Typical negative mode LC–MS total ion chromatograms (TIC) of different *E. foeminea* crude extracts. a) acetone; (b) dichloromethane/methanol; (c) ethanol; (d) ethyl acetate; (e) methanol; Figure S13: GC–MS total ion chromatograms (TIC) of derivatized *E. foeminea* crude extracts using different solvents. (a) acetone; (b) dichloromethane/methanol; (c) ethanol; (d) ethyl acetate; (e) methanol.; Figure S14: (a) Over Representation Analysis (ORA) Bar chart and (b) pie chart of the chemical classification of the total metabolites identified in the *E. foeminea* acetone crude extract sample using GC–MS via metabolite set enrichment analysis (MSEA); Figure S15: (a) Over Representation Analysis (ORA) Bar chart and (b) pie chart of the chemical classification of the total metabolites identified in the *E. foeminea* dichloromethane/methanol crude extract sample using GC–MS via metabolite set enrichment analysis (MSEA); Figure S16: (a) Over Representation Analysis (ORA) Bar chart and (b) pie chart of the chemical classification of the total metabolites identified in the *E. foeminea* ethanol crude extract sample using GC–MS via metabolite set enrichment analysis (MSEA); Figure S17: (a) Over Representation Analysis (ORA) Bar chart and (b) pie chart of the chemical classification of the total metabolites identified in the *E. foeminea* ethyl acetate crude extract sample using GC–MS via metabolite set enrichment analysis (MSEA); Figure S18: (a) Over Representation Analysis (ORA) Bar chart and (b) pie chart of the chemical classification of the total metabolites identified in the *E. foeminea* methanol crude extract sample using GC–MS via metabolite set enrichment analysis (MSEA); Figure S19: Screenshot of Chenomx Profiler (Chenomx Suite 9.0, Alberta, Canada) software window. As shown in the figure, the peaks at δ 1.05 ppm and 0.99 ppm can be assigned to valine; Figure S20: Screenshot of Chenomx Profiler (Chenomx Suite 9.0, Alberta, Canada) software window. As shown in the figure, the doublet at δ 0.96 ppm and 0.97 ppm can be assigned to leucine; Figure S21: Screenshot of Chenomx Profiler (Chenomx Suite 9.0, Alberta, Canada) software window. As shown in the figure, the peaks at δ 1.1 ppm and 0.9 ppm can be assigned to isoleucine; Figure S22: Screenshot of Chenomx Profiler (Chenomx Suite 9.0, Alberta, Canada) software window. As shown in the figure, the peaks mainly found between δ 3.2 ppm and 3.9 ppm can be assigned to glucose; Figure S23: Screenshot of Chenomx Profiler (Chenomx Suite 9.0, Alberta, Canada) software window. As shown in the figure, the peaks mainly found between δ 3.4 ppm and 4.0 ppm can be assigned to maltose; Figure S24: Screenshot of Chenomx Profiler (Chenomx Suite 9.0, Alberta, Canada) software window. As shown in the figure, the peaks mainly found between δ 3.4 ppm and 4.9 ppm can be assigned to mannose; Figure S25: Screenshot of Chenomx Profiler (Chenomx Suite 9.0, Alberta, Canada) software window. As shown in the figure, the peaks mainly found between δ 3.5 ppm and 4.9 ppm can be assigned to ribose; Figure S26: Screenshot of Chenomx Profiler (Chenomx Suite 9.0, Alberta, Canada) software window. As shown in the figure, the peaks mainly found between δ 3.5 ppm and 4.2 ppm can be assigned to sucrose; Figure S27: ^1^H NMR 1D spectrum of *E. foeminea* methanolic extract recorded using 600 MHz liquid-NMR displaying the solvent region acquired with water suppression pulse program; Figure S28: ^1^H NMR 1D spectrum of *E. foeminea* methanolic extract recorded using 600 MHz liquid-NMR displaying the internal reference DSS region at δ 0.0 ppm; Figure S29: (a) ^1^H NMR 1D spectrum of *E. foeminea* methanolic extract recorded using 600 MHz liquid-NMR with water suppression (red) and without water suppression (blue); (b) extended region; Table S1: Identified metabolites in *E. foeminea* crude extracts analyzed by positive mode LC–MS. (a) acetone extract; (b) dichloromethane/methanol extract; (c) ethanol extract; (d) ethyl acetate extract; (e) methanol extract.; Table S2: Common and unique compounds identified in different *E. foeminea* crude extracts via Venn diagram analysis using positive ion mode LC–MS; Table S3: Identified metabolites in *E. foeminea* crude extracts analyzed by negative mode LC–MS. (a) acetone extract; (b) dichloromethane/methanol extract; (c) ethanol extract; (d) ethyl acetate extract; (e) methanol extract; Table S4: Common and unique compounds identified in different *E. foeminea* crude extracts via Venn diagram analysis using negative ion mode LC–MS; Table S5: Identified metabolites in *E. foeminea* crude extracts analyzed by GC–MS. (a) acetone extract; (b) dichloromethane/methanol extract; (c) ethanol extract; (d) ethyl acetate extract; (e) methanol extract.; Table S6: Common and unique compounds identified in different *E. foeminea* crude extracts via Venn diagram analysis using GC–MS; Table S7: Result from Over Representation Analysis (ORA) of all five *E. foeminea* crude extract samples; Table S8: Result from Over Representation Analysis (ORA) of different *E. foeminea* crude extract samples. (a) acetone extract; (b) dichloromethane/methanol extract; (c) ethanol extract; (d) ethyl acetate extract; (e) methanol extract; Table S9: Raw spectral data of *E. foeminea* acetone crude extract analyzed using GC–MS; Table S10: Raw spectral data of *E. foeminea* dichloromethane/methanol crude extract analyzed using GC–MS Table S11: Raw spectral data of *E. foeminea* ethanol crude extract analyzed using GC–MS; Table S12: Raw spectral data of *E. foeminea* ethyl acetate crude extract analyzed using GC–MS; Table S13: Raw spectral data of *E. foeminea* methanol crude extract analyzed using GC–MS; Table S14: Raw spectral data of *E. foeminea* acetone crude extract analyzed using ESI+ LC–MS; Table S15: Raw spectral data of *E. foeminea* dichloromethane/methanol crude extract analyzed using ESI+ LC–MS; Table S16: Raw spectral data of *E. foeminea* ethanol crude extract analyzed using ESI+ LC–MS; Table S17: Raw spectral data of *E. foeminea* ethyl acetate crude extract analyzed using ESI+ LC–MS; Table S18: Raw spectral data of *E. foeminea* methanol crude extract analyzed using ESI+ LC–MS; Table S19: Raw spectral data of *E. foeminea* acetone crude extract analyzed using ESI− LC–MS; Table S20: Raw spectral data of *E. foeminea* dichloromethane/methanol crude extract analyzed using ESI− LC–MS; Table S21: Raw spectral data of *E. foeminea* ethanol crude extract analyzed using ESI− LC–MS; Table S22: Raw spectral data of *E. foeminea* ethyl acetate crude extract analyzed using ESI− LC–MS; Table S23: Raw spectral data of *E. foeminea* methanol crude extract analyzed using ESI− LC–MS.
