# Supplementary material for: Untargeted Metabolomic Profiling and Antioxidant Capacities of Different Solvent Crude Extracts of Ephedra foeminea
Source: Metabolites. 2022 May 17;12(5):451. doi: 10.3390/metabo12050451 (PMC9146585; doi:10.3390/metabo12050451)
Supplement: Supplementary file 1 [file metabolites-12-00451-s001.zip › Supplementary Figures.pdf]

Supplementary Figures

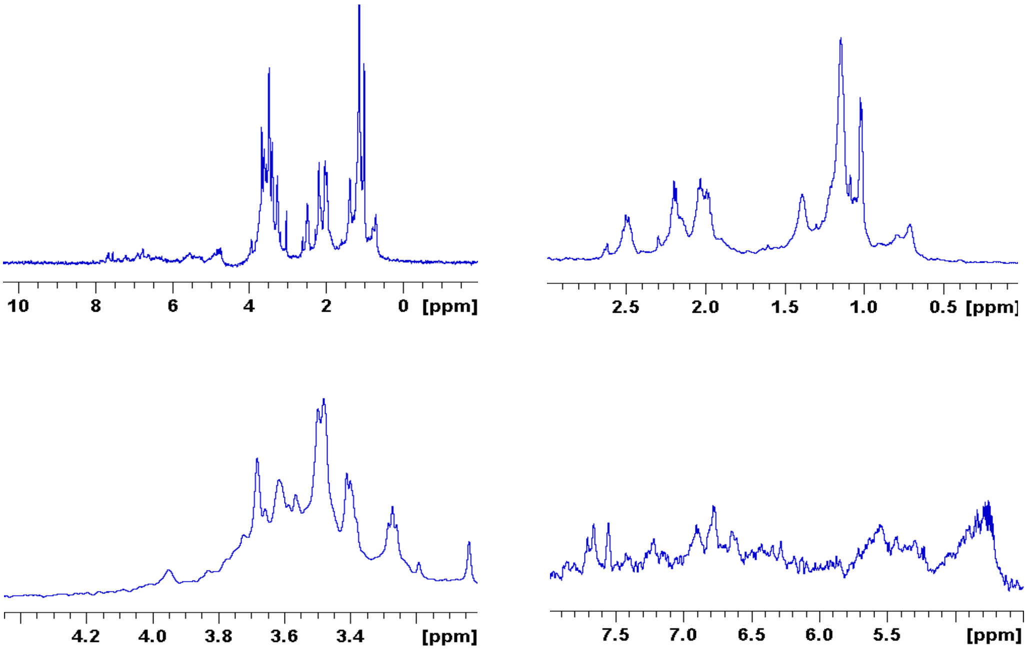

Figure S1. (a) ^{1}H NMR 1D spectrum of *E. foeminea* acetone extract recorded using 600 MHz liquid-NMR and dissolved in D2O; **(b)** extended region of CH_3 and CH_2 mainly CH_3 of amino acids (δ 3–0); **(c)** polysaccharides extended region (δ 4.5–3); **(d)** aromatic region.

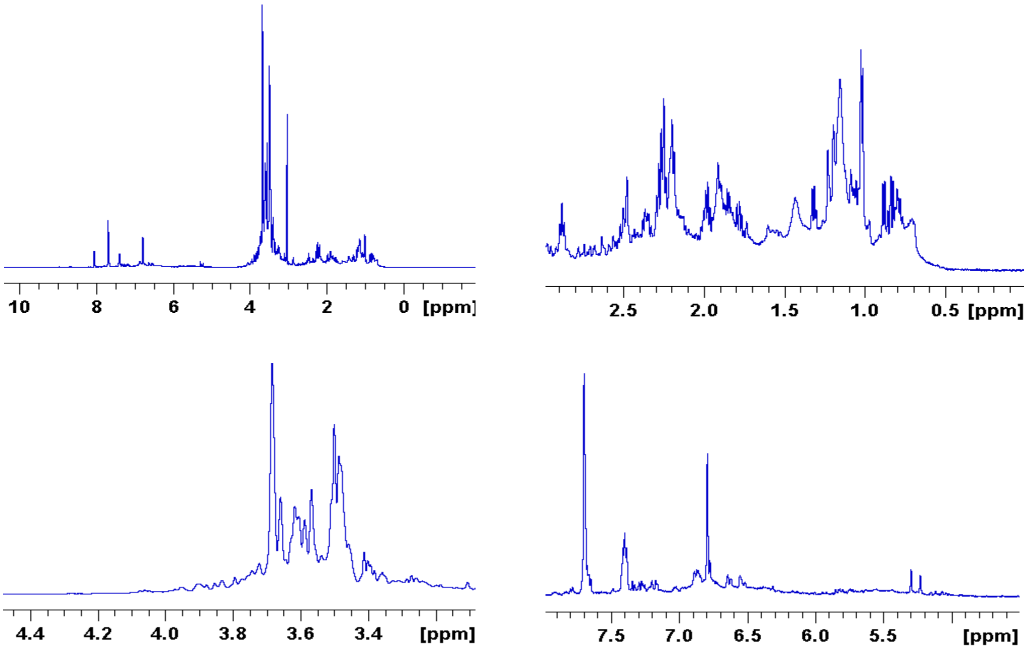

Figure S2. (a) ^{1}H NMR 1D spectrum of *E. foeminea* dichloromethane/methanol extract recorded using 600 MHz liquid-NMR and dissolved in D2O; **(b)** extended region of CH_3 and CH_2 mainly CH_3 of amino acids (δ 3–0); **(c)** polysaccharides extended region (δ 4.5–3); **(d)** aromatic region.

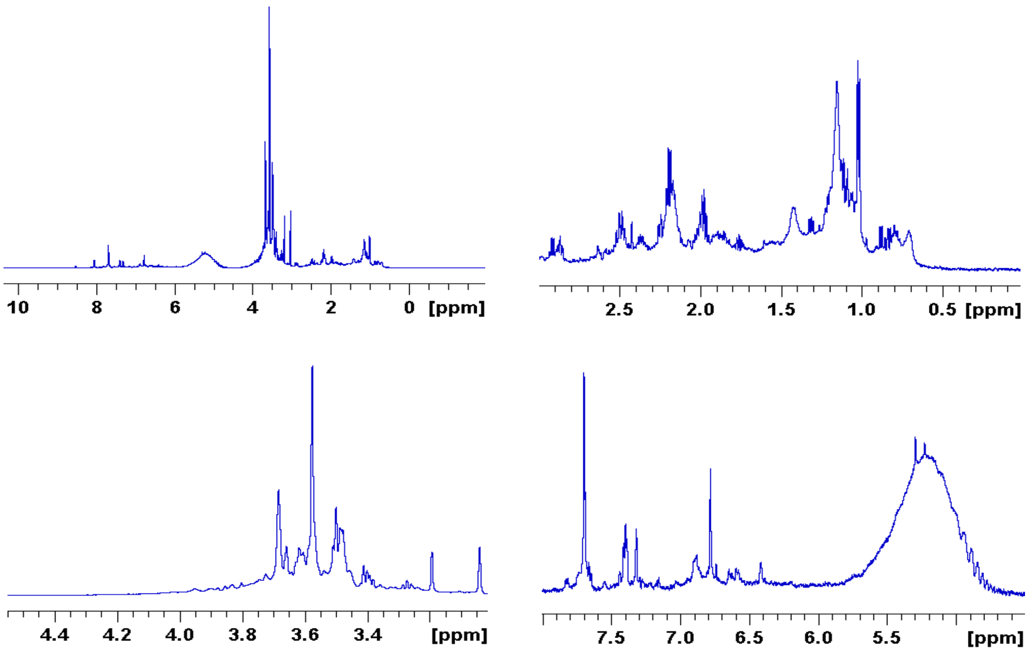

Figure S3. (a) ^{1}H NMR 1D spectrum of *E. foeminea* ethanol extract recorded using 600 MHz liquid-NMR and dissolved in D2O; **(b)** extended region of CH_3 and CH_2 mainly CH_3 of amino acids (δ 3–0); **(c)** polysaccharides extended region (δ 4.5–3); **(d)** aromatic region.

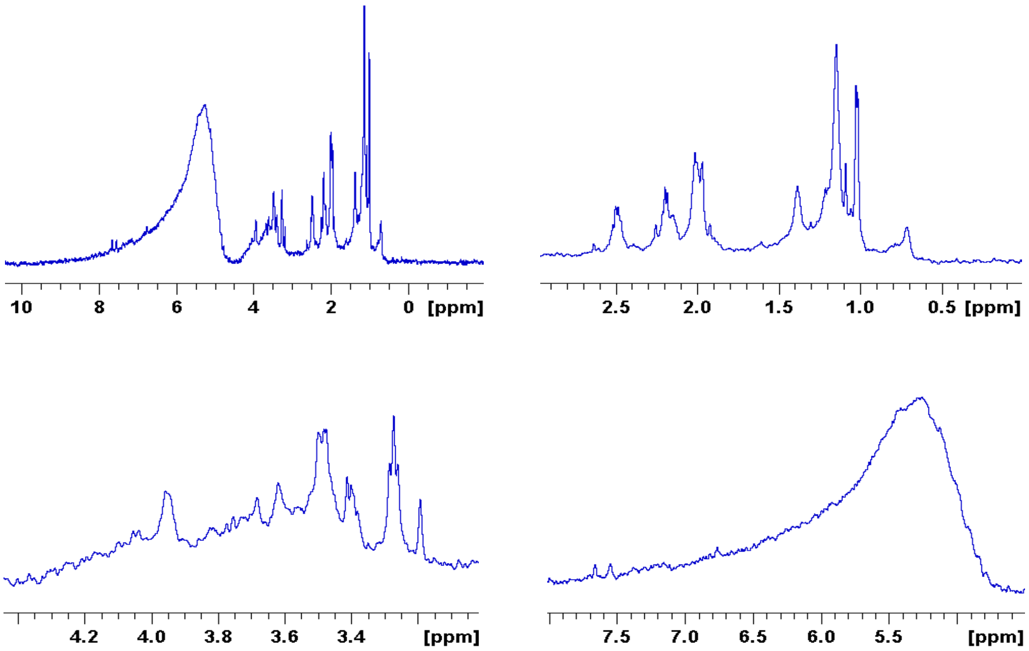

Figure S4. (a) ^{1}H NMR 1D spectrum of *E. foeminea* ethyl acetate extract recorded using 600 MHz liquid-NMR and dissolved in D2O; **(b)** extended region of CH_3 and CH_2 mainly CH_3 of amino acids (δ 3–0); **(c)** polysaccharides extended region (δ 4.5–3); **(d)** aromatic region.

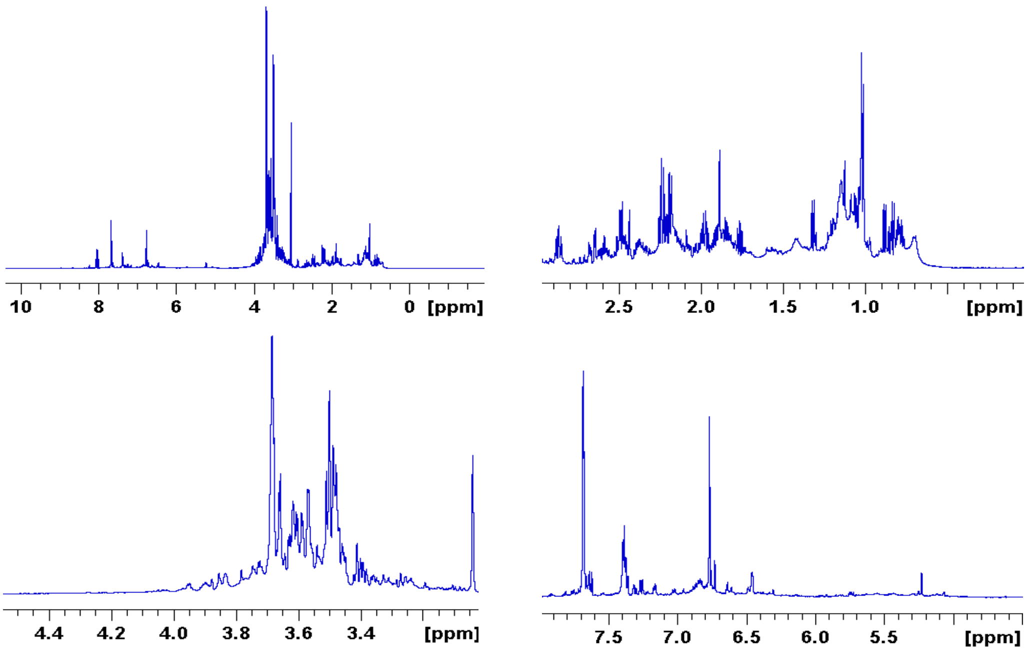

Figure S5. **(a)** ^{1}H NMR 1D spectrum of *E. foeminea* methanol extract recorded using 600 MHz liquid-NMR and dissolved in D2O; **(b)** extended region of CH_3 and CH_2 mainly CH_3 of amino acids (δ 3–0); **(c)** polysaccharides extended region (δ 4.5–3); **(d)** aromatic region.

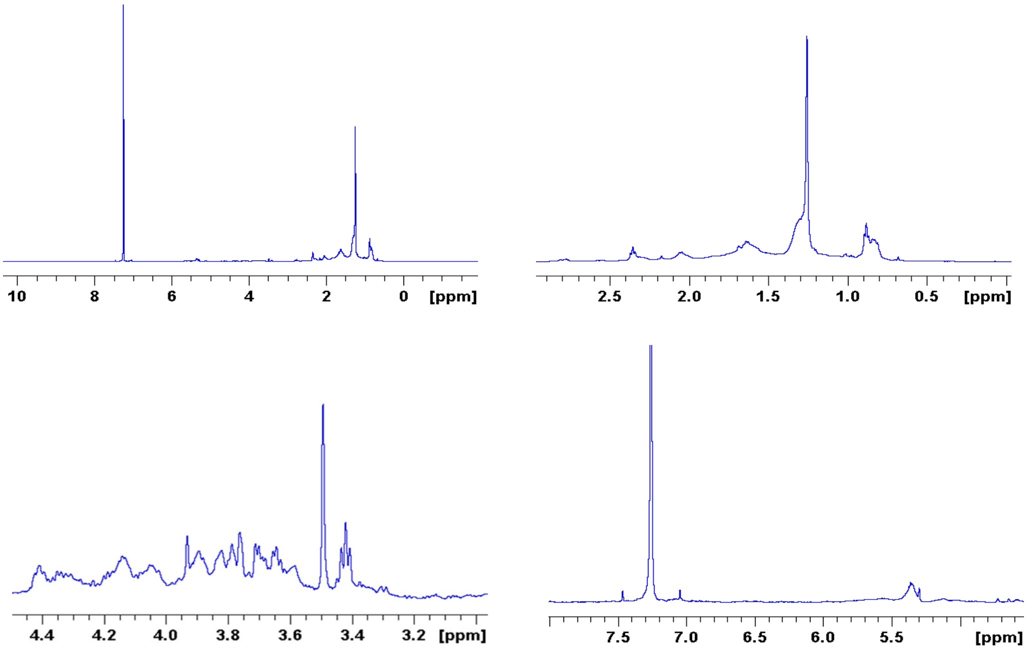

Figure S6. **(a)** ^{1}H NMR 1D spectrum of *E. foeminea* acetone extract recorded using 500 MHz liquid-NMR and dissolved in CDCL3; **(b)** extended region of CH_3 and CH_2 mainly CH_3 of amino acids (δ 3–0); **(c)** polysaccharides extended region (δ 4.5–3); **(d)** aromatic region.

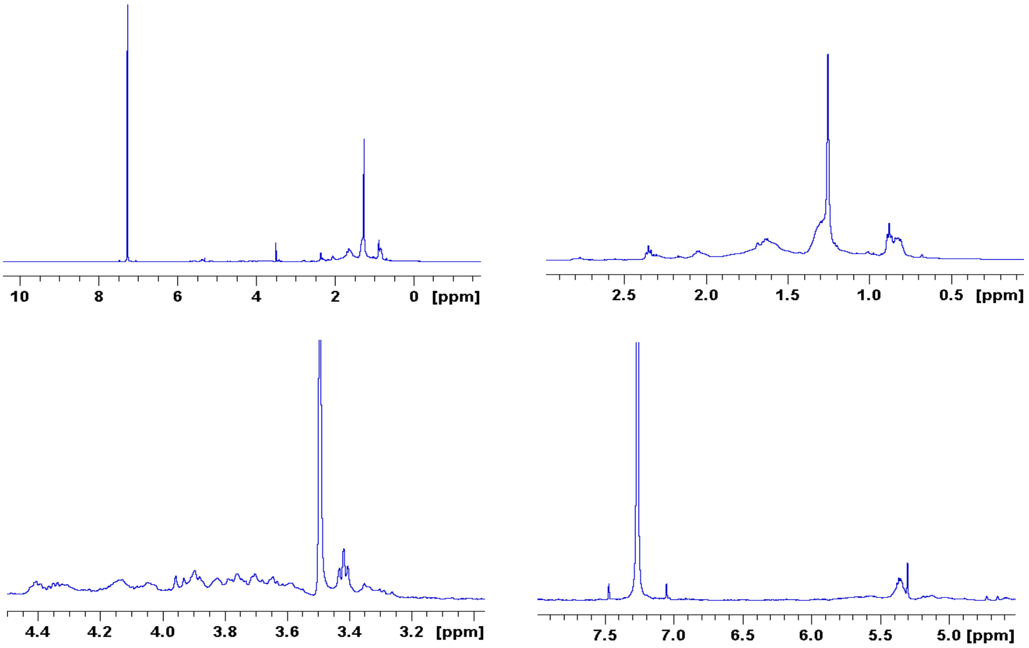

Figure S7. (a) ^{1}H NMR 1D spectrum of *E. foeminea* dichloromethane/methanol extract recorded using 500 MHz liquid-NMR and dissolved in CDCL3; **(b)** extended region of CH_3 and CH_2 mainly CH_3 of amino acids (δ 3–0); **(c)** polysaccharides extended region (δ 4.5–3); **(d)** aromatic region.

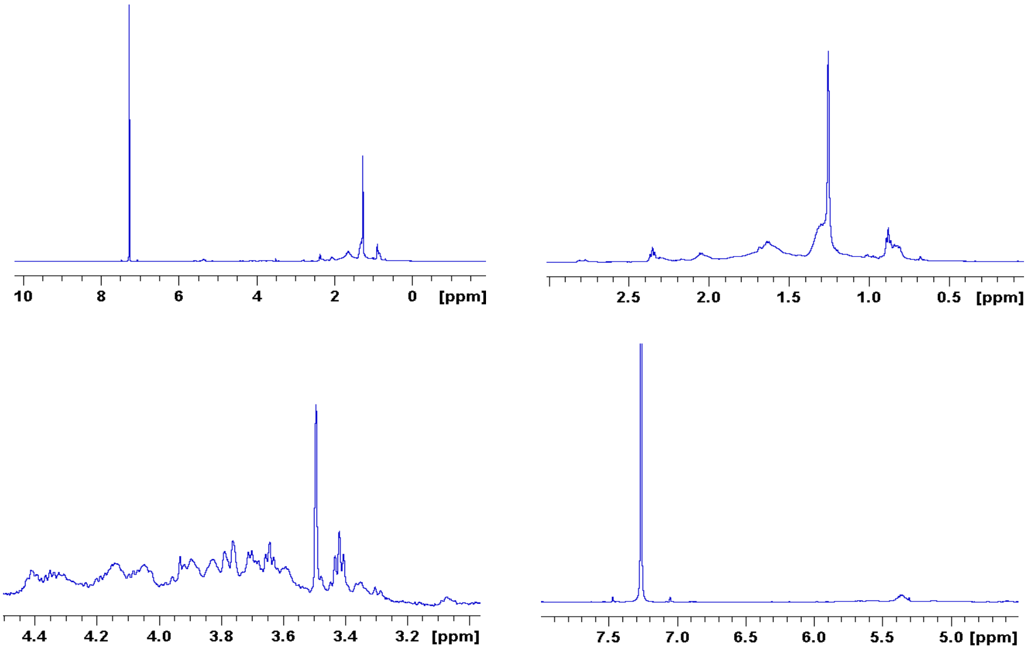

Figure S8. (a) ^{1}H NMR 1D spectrum of *E. foeminea* ethanol extract recorded using 500 MHz liquid-NMR and dissolved in CDCL3; **(b)** extended region of CH_3 and CH_2 mainly CH_3 of amino acids (δ 3–0); **(c)** polysaccharides extended region (δ 4.5–3); **(d)** aromatic region.

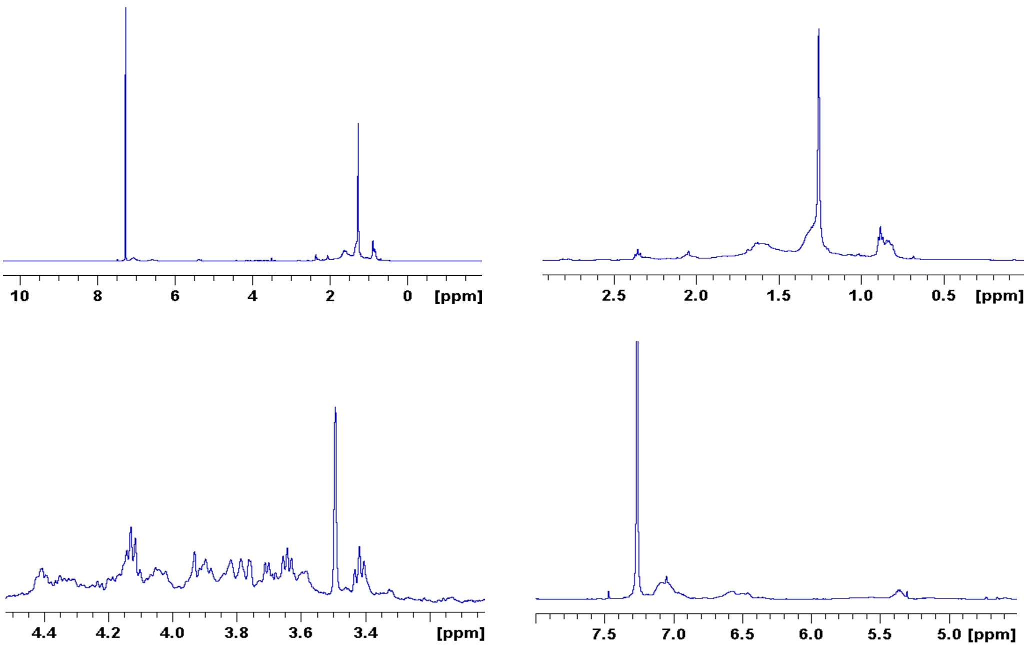

Figure S9. (a) ^{1}H NMR 1D spectrum of *E. foeminea* ethyl acetate extract recorded using 500 MHz liquid-NMR and dissolved in CDCL3; **(b)** extended region of CH_3 and CH_2 mainly CH_3 of amino acids (δ 3–0); **(c)** polysaccharides extended region (δ 4.5–3); **(d)** aromatic region.

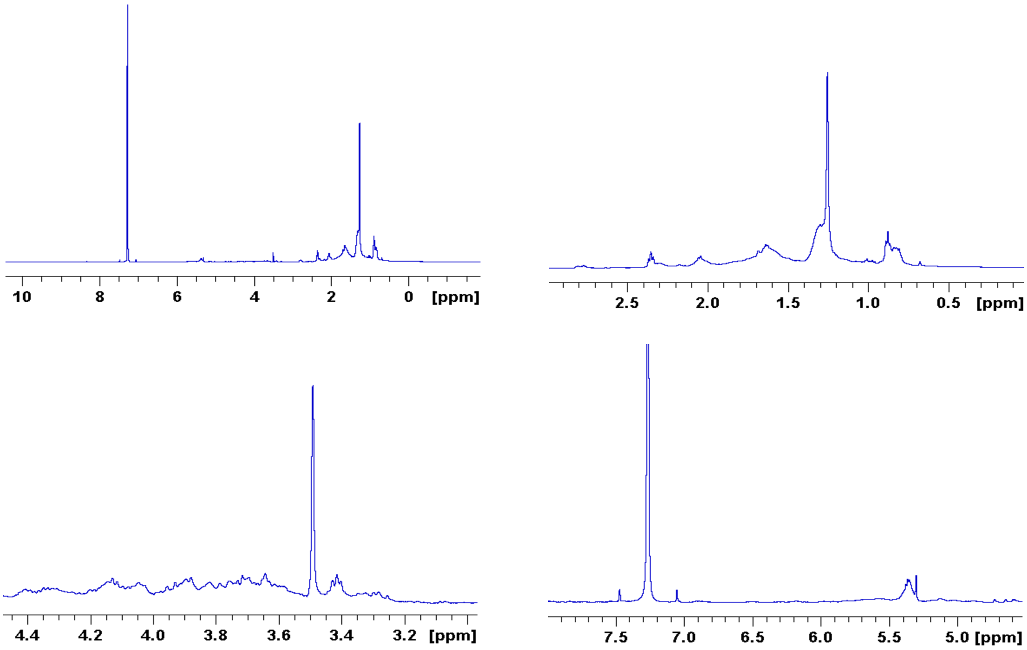

Figure S10. (a) ^{1}H NMR 1D spectrum of *E. foeminea* methanol extract recorded using 500 MHz liquid-NMR and dissolved in CDCL3; **(b)** extended region of CH_3 and CH_2 mainly CH_3 of amino acids (δ 3–0); **(c)** polysaccharides extended region (δ 4.5–3); **(d)** aromatic region.

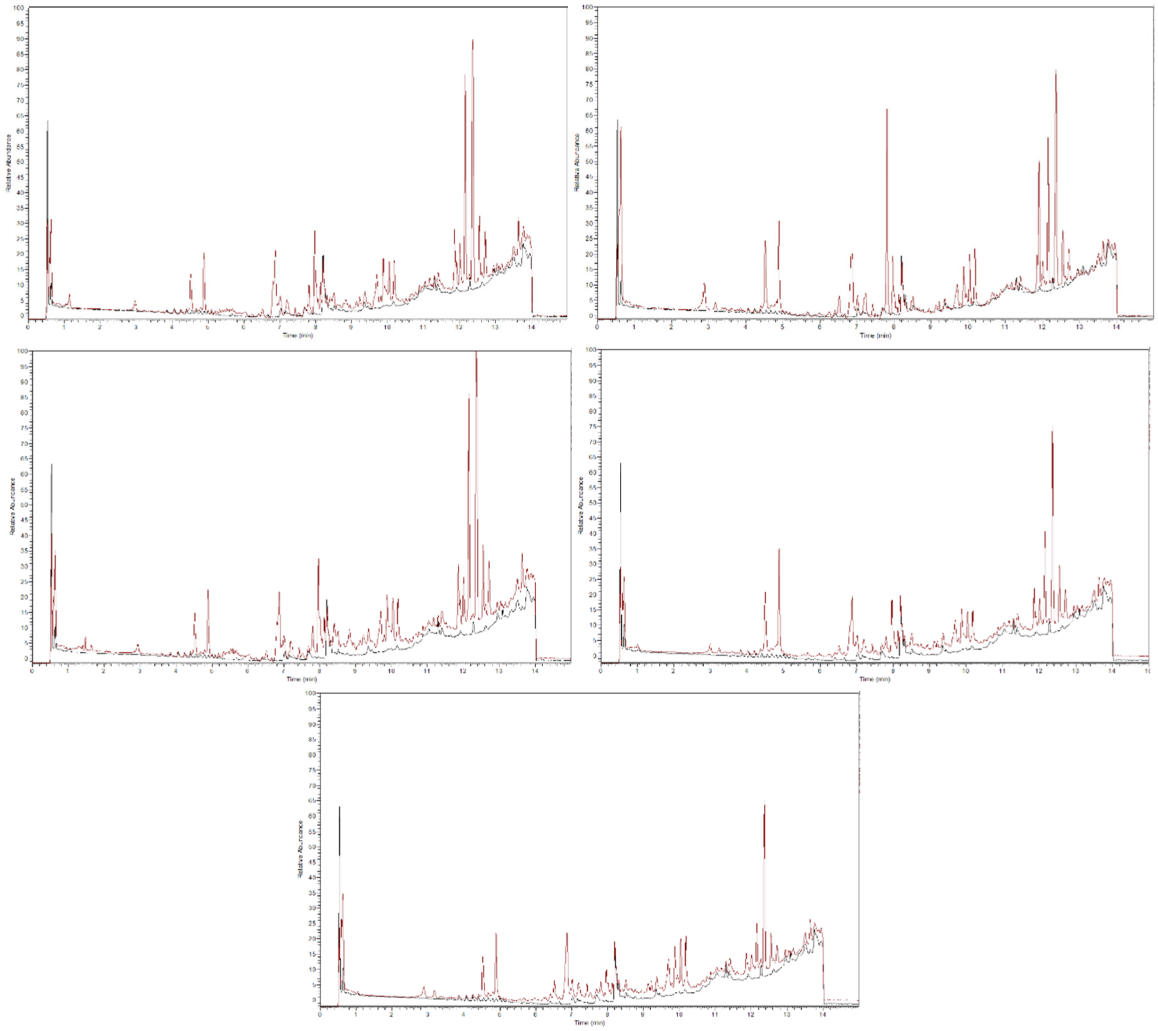

Figure S11. Typical positive mode LC-MS total ion chromatograms (TIC) of different *E. foeminea* crude extracts; **(a)** acetone; **(b)** dichloromethane/methanol; **(c)** ethanol; **(d)** ethyl acetate; **(e)** methanol. All chromatograms are displayed against the blank.

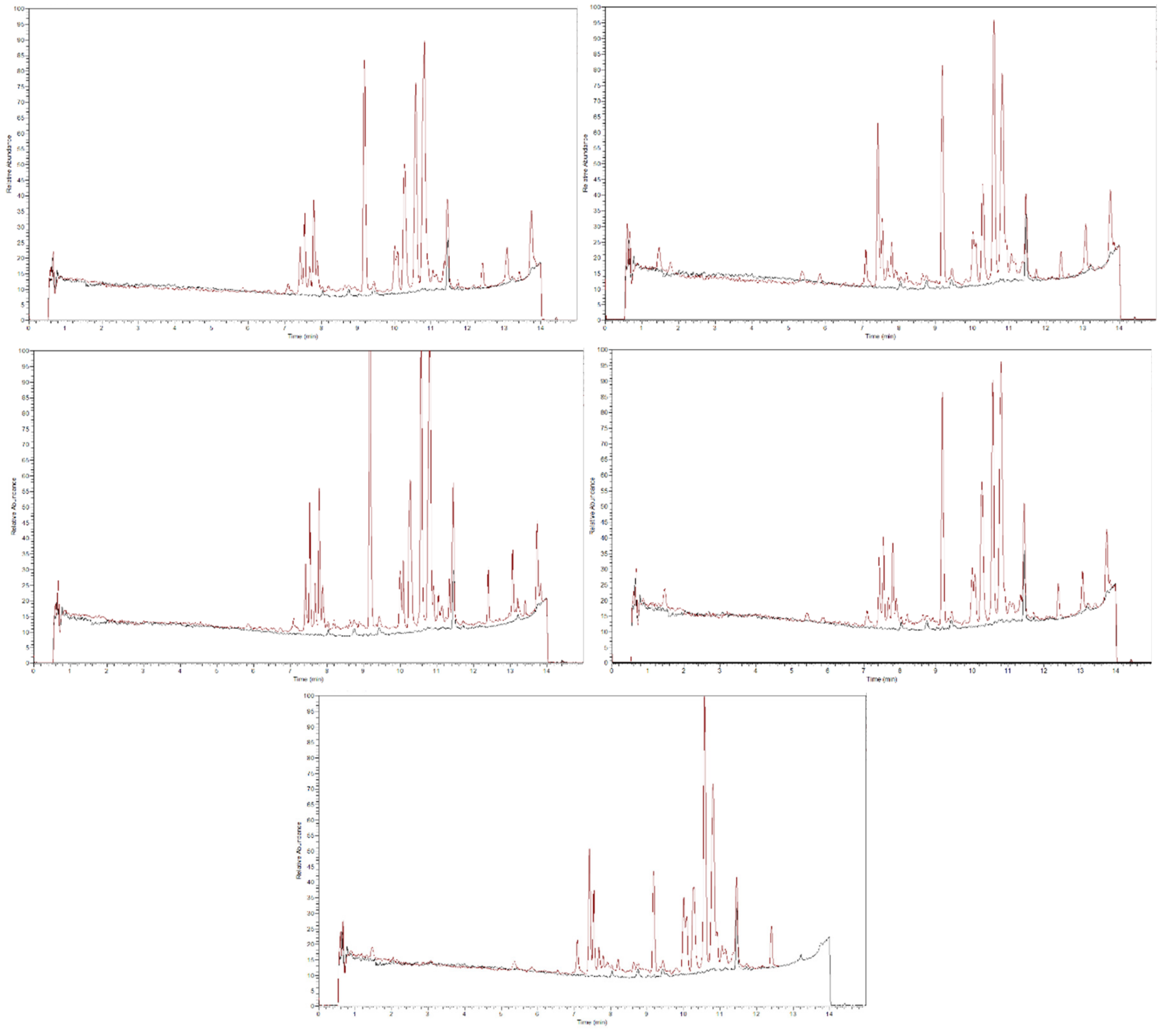

Figure S12. Typical negative mode LC-MS total ion chromatograms (TIC) of different *E. foeminea* crude extracts; **(a)** acetone; **(b)** dichloromethane/methanol; **(c)** ethanol; **(d)** ethyl acetate; **(e)** methanol. All chromatograms are displayed against the blank.

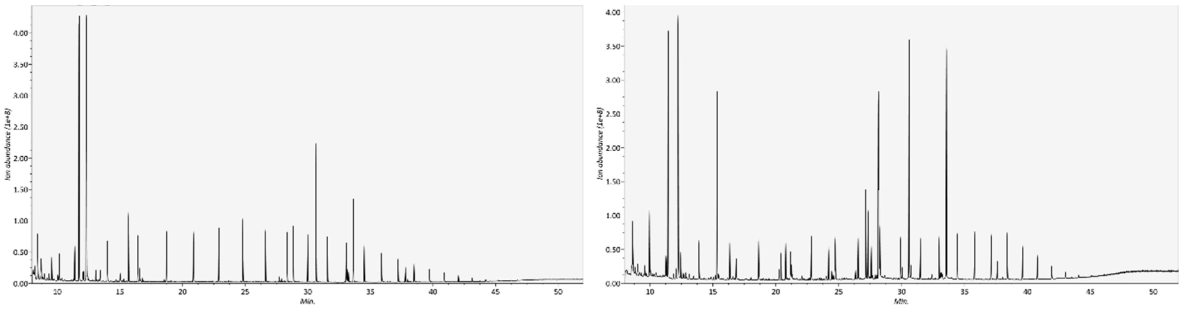

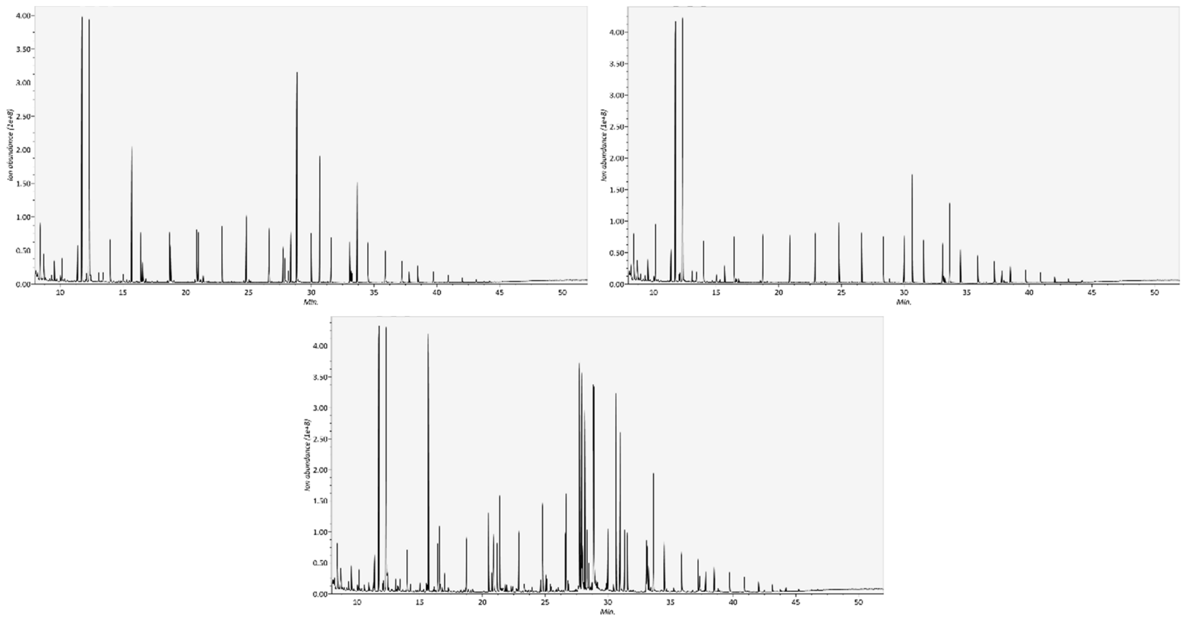

Figure S13. GC–MS Total Ion Chromatograms (TIC) of derivatized *E. foeminea* crude extracts using different solvents. **(a)** acetone; **(b)** dichloromethane/methanol; **(c)** ethanol; **(d)** ethyl acetate; **(e)** methanol.

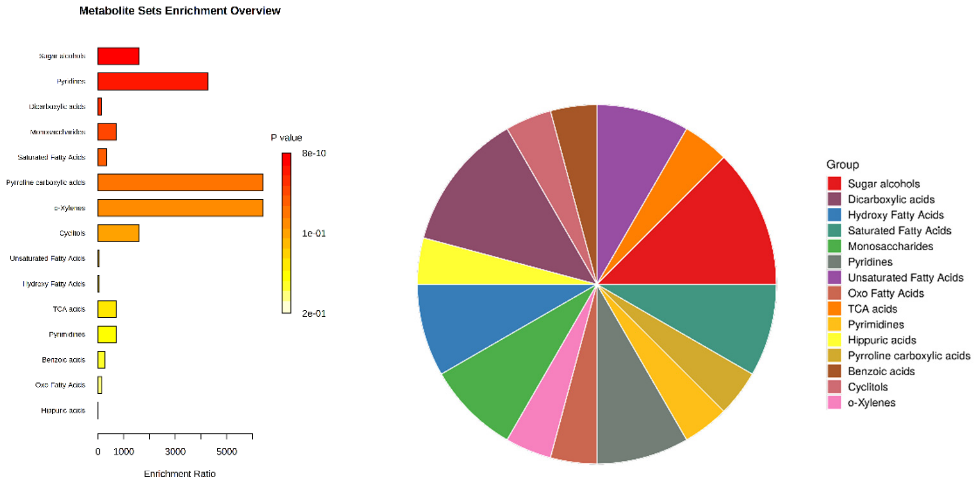

Figure S14. (a) Over Representation Analysis (ORA) Bar chart and **(b)** pie chart of the chemical classification of the total metabolites identified in the *E. foeminea* acetone crude extract sample using GC-MS via metabolite set enrichment analysis (MSEA). Colors in the bar plot describe the p-value. The red and orange colors signify the high and low values, respectively. The lines indicate the enrichment ratio, which was computed by hits/expected, where hits = observed hits and expected = expected hits. The colors in the interactive pie chart designate each chemical group relative to the total number of compounds.

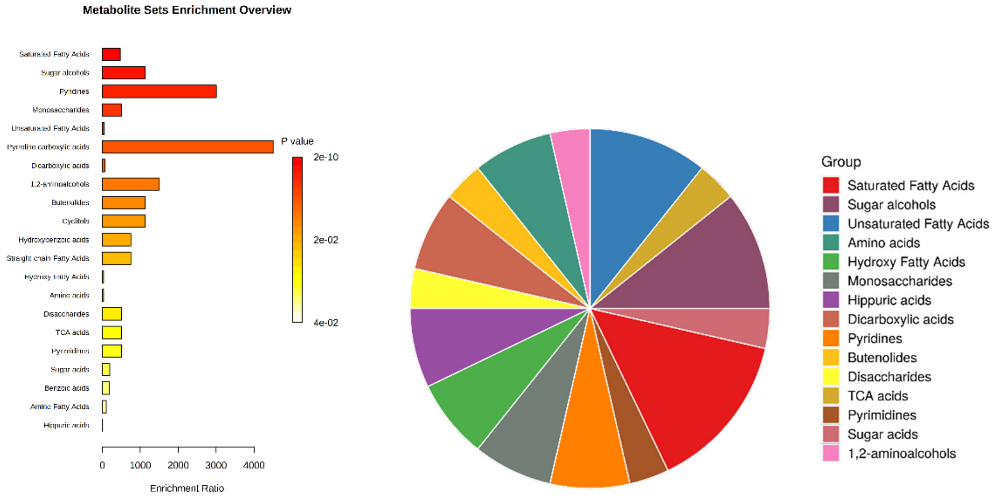

Figure S15. (a) Over Representation Analysis (ORA) Bar chart and **(b)** pie chart of the chemical classification of the total metabolites identified in the *E. foeminea* dichloromethane/methanol crude extract sample using GC-MS via metabolite set enrichment analysis (MSEA). Colors in the bar plot describe the p-value. The red and orange colors signify the high and low values, respectively. The lines indicate the enrichment ratio, which was computed by hits/expected, where hits = observed hits and expected = expected hits. The colors in the interactive pie chart designate each chemical group relative to the total number of compounds.

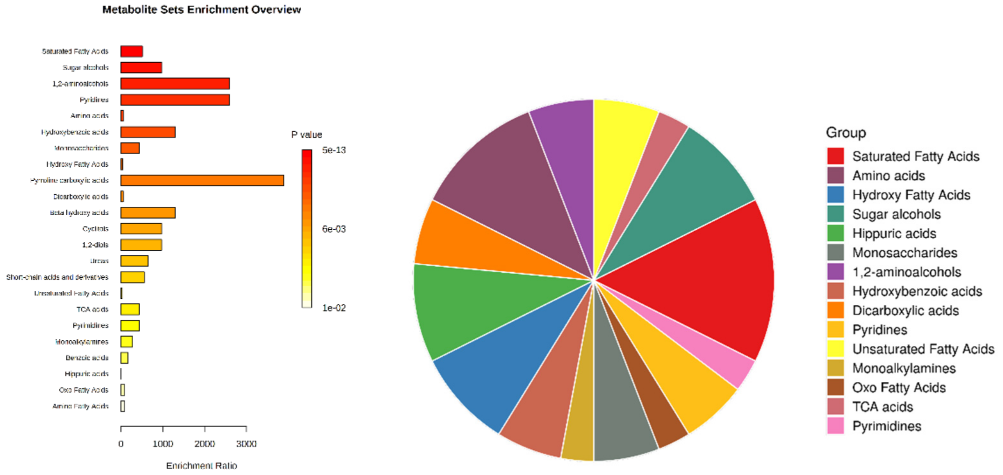

Figure S16. (a) Over Representation Analysis (ORA) Bar chart and **(b)** pie chart of the chemical classification of the total metabolites identified in the *E. foeminea* ethanol crude extract sample using GC-MS via metabolite set enrichment analysis (MSEA). Colors in the bar plot describe the p-value. The red and orange colors signify the high and low values, respectively. The lines indicate the enrichment ratio, which was computed by hits/expected, where hits = observed hits and expected = expected hits. The colors in the interactive pie chart designate each chemical group relative to the total number of compounds.

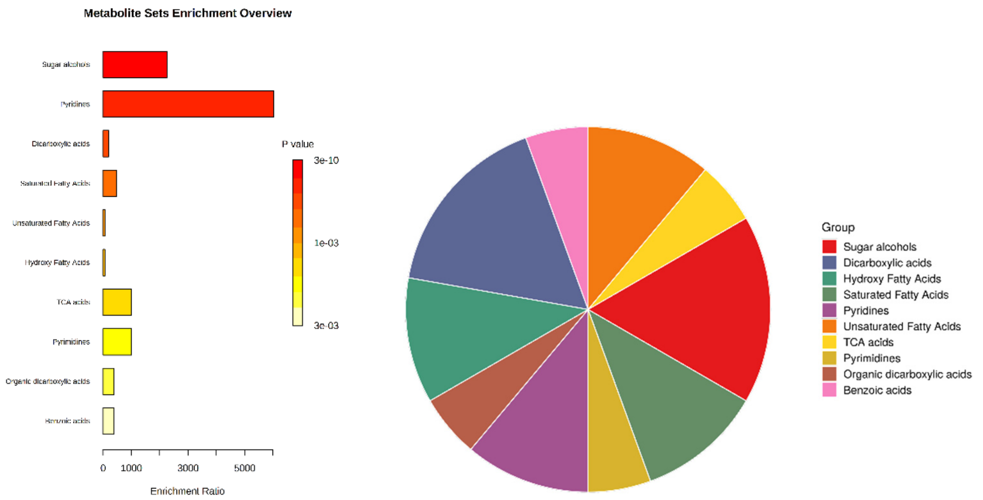

Figure S17. (a) Over Representation Analysis (ORA) Bar chart and **(b)** pie chart of the chemical classification of the total metabolites identified in the *E. foeminea* ethyl acetate crude extract sample using GC-MS via metabolite set enrichment analysis (MSEA). Colors in the bar plot describe the p-value. The red and orange colors signify the high and low values, respectively. The lines indicate the enrichment ratio, which was computed by hits/expected, where hits = observed hits and expected = expected hits. The colors in the interactive pie chart designate each chemical group relative to the total number of compounds.

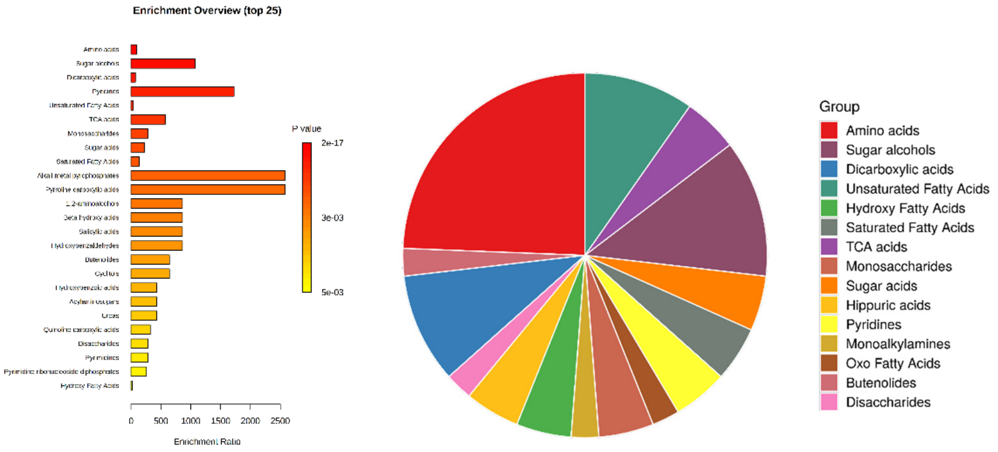

Figure S18. (a) Over Representation Analysis (ORA) Bar chart and **(b)** pie chart of the chemical classification of the total metabolites identified in the *E. foeminea* methanol crude extract sample using GC-MS via metabolite set enrichment analysis (MSEA). Colors in the bar plot describe the p-value. The red and orange colors signify the high and low values, respectively. The lines indicate the enrichment ratio, which was computed by hits/expected, where hits = observed hits and expected = expected hits. The colors in the interactive pie chart designate each chemical group relative to the total number of compounds.

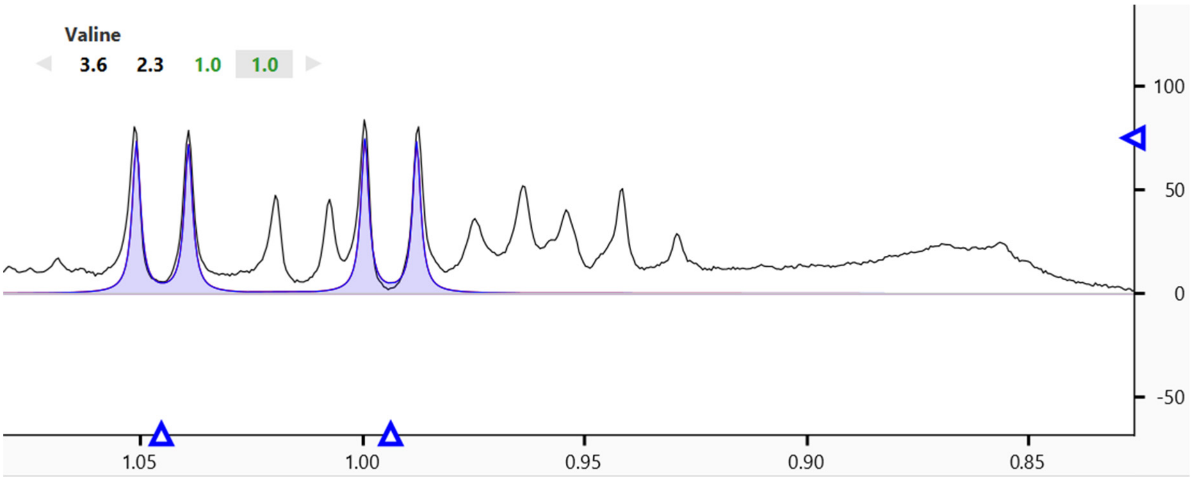

Figure S19. Screenshot of Chenomx Profiler (Chenomx Suite 9.0, Alberta, Canada) software window. As shown in the figure, the peaks at δ 1.05 ppm and 0.99 ppm can be assigned to valine.

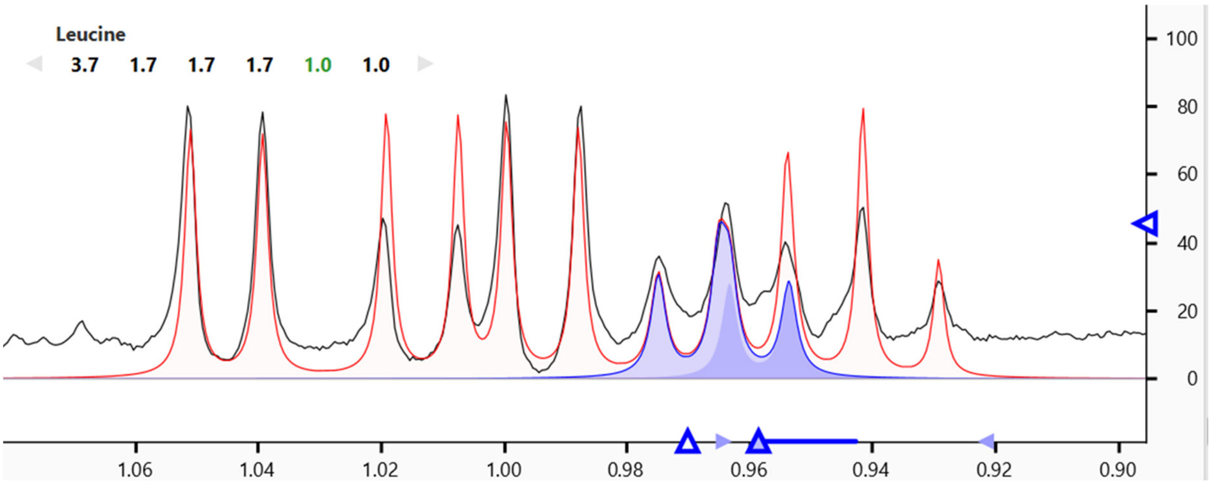

Figure S20. Screenshot of Chenomx Profiler (Chenomx Suite 9.0, Alberta, Canada) software window. As shown in the figure, the doublet at δ 0.96 ppm and 0.97 ppm can be assigned to leucine.

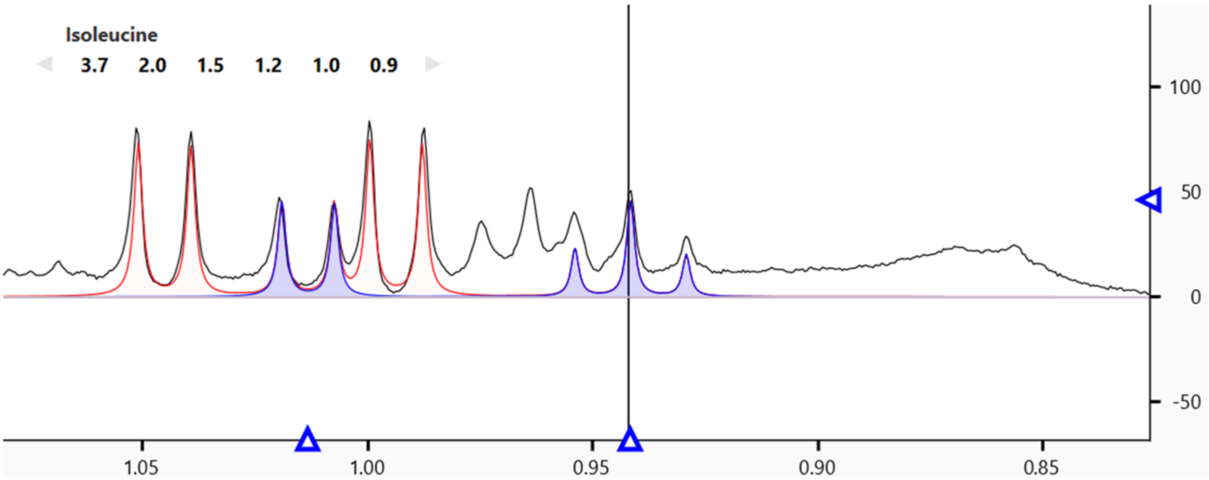

Figure S21. Screenshot of Chenomx Profiler (Chenomx Suite 9.0, Alberta, Canada) software window. As shown in the figure, the peaks at δ 1.1 ppm and 0.9 ppm can be assigned to isoleucine.

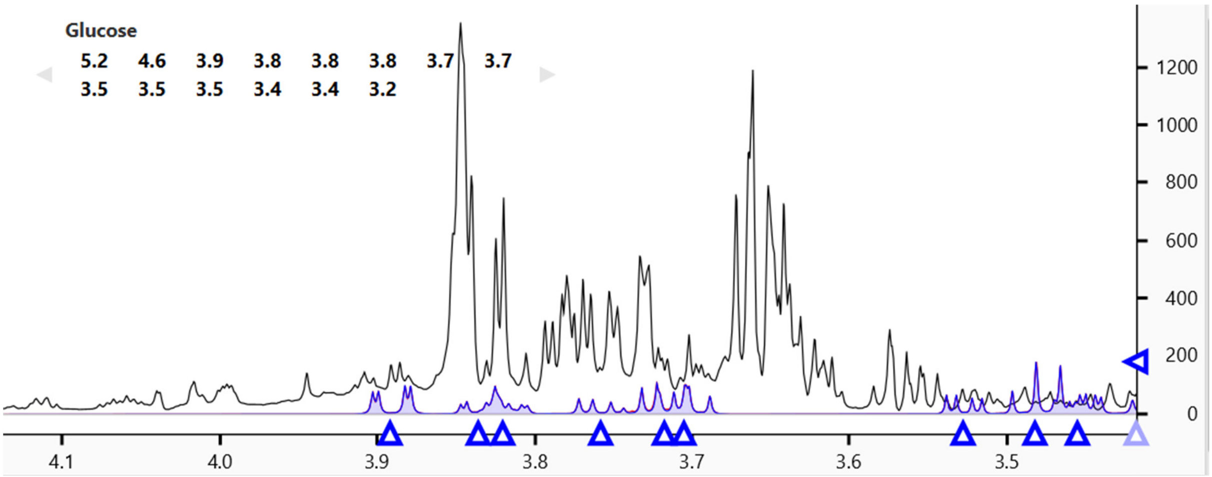

Figure S22. Screenshot of Chenomx Profiler (Chenomx Suite 9.0, Alberta, Canada) software window. As shown in the figure, the peaks mainly found between δ 3.2 ppm and 3.9 ppm can be assigned to glucose.

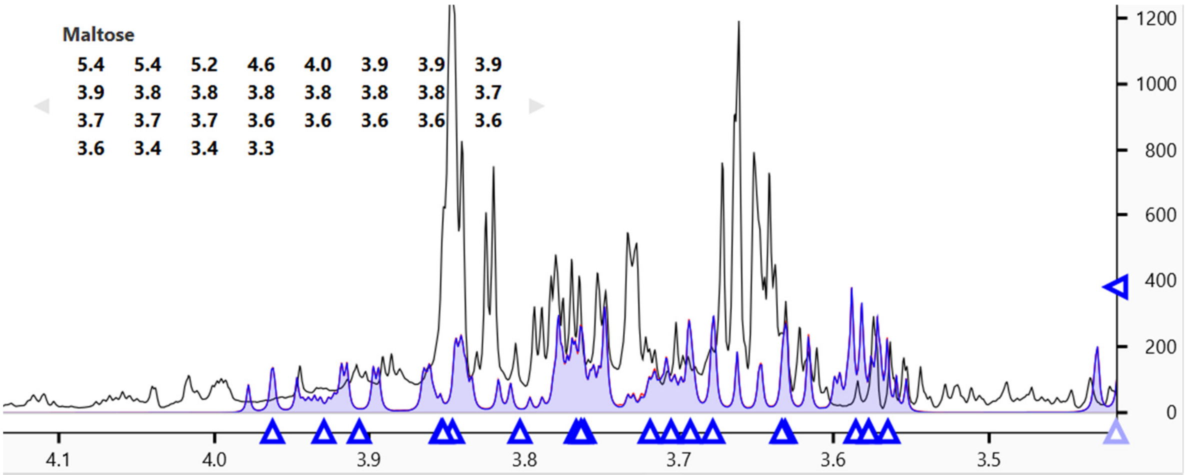

Figure S23. Screenshot of Chenomx Profiler (Chenomx Suite 9.0, Alberta, Canada) software window. As shown in the figure, the peaks mainly found between δ 3.4 ppm and 4.0 ppm can be assigned to maltose.

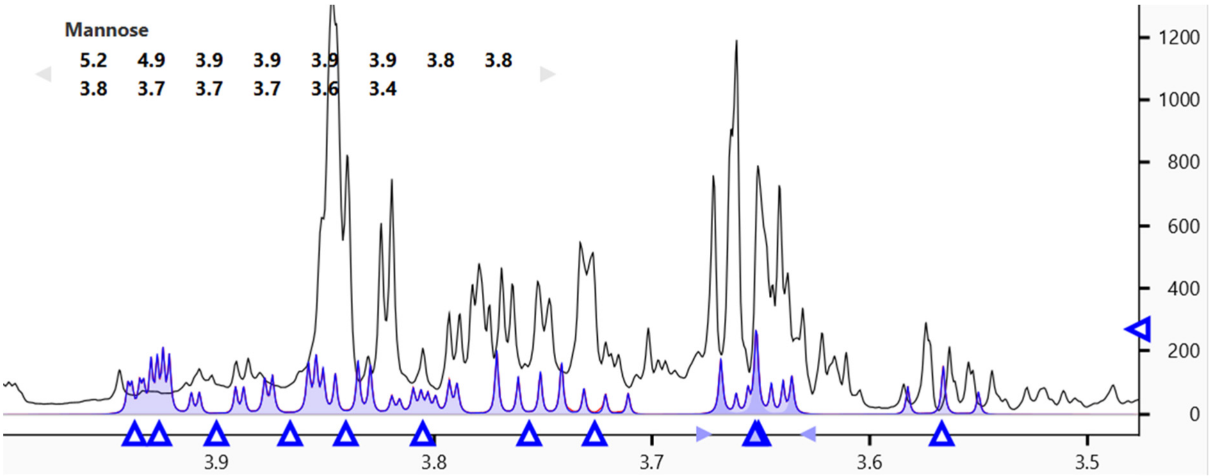

Figure S24. Screenshot of Chenomx Profiler (Chenomx Suite 9.0, Alberta, Canada) software window. As shown in the figure, the peaks mainly found between δ 3.4 ppm and 4.9 ppm can be assigned to mannose.

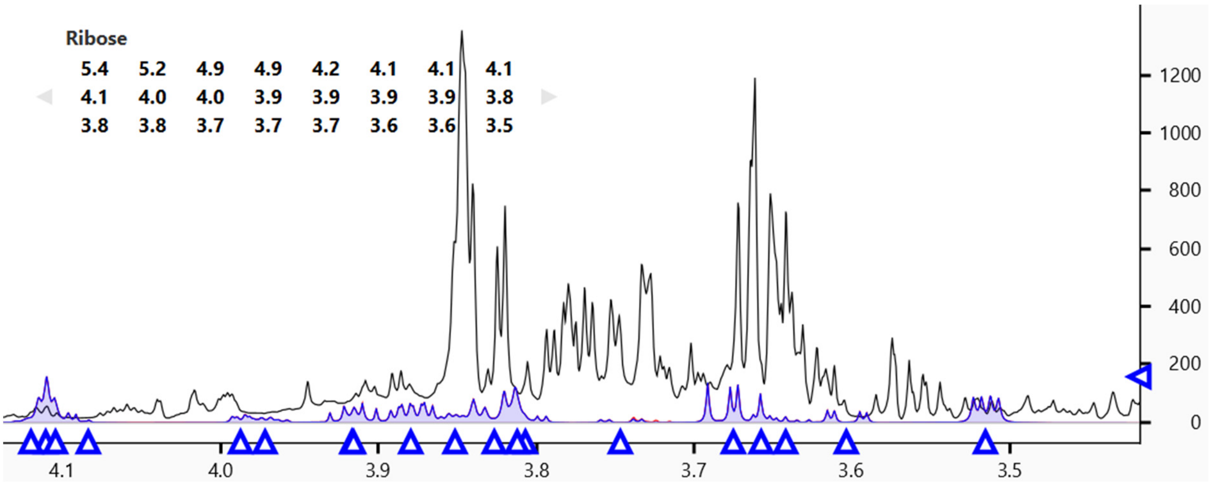

Figure S25. Screenshot of Chenomx Profiler (Chenomx Suite 9.0, Alberta, Canada) software window. As shown in the figure, the peaks mainly found between δ 3.5 ppm and 4.9 ppm can be assigned to ribose.

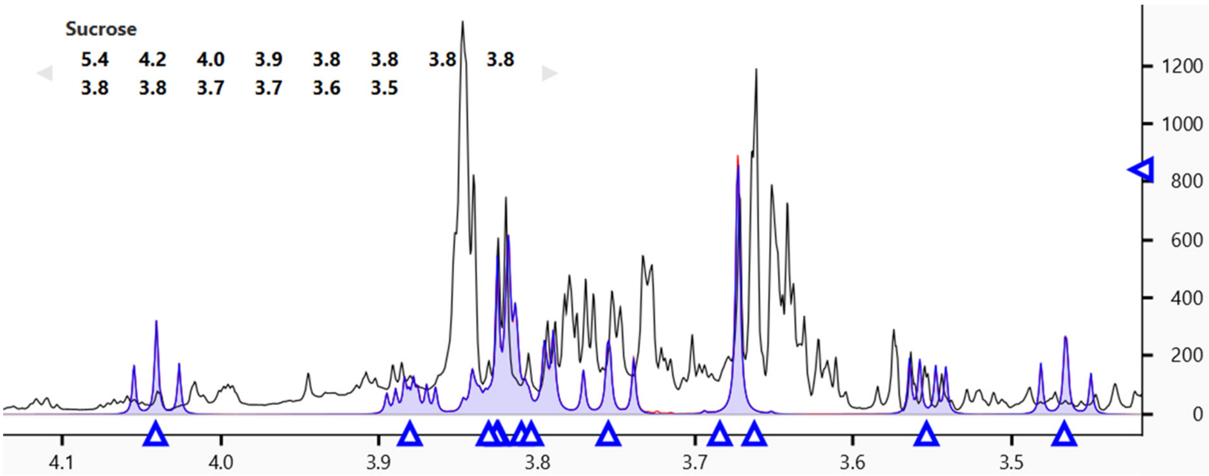

Figure S26. Screenshot of Chenomx Profiler (Chenomx Suite 9.0, Alberta, Canada) software window. As shown in the figure, the peaks mainly found between δ 3.5 ppm and 4.2 ppm can be assigned to sucrose.

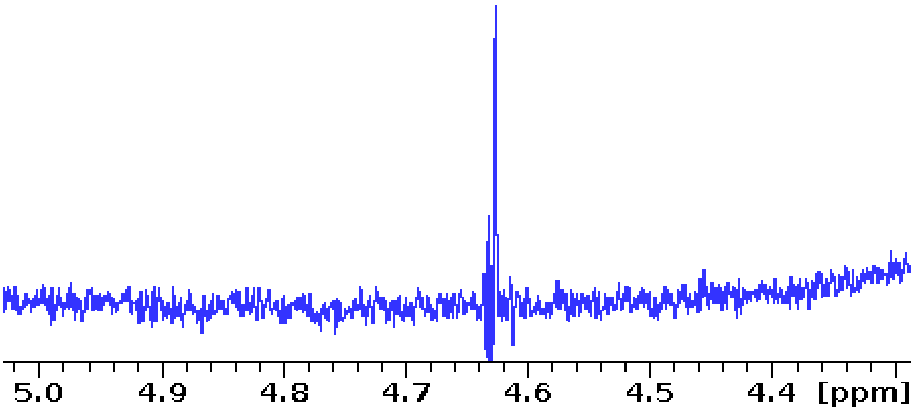

Figure S27. ^{1}H NMR 1D spectrum of *E. foeminea* methanolic extract recorded using 600 MHz liquid-NMR displaying the solvent region acquired with water suppression pulse program.

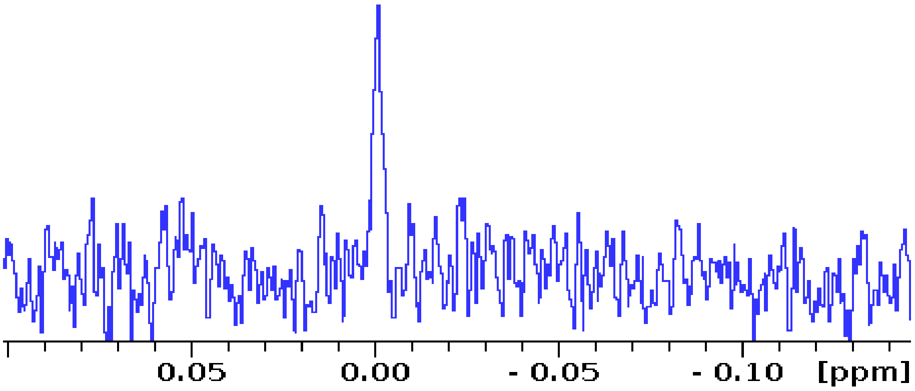

Figure S28. ^{1}H NMR 1D spectrum of *E. foeminea* methanolic extract recorded using 600 MHz liquid-NMR displaying the internal reference DSS region at δ 0.0 ppm.

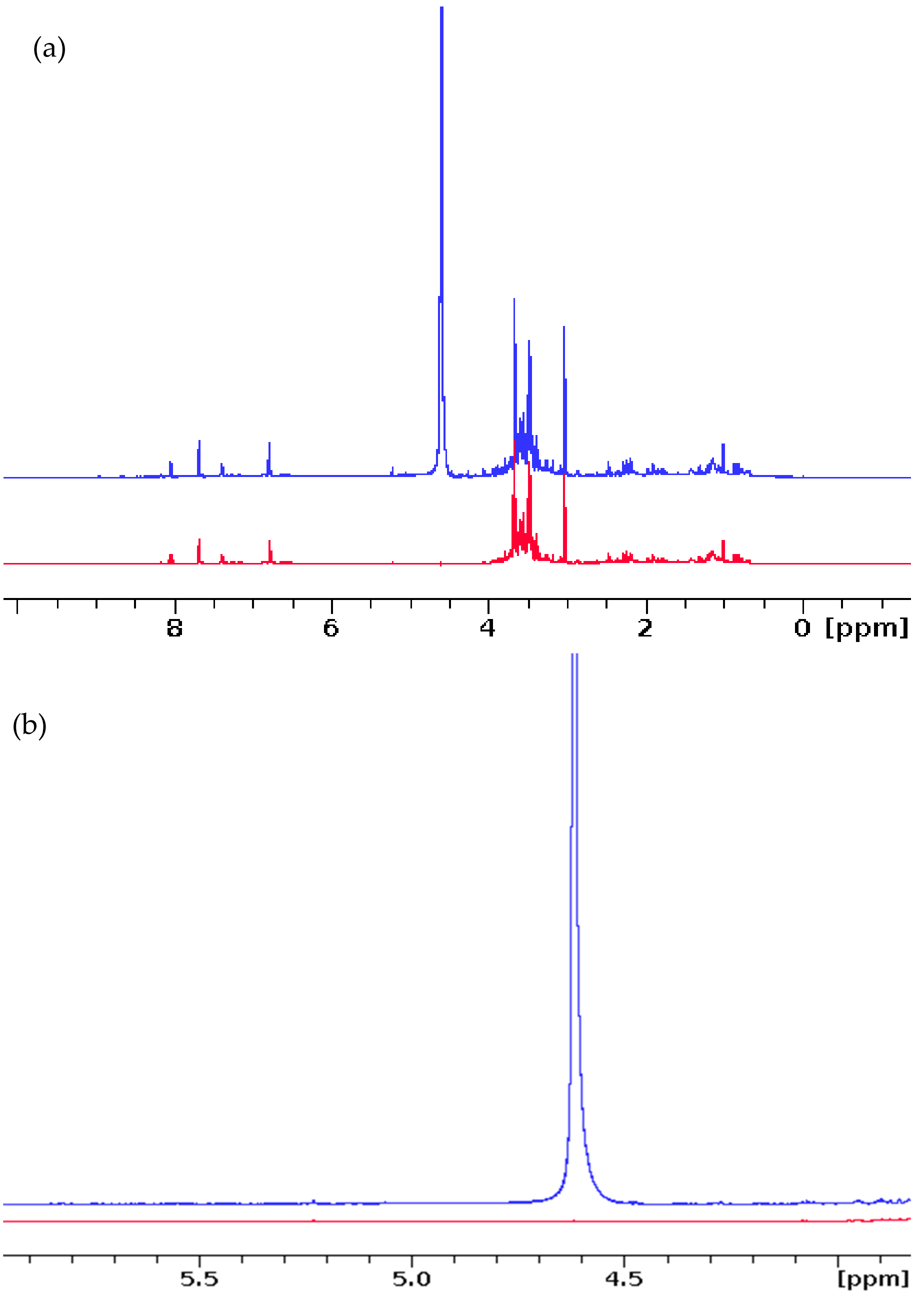

Figure S29. (a) ^{1}H NMR 1D spectrum of *E. foeminea* methanolic extract recorded using 600 MHz liquid-NMR with water suppression (red) and without water suppression (blue); (b) extended region. As shown in Figure S29a, the water peak is dominating the spectrum, thus we recorded all spectra using water suppression pulse program as described in materials and methods.
