# Supplementary material for: Untargeted Metabolomic Profiling and Antioxidant Capacities of Different Solvent Crude Extracts of Ephedra foeminea
Source: Metabolites. 2022 May 17;12(5):451. doi: 10.3390/metabo12050451 (PMC9146585; doi:10.3390/metabo12050451)
Supplement: Supplementary file 1 [file metabolites-12-00451-s001.zip › Supplementary Tables.pdf]

Table S1. Identified metabolites in *E. foeminea* crude extracts analyzed by positive mode LC–MS.

(a) acetone extract.

Compound Name	Formula	RT [min]
Unknown	C26 H49 F O	0.519
1-Methylpyrrolidin-2-one	C5 H9 N O	0.531
4,6-dimethylpyrimidin-2-amine	C6 H9 N3	0.531
Lanosta-8,24-dien-3beta,23-diol	C29 H48 O2	0.536
NP-021293	C30 H48 O4	0.536
2-Hydroxy-N,N,N-trimethylethan-1-aminium	C5 H13 N O	0.578
(3bS,5aS,7R,8R,10aR,10bS)-7-(Hydroxymethyl)-10b-methyl-3b,4,5,6,7,8,9,10,10a,10b,11,12-dodecahydro-5a,8-methanocyclohepta[5,6]naphtho[2,1-b]furan-7-ol	C5 H13 N O	0.583
Unknown	C4 H10 F2 N2 O5	0.591
4-(Hydroxyamino)-1-({1-hydroxy-1-[2-(hydroxymethyl)-1-pyrrolidinyl]-3-methyl-2-butanyl}amino)-2-pentyl-1,4-butanediol	C9 H8 N4 O2	0.595
2-Hydroxy-2-methylpropanenitrile	C5 H5 N5	0.626
cis-N-Butyl-4-(4-octylphenyl)cyclohexanamine	C26 H28 O14	0.626
1-Methylamino-1-(3,4-Methylenedioxyphenyl)Propane	C11 H15 N O2	0.627
3-(Dimethylamino)propyl acrylate	C8 H15 N O2	0.627
(2Z,4S,5S,6S,7R,8Z)-2,9-Diphenyldeca-2,8-diene-3,4,5,6,7,8-hexol	C11 H15 N O	0.627
exo-1,7,7-Trimethylbicyclo(2.2.1)hept-2-yl acrylate	C9 H17 N O2	0.627
4-Hydroxyquinoline-2-carboxylic acid	C10 H7 N O3	0.627
pyridine-3-carboxamide	C6 H6 N2 O	0.627
(10E,12E)-9-oxooctadeca-10,12-dienoic acid	C4 H7 N O	0.628
Unknown	C9 H17 F3 N4	0.629
2-Ethyl-2-oxazoline	C5 H9 N O	0.63
exo-1,7,7-Trimethylbicyclo(2.2.1)hept-2-yl acrylate	C13 H20 O2	0.63
2-Amino-6-({[(2-chlorobenzyl)oxy]carbonyl}amino)hexanoic acid	C14 H19 Cl N2 O4	0.631
2-Hydroxy-2-methylpropanenitrile	C4 H7 N O	0.897
2,3-dihydroxybutanedihydrazide	C4 H10 N4 O4	2.995
4-Hydroxyquinoline-2-carboxylic acid	C10 H7 N O3	3.237
Hexadecanamide	C12 H26 O7	3.598
3,6,9,12,15,18-Hexaoxaicosane-1,20-diol	C14 H30 O8	3.819
Unknown	C19 H35 F N3 O2 P	4.027
Unknown	C23 H45 N5 O8	4.533
Unknown	C25 H49 N5 O9	4.665
(2R,3S,4S,5S,6R)-2-({7-hydroxy-4-[(3Z)-5-hydroxy-3-methylpent-3-en-1-yl]-4a,8,8-trimethyl-3-methylidene-decahydronaphthalen-2-yl}oxy)-6-(hydroxymethyl)oxane-3,4,5-triol	C26 H44 O8	4.724
(2R,3S,4S,5S,6R)-2-({7-hydroxy-4-[(3Z)-5-hydroxy-3-methylpent-3-en-1-yl]-4a,8,8-trimethyl-3-methylidene-decahydronaphthalen-2-yl}oxy)-6-(hydroxymethyl)oxane-3,4,5-triol	C26 H44 O8	4.821
(2R,3S,4S,5S,6R)-2-({7-hydroxy-4-[(3Z)-5-hydroxy-3-methylpent-3-en-1-yl]-4a,8,8-trimethyl-3-methylidene-decahydronaphthalen-2-yl}oxy)-6-(hydroxymethyl)oxane-3,4,5-triol	C26 H44 O8	4.887
3-[(3S,4S,21R)-14-Ethyl-21-(methoxycarbonyl)-4,8,13,18-tetramethyl-20-oxo-9-vinyl-3-phorbinyl]propanoic acid	C20 H22 O7	6.045
NP-018660	C13 H22 O3	6.406
(11E,15Z)-9,10,13-trihydroxyoctadeca-11,15-dienoic acid	C18 H32 O5	6.519
NP-017152	C13 H23 N O2	6.658
3-(1′-Hydroxypentyl)phenol	C11 H16 O2	6.769
3-(4-Hydroxyphenyl)-1-(2,4,6-trihydroxyphenyl)propan-1-one	C15 H14 O5	6.798
chromen-2-one	C9 H6 O2	6.805

3,5,7-trihydroxy-2-(4-hydroxyphenyl)-4H-1-benzopyran-4-one	C15 H10 O6	6.805
(9Z)-Hexadec-9-enoic acid	C16 H30 O2	6.839
(9Z,11E,13S)-13-hydroperoxyoctadeca-9,11-dienoic acid	C18 H32 O4	6.84
2-[(3S)-1-(Cyclohexylmethyl)-3-pyrrolidinyl]-1H-benzimidazole-5-carbonitrile	C19 H24 N4	6.87
3-hydroxy-3,4-bis[(4-hydroxy-3-methoxyphenyl)methyl]oxolan-2-one	C16 H22	6.873
Decamethylcyclopentasiloxane	C16 H24 O	6.874
OPEO	C16 H26 O2	6.874
(4Z,6E,8R,10Z)-8-hydroxyhexadeca-4,6,10-trienoic acid	C16 H26 O3	6.884
NP-020214	C16 H32 O4	6.982
2,6-di-tert-butyl-4-ethylphenol	C16 H26 O	6.985
NP-020214	C16 H32 O4	6.985
5'-O-({1-[(2e)-4-(4-Hydroxy-6-Methoxy-7-Methyl-3-Oxo-1,3-Dihydro-2-Benzofuran-5-Yl)-2-Methylbut-2-En-1-Yl]-1h-1,2,3-Triazol-4-Yl}methyl)adenosine	C17 H26 N2 O S	7.009
NP-008563	C16 H28 O4	7.014
(4Z,6E,8R,10Z)-8-hydroxyhexadeca-4,6,10-trienoic acid	C16 H26 O3	7.014
1-[(2-Hydroxyethyl)amino]-2-dodecanol	C14 H31 N O2	7.022
N,N-Dimethyldodecan-1-amine N-oxide	C12 H27 N O	7.054
NP-015468	C20 H16 O4	7.133
(2S,3S,4R)-2-aminotetradecane-1,3,4-triol	C14 H31 N O3	7.158
OPEO	C16 H26 O2	7.188
2-[1-(2H-1,3-benzodioxol-5-yl)propan-2-yl]-6-methoxy-4-(prop-2-en-1-yl)phenol	C20 H22 O4	7.232
chromen-2-one	C13 H25 N O2	7.235
(Z)-docos-13-enamide	C13 H20 O2	7.377
Bicyclohexyliden-2-one	C22 H26 O6	7.807
Ethenylbenzene	C8 H8	7.807
(4Z,6E,8S,10Z)-8-hydroxyhexadeca-4,6,10-trienoic acid	C16 H26 O3	7.815
9*H*-Purin-6-amine	C20 H30 O5	7.96
(3E,4S)-3-[2-[(1R,4aS,5R,6R,8aS)-6-hydroxy-5-(hydroxymethyl)-5,8a-dimethyl-2-methylidene-3,4,4a,6,7,8-hexahydro-1H-naphthalen-1-yl]ethylidene]-4-hydroxyoxolan-2-one	C39 H32 O14	7.965
Unknown	C13 H25 F N8 O4 P2	7.965
5,7-dihydroxy-2-(4-hydroxyphenyl)-8-[3,4,5-trihydroxy-6-(hydroxymethyl)oxan-2-yl]-6-(3,4,5-trihydroxyoxan-2-yl)chromen-4-one	C9 H6 O2	7.966
6-cyclohexyl-5-(cyclohexylimino)-4-thia-6-azaspiro[2.4]heptan-7-one	C24 H24 O9	7.969
(11E,15Z)-9,10,13-trihydroxyoctadeca-11,15-dienoic acid	C18 H32 O5	7.971
6-O-[(2E)-3-(4-Hydroxyphenyl)-2-propenoyl]-1-O-[(2E)-3-phenyl-2-propenoyl]-β-D-glucopyranose	C24 H24 O9	8.014
chromen-2-one	C9 H6 O2	8.017
6-O-[(2E)-3-(4-Hydroxyphenyl)-2-propenoyl]-1-O-[(2E)-3-phenyl-2-propenoyl]-β-D-glucopyranose	C24 H24 O9	8.065
chromen-2-one	C9 H6 O2	8.065
6-O-[(2E)-3-(4-Hydroxyphenyl)-2-propenoyl]-1-O-[(2E)-3-phenyl-2-propenoyl]-β-D-glucopyranose	C24 H24 O9	8.134
chromen-2-one	C9 H6 O2	8.134
Hexadecanoic acid	C16 H32 O2	8.206
N,N-Dimethyldodecan-1-amine N-oxide	C14 H31 N O	8.255
(2S,3S,4R)-2-Aminooctadecane-1,3,4-triol	C18 H39 N O3	8.273
1-phenyldecan-1-one	C16 H35 N O3	8.315
N,N-Dimethyldodecan-1-amine N-oxide	C14 H31 N O	8.331
2-Aminohexadecanoic acid	C16 H33 N O2	8.438
Unknown	C32 H45 F O2 P2	8.473

(5alpha,17alpha)-3,3-Difluoropregn-20-yn-17-ol	C21 H30 F2 O	8.51
N,N-bis(2-hydroxyethyl)dodecan-1-amine oxide	C24 H30 O6	8.512
2-[1-(Aminomethyl)cyclohexyl]acetic acid	C16 H33 N O	8.582
N,N-Dimethyldodecan-1-amine N-oxide	C14 H31 N O	8.772
(9Z)-Hexadec-9-enoic acid	C16 H30 O2	9.139
(9Z)-Hexadec-9-enoic acid	C16 H30 O2	9.213
(2S,3R)-2-aminooctadecane-1,3-diol	C18 H39 N O2	9.354
NP-004713	C15 H24 O2	9.368
(3bS,5aS,7R,8R,10aR,10bS)-3b,4,5,6,7,8,9,10,10a,10b-Decahydro-7-hydroxy-10b-methyl-5a,8-Methano-5aH-cyclohepta(5,6)naphtho(2,1-b)furan-7-methanol	C20 H26 O3	9.371
Unknown	C31 H53 F3 N4 O	9.624
(9Z,11E,13E)-Octadeca-9,11,13-trienoic acid	C18 H30 O2	9.659
Unknown	C30 H57 F3 O5	9.696
NP-014287	C18 H32 O3	9.714
Unknown	C33 H56 N4 O3	9.798
Unknown	C32 H62 O7	9.866
Apigenin 7- (3'',6''-di-p-coumarylglucoside)	C12 H18 O	9.878
Unknown	C20 H28 O3	9.878
N-{[(2R,4S,5R)-5-Vinyl-1-azabicyclo[2.2.2]oct-2-yl]methyl}cyclohexanecarboxamide	C17 H28 N2 O	9.878
(9Z)-Hexadec-9-enoic acid	C16 H30 O2	9.878
(9Z,11E,13S)-13-hydroperoxyoctadeca-9,11-dienoic acid	C18 H32 O4	9.949
(9Z)-Hexadec-9-enoic acid	C16 H30 O2	9.949
Unknown	C34 H54 F2 N4	9.982
(3bS,5aS,7R,8R,10aR,10bS)-7-(Hydroxymethyl)-10b-methyl-3b,4,5,6,7,8,9,10,10a,10b,11,12-dodecahydro-5a,8-methanocyclohepta[5,6]naphtho[2,1-b]furan-7-ol	C20 H28 O3	10.037
N-{[(2R,4S,5R)-5-Vinyl-1-azabicyclo[2.2.2]oct-2-yl]methyl}cyclohexanecarboxamide	C17 H28 N2 O	10.037
(9Z)-Hexadec-9-enoic acid	C16 H30 O2	10.037
Unknown	C32 H62 O7	10.061
6-O-[(2E)-3-(4-Hydroxyphenyl)-2-propenoyl]-1-O-[(2E)-3-phenyl-2-propenoyl]-β-D-glucopyranose	C18 H30 O3	10.179
(9Z)-Hexadec-9-enoic acid	C16 H30 O2	10.179
N-{[(2R,4S,5R)-5-Vinyl-1-azabicyclo[2.2.2]oct-2-yl]methyl}cyclohexanecarboxamide	C17 H28 N2 O	10.181
NP-020014	C15 H26 O3	10.229
(9Z,12Z)-N-(2-hydroxyethyl)octadeca-9,12-dienamide	C20 H37 N O2	10.761
Unknown	C20 H31 F3 O6	10.874
4-[(1R,3S,5S,8R,9S,10R,11R,13R,14S,17R)-1,5,11,14-tetrahydroxy-10-(hydroxymethyl)-13-methyl-3-((2R,3R,4R,5R,6S)-3,4,5-trihydroxy-6-methyltetrahydro-2H- pyran-2-yloxy)hexadecahydro-1H-cyclopenta[a]phenanthren-17-yl]furan-2(5H)-one	C29 H44 O12	10.948
3-[(3R,12Z,21S,22S)-16-ethenyl-11-ethyl-12-(hydroxymethylidene)-3-methoxycarbonyl-17,21,26-trimethyl-4-oxo-7,23,24,25-tetrazahexacyclo[18.2.1.15,8.110,13.115,18.02,6]hexacosa-1,5(26),6,8,10,13(25),14,16,18(24),19-decaen-22-yl]propanoic acid	C35 H34 N4 O6	11.168
Unknown	C25 H39 F2 N	11.234
N,N-bis(2-hydroxyethyl)hexadecanamide	C18 H37 N O2	11.276
3-Methyl-1-phenyl-1,2-nonadiene	C19 H41 N3 O5	11.312
2-(methylamino)-1-phenylbutan-1-one	C12 H15 Cl2 F2 N6 O2 P	11.398
2-Hydroxy-N,N,N-trimethylethan-1-aminium	C24 H41 N	11.42
(9Z)-N-(2-Hydroxyethyl)octadec-9-enamide	C20 H39 N O2	11.522
Unknown	C21 H42 F N O	11.623

Unknown	C24 H41 F2 N6 O4 P3	11.86
methyl (1R,15S,17R,18R,19S,20S)-6,18-dimethoxy-17-(3,4,5-trimethoxybenzoyl)oxy-1,3,11,12,14,15,16,17,18,19,20,21-dodecahydroyohimban-19-carboxylate	C33 H40 N2 O9	11.86
Unknown	C32 H49 F N3 O8 P	11.91
Unknown	C40 H50 F3 N2 O3 P	11.91
5-hydroxy-3-methyl-N-{3-[(2R,5R)-5-{3-[(2E)-3-methyl-5-{[(2S,3R,4R,5S,6S)-3,4,5-trihydroxy-6-(hydroxymethyl)oxan-2-yl]oxy}pent-2-enamido]propyl}-3,6-dioxopiperazin-2-yl]propyl}pent-2-enamide	C28 H46 N4 O11	11.91
4-Oxo-3,4-dihydroquinazoline-2-carbohydrazide	C28 H32 N8 O8	12.147
methyl (1R,15S,17R,18R,19S,20S)-6,18-dimethoxy-17-(3,4,5-trimethoxybenzoyl)oxy-1,3,11,12,14,15,16,17,18,19,20,21-dodecahydroyohimban-19-carboxylate	C33 H40 N2 O9	12.152
2-(dimethylamino)ethyl 3-cyclohexylpropanoate	C10 H30 O5 Si5	12.273
1-{2-[(11aS)-5-(4-Isopropylphenyl)-1,3-dioxo-5,6,11,11a-tetrahydro-1H-imidazo[1',5':1,6]pyrido[3,4-b]indol-2(3H)-yl]benzoyl}-4-piperidinecarboxylic acid	C35 H34 N4 O5	12.344
Unknown	C17 H43 F5 N10 O3 P2	12.36
2-(Allyl{4-[(2-ethyl-5,7-dimethyl-3H-imidazo[4,5-b]pyridin-3-yl)methyl]phenyl}amino)-2-phenyl-N-(phenylsulfonyl)acetamide	C34 H35 N5 O3 S	12.363
3,6,9,12,15-Pentaoxaheptadecan-1,17-diol	C35 H36 N4 O5	12.365
3-[(3S,4S,21R)-14-Ethyl-21-(methoxycarbonyl)-4,8,13,18-tetramethyl-20-oxo-9-vinyl-3-phorbinyl]propanoic acid	C35 H36 N4 O5	12.549
Unknown	C20 H44 N6 O14	12.549
N-[2-(3,4-Dimethoxyphenyl)ethyl]-2-({2-[2-(1-piperidinyl)ethyl][1,2,4]triazolo[1,5-c]quinazolin-5-yl}sulfanyl)acetamide	C28 H34 N6 O3 S	12.702
1'-ethyl-5-[2'-methyl-4'-(5-methyl-1,3,4-oxadiazolyl-2-yl)biphenyl-4-carbonyl]-2,3,6,7-tetrahydrospiro(furo[2,3-f]indole-3,4'-piperidine)	C33 H34 N4 O3	12.705
(2E,4S)-4-[(2-Oxohexadecanoyl)amino]-2-octenoic acid	C24 H43 N O4	12.802
1-isothiocyanatooctane	C9 H17 N S	12.947
(1R,9S)-5-[(E)-2-(4-Chlorophenyl)vinyl]-11-(5-pyrimidinylcarbonyl)-7,11-diazatricyclo[7.3.1.02,7]trideca-2,4-dien-6-one	C24 H21 Cl N4 O2	12.948
N-[(2S,3R)-1,3-dihydroxyoctadecan-2-yl]dodecanamide	C30 H61 N O3	13.034
Decamethylcyclopentasiloxane	C10 H30 O5 Si5	13.091
N-{4-[4-(4-Chlorophenyl)-1-piperazinyl]benzyl}-2-(dibenzo[b,d]thiophen-2-yl)imidazo[1,2-a]pyridine-3-carboxamide	C37 H30 Cl N5 O S	13.091
Unknown	C26 H33 Cl F N7 O3 S2	13.462
Unknown	C36 H57 F3 O3	13.484
N-(2-Aminoethyl)-N'-(1,5-dicyclohexyl-3-pentanyl)-1,2-ethanediamine	C21 H43 N3	13.635
methyl (4aS,4bR,5aR,6aS,7R,9aS,9bR,10R)-4a,6a-dimethyl-2,5'-dioxo-2,4,4',4a,5',5a,6,6a,8,9,9a,9b,10,11-tetradecahydro-3H,3'H-spiro[cyclopenta[7,8]phenanthro[4b,5-b]oxirene-7,2'-furan]-10-carboxylate	C22 H43 N O	13.636
Unknown	C34 H61 N O5	13.733
NP-014287	C18 H32 O3	13.751
Unknown	C44 H35 Cl2 F2 N O	13.757
Unknown	C41 H78 F N O4 P2	13.78
Unknown	C34 H57 F3 O3	13.946

(b) dichloromethane/methanol extract.

Compound Name	Formula	RT [min]
4-methylpyridine-2,3-diamine	C6 H9 N3	1.027
5,7-Diisopropyl-1,1-dimethyl-1,2,3,4-tetrahydronaphthalene	C18 H28	1.028
Unknown	C25 H53 F3 N4 O4	1.028
Unknown	C3 H F4 P	1.028
Unknown	C31 H31 Cl F N S2	1.028
Unknown	C38 H65 F3 O4	1.028

Unknown	C5 H F2 O P	1.028
Unknown	C51 H80 F2 O	1.028
NP-020521	C18 H32 O2	1.028
Unknown	C12 H12 Cl F N10 S2	1.029
Unknown	C38 H71 N O6	1.029
Unknown	C40 H75 N O6	1.029
2-acetamido-3-sulfanylpropanoic acid	C5 H9 N O3 S	1.03
1-hydroxy-2,2,6,6-tetramethylpiperidin-4-ol	C9 H19 N O2	1.4
2,3-dihydroxybutanedihydrazide	C4 H10 N4 O4	3.022
4-Hydroxyquinoline-2-carboxylic acid	C10 H7 N O3	3.199
Unknown	C3 H10 F2 N3 O2 P	3.264
4-Hydroxyquinoline-2-carboxylic acid	C10 H7 N O3	3.265
1-BENZYL-4-PROPYLAMINO-4-PIPERIDINECARBOXAMIDE	C16 H25 N3 O	3.352
Unknown	C15 H29 N5 O4	3.811
1-{[4-(2,5-dimethyl-1H-pyrrol-1-yl)benzoyl]oxy}dihydro-1H-pyrrole-2,5-dione	C17 H16 N2 O4	3.832
Unknown	C17 H33 N5 O5	3.993
Unknown	C18 H41 F3 N7 O4 P	4.622
(2R,3S,4S,5S,6R)-2-({7-hydroxy-4-[(3Z)-5-hydroxy-3-methylpent-3-en-1-yl]-4a,8,8-trimethyl-3-methylidene-decahydronaphthalen-2-yl}oxy)-6-(hydroxymethyl)oxane-3,4,5-triol	C26 H44 O8	4.671
4-Isopropylbenzaldehyde	C10 H12 O	5.42
2-(dibutylamino)ethanol	C10 H23 N O	5.46
2-(Hydroxymethyl)-2-(octylamino)-1,3-propanediol	C12 H27 N O3	5.624
3,5,7-Trimethyl-1-azatricyclo[3.3.1.1~3,7~]decane-2,8-diol	C12 H21 N O2	5.857
(9aR,9bS)-9a-Hydroxy-6,9-dimethyl-3-methylene-3,3a,4,5,9a,9b-hexahydroazuleno[4,5-b]furan-2,7-dione	C15 H16 O4	5.879
Unknown	C21 H37 F4 N O9	5.887
NP-018660	C13 H22 O3	6.128
(11E,15Z)-9,10,13-trihydroxyoctadeca-11,15-dienoic acid	C18 H32 O5	6.208
8-(4-oxo-5-pent-2-enylcyclopent-2-en-1-yl)octanoic acid	C18 H28 O3	6.208
8-[(1S,5R)-4-oxo-5-[(Z)-pent-2-enyl]cyclopent-2-en-1-yl]octanoic acid	C18 H28 O3	6.208
4-[(1R,3S,5S,8R,9S,10R,11R,13R,14S,17R)-1,5,11,14-tetrahydroxy-10-(hydroxymethyl)-13-methyl-3-((2R,3R,4R,5R,6S)-3,4,5-trihydroxy-6-methyltetrahydro-2H- pyran-2-yloxy)hexadecahydro-1H-cyclopenta[a]phenanthren-17-yl]furan-2(5H)-one	C15 H23 N O3	6.334
5-(2-Ethoxyphenyl)-1-methyl-3-propyl-1,6-dihydro-7H-pyrazolo[4,3-d]pyrimidin-7-one	C17 H20 N4 O2	6.356
Unknown	C16 H33 N O6	6.359
chromen-2-one	C9 H6 O2	6.462
3-Butyl-3a,4,5,6-tetrahydro-2-benzofuran-1(3H)-one	C12 H18 O2	6.495
(6S)-6-[(1Z)-But-1-en-1-yl]cyclohepta-1,4-diene	C11 H16	6.496
(12Z)-9,10,11-trihydroxyoctadec-12-enoic acid	C18 H34 O5	6.498
(9Z)-Hexadec-9-enoic acid	C16 H30 O2	6.5
(9Z,11E,13S)-13-hydroperoxyoctadeca-9,11-dienoic acid	C18 H32 O4	6.501
(4aR,9aS)-7-Ethynyl-4a,9a-dimethyl-1-methylene-2,3,4,4a,5,6,9,9a-octahydro-1H-benzo[7]annulene	C16 H22	6.535
1-phenyldecan-1-one	C16 H24 O	6.535
(S)-1-[3-(Dimethylamino)propyl]-1-(4-fluorophenyl)-1,3-dihydroisobenzofuran-5-carbonitrile	C20 H21 F N2 O	6.535
(9Z)-N-(2-Hydroxyethyl)octadec-9-enamide	C16 H26 O2	6.535
(4Z,6E,8R,10Z)-8-hydroxyhexadeca-4,6,10-trienoic acid	C16 H26 O3	6.541
NP-020214	C16 H32 O4	6.636
2,6-di-tert-butyl-4-ethylphenol	C16 H26 O	6.637

NP-020014	C16 H32 O4	6.637
1-[(2-Hydroxyethyl)amino]-2-dodecanol	C14 H31 N O2	6.663
3-[2-carboxyethyl(dodecyl)amino]propanoic acid	C18 H35 N O4	6.667
6-cyclohexyl-5-(cyclohexylimino)-4-thia-6-azaspiro[2.4]heptan-7-one	C17 H26 N2 O S	6.671
NP-006888	C16 H28 O4	6.673
(4Z,6E,8R,10Z)-8-hydroxyhexadeca-4,6,10-trienoic acid	C16 H26 O3	6.674
N,N-Dimethyldodecan-1-amine N-oxide	C12 H27 N O	6.692
1-hydroxy-2,2,6,6-tetramethylpiperidine (TEMPO) (TEMPO)	C9 H19 N O	6.745
(2S,3S,4R)-2-aminotetradecane-1,3,4-triol	C14 H31 N O3	6.789
OPEO	C16 H26 O2	6.832
(2Z)-2-Hepten-1-ylbenzene	C13 H18	6.873
2-[1-(2H-1,3-benzodioxol-5-yl)propan-2-yl]-6-methoxy-4-(prop-2-en-1-yl)phenol	C20 H22 O4	6.876
(E)-4-(2,6,6-trimethylcyclohexen-1-yl)but-3-en-2-one	C13 H20 O	6.876
Unknown	C32 H34 N3 P	6.876
2-(dimethylamino)ethyl 3-cyclohexylpropanoate	C13 H25 N O2	6.878
NP-018660	C13 H22 O3	7.027
Unknown	C16 H27 F3 O2	7.046
(1R,4aS)-7-(2-Hydroxypropan-2-yl)-1,4a-dimethyl-9-oxo-3,4,10,10a-tetrahydro-2H-phenanthrene-1-carboxylic acid	C20 H26 O4	7.049
Unknown	C10 H23 F N8 O	7.064
2-aminoethyl (allylamino)methanedithioate hydrochloride	C6 H12 N2 S2	7.175
Unknown	C13 H25 F N6 O5	7.25
Unknown	C16 H29 F N2 O3 S2	7.25
5-hydroxy-3-methyl-N-{3-[(2R,5R)-5-{3-[(2E)-3-methyl-5-{[(2S,3R,4R,5S,6S)-3,4,5-trihydroxy-6-(hydroxymethyl)oxan-2-yl]oxy}pent-2-enamido]propyl}-3,6-dioxopiperazin-2-yl]propyl}pent-2-enamide	C15 H26 O3	7.308
NP-016596	C13 H22 O3	7.317
NP-014287	C19 H30 O8	7.411
1-phenylethanone	C8 H8 O	7.412
3-{(4R,7S,8aS)-7-[(Isopropylcarbamoyl)amino]-1-oxooctahydropyrrolo[1,2-a]pyrazin-4-yl}-N-(4-methoxybenzyl)propanamide	C22 H33 N5 O4	7.414
(2Z,4S,5S,6S,7R,8Z)-2,9-Diphenyldeca-2,8-diene-3,4,5,6,7,8-hexol	C22 H26 O6	7.415
Unknown	C12 H34 N9 O4 P S	7.415
Unknown	C22 H28 F5 N5	7.415
(1S,2S,4R,8S,9S,11S,12R,13S,19S)-12,19-difluoro-11-hydroxy-8-(2-hydroxyacetyl)-6,6,9,13-tetramethyl-5,7-dioxapentacyclo[10.8.0.02,9.04,8.013,18]icosa-14,17-dien-16-one	C21 H26 F2 O6	7.415
Ethenylbenzene	C8 H8	7.415
N,N'-(2-Hydroxy-1,3-propanediyl)dioctanamide	C19 H38 N2 O3	7.462
[(3S,4S,6S)-3,4-dihydroxy-6-[5-hydroxy-2-(4-hydroxyphenyl)-4-oxochromen-7-yl]oxy-5-[(E)-3-(4-hydroxyphenyl)prop-2-enoyl]oxyoxan-2-yl]methyl (E)-3-(4-hydroxyphenyl)prop-2-enoate	C39 H32 O14	7.537
8-[(1S,5R)-4-oxo-5-[(Z)-pent-2-enyl]cyclopent-2-en-1-yl]octanoic acid	C18 H28 O3	7.538
(3E,4S)-3-[2-[(1R,4aS,5R,6R,8aS)-6-hydroxy-5-(hydroxymethyl)-5,8a-dimethyl-2-methylidene-3,4,4a,6,7,8-hexahydro-1H-naphthalen-1-yl]ethylidene]-4-hydroxyoxolan-2-one	C20 H30 O5	7.538
N-(6-Amino-1-isobutyl-2,4-dioxo-1,2,3,4-tetrahydro-5-pyrimidinyl)-3-cyclohexyl-N-isobutylpropanamide	C21 H36 N4 O3	7.539
8-(4-oxo-5-pent-2-enylcyclopent-2-en-1-yl)octanoic acid	C18 H28 O3	7.54
Unknown	C13 H25 F N8 O4 P2	7.54
6-O-[(2E)-3-(4-Hydroxyphenyl)-2-propenoyl]-1-O-[(2E)-3-phenyl-2-propenoyl]-β-D-glucopyranose	C24 H24 O9	7.544
(E)-3-(2-hydroxyphenyl)prop-2-enoic acid (o-coumaric acid)	C9 H8 O3	7.547
N,N-Bis(2-hydroxyethyl)dodecanamide	C16 H33 N O3	7.548

Unknown	C28 H62 F N5 O8	7.572
(E)-3-(2-hydroxyphenyl)prop-2-enoic acid	C9 H8 O3	7.634
(E)-3-(2-hydroxyphenyl)prop-2-enoic acid	C9 H8 O3	7.694
6-O-[(2E)-3-(4-Hydroxyphenyl)-2-propenoyl]-1-O-[(2E)-3-phenyl-2-propenoyl]-β-D-glucopyranose	C24 H24 O9	7.694
[(3S,4S,6S)-3,4-dihydroxy-6-[5-hydroxy-2-(4-hydroxyphenyl)-4-oxochromen-7-yl]oxy-5-[(E)-3-(4-hydroxyphenyl)prop-2-enoyl]oxyoxan-2-yl]methyl (E)-3-(4-hydroxyphenyl)prop-2-enoate	C39 H32 O14	7.694
8-(4-oxo-5-pent-2-enylcyclopent-2-en-1-yl)octanoic acid	C18 H28 O3	7.73
(RS)-1-[2-(Allyloxy)phenoxy]-3-(isopropylamino)propan-2-ol	C16 H32 O2	7.763
Unknown	C15 H34 N2 O2	7.766
N-[(2S,3S)-3-Fluoro-1-hydroxy-2-dodecanyl]hexanamide	C18 H36 F N O2	7.767
N,N-Dimethyldodecan-1-amine N-oxide	C14 H31 N O	7.805
NP-018660	C13 H22 O3	7.817
C16 (2S,3S,4R)-2-Aminooctadecane-1,3,4-triol	C16 H35 N O3	7.862
Unknown	C18 H29 F3 O4	7.905
Unknown	C15 H30 N2 O7 S	7.908
(9Z)-Tetradec-9-enoic acid	C14 H26 O2	7.95
3-dodecoxypropan-1-amine	C15 H33 N O	7.957
3-hydroxy-N-(1-hydroxy-4-methylpentan-2-yl)-5-oxo-6-phenylhexanamide	C18 H27 N O4	7.974
2-aminohexadecanoic acid	C16 H33 N O2	7.977
1-[2,6-Bis(4-ethylphenyl)-4,4a,8,8a-tetrahydro-[1,3]dioxino[5,4-d][1,3]dioxin-4-yl]ethane-1,2-diol	C24 H30 O6	8.068
(9Z,12Z,15Z)-N-(2-hydroxyethyl)octadeca-9,12,15-trienamide	C20 H35 N O2	8.097
Hexadecanamide	C16 H33 N O	8.113
(9Z,12Z,15Z)-N-(2-hydroxyethyl)octadeca-9,12,15-trienamide	C20 H35 N O2	8.21
N,N-Dimethyldodecan-1-amine N-oxide	C14 H31 N O	8.287
(4Z,6E,8R,10Z)-8-hydroxyhexadeca-4,6,10-trienoic acid	C16 H26 O3	8.383
Unknown	C21 H31 F O S	8.425
(11E,15Z)-9,10,13-trihydroxyoctadeca-11,15-dienoic acid	C18 H32 O5	8.502
(4E)-1-(4-Hydroxy-3-methoxyphenyl)dec-4-en-3-one	C17 H24 O3	8.555
4,4-Difluoropregn-5-ene-3,20-dione	C21 H28 F2 O2	8.602
Unknown	C18 H29 F3 O3	8.725
(2S,3R)-2-aminooctadecane-1,3-diol	C18 H39 N O2	8.831
8-(4-oxo-5-pent-2-enylcyclopent-2-en-1-yl)octanoic acid	C18 H28 O3	8.876
(2R,3S,4R,2'R,3'S,4'R)-5,5'-(Cyclododecylimino)di(1,2,3,4-pentanetetrol) (non-preferred name)	C22 H45 N O8	8.948
3,5-di-tert-butyl-4-hydroxybenzaldehyde	C15 H22 O2	8.956
8-(4-oxo-5-pent-2-enylcyclopent-2-en-1-yl)octanoic acid	C18 H28 O3	8.985
8-[(1S,5R)-4-oxo-5-[(Z)-pent-2-enyl]cyclopent-2-en-1-yl]octanoic acid	C18 H28 O3	8.987
Unknown	C39 H31 F2 N5 O2	9.051
Unknown	C33 H59 F O3 S	9.159
NP-020214	C18 H32 O3	9.162
Unknown	C32 H60 O7	9.259
Unknown	C32 H62 O7	9.317
Unknown	C18 H38 F N O	9.323
Unknown	C12 H27 F3 N4 S	9.337
7-[(2E)-3,7-Dimethyl-2,6-octadien-1-yl]-6-(methoxyamino)-9-methyl-9H-purin-7-ium	C17 H26 N5 O	9.341
(3bS,5aS,7R,8R,10aR,10bS)-7-(Hydroxymethyl)-10b-methyl-3b,4,5,6,7,8,9,10,10a,10b,11,12-dodecahydro-5a,8-methanocyclohepta[5,6]naphtho[2,1-b]furan-7-ol	C20 H28 O3	9.341
2-cyclohexylidenecyclohexan-1-one	C12 H18 O	9.342

N-{[(2R,4S,5R)-5-Vinyl-1-azabicyclo[2.2.2]oct-2-yl]methyl}cyclohexanecarboxamide	C17 H28 N2 O	9.342
(9Z)-Hexadec-9-enoic acid	C16 H30 O2	9.342
NP-014287	C18 H32 O3	9.345
N-methyl-N-octyloctan-1-amine	C17 H37 N	9.398
(9Z)-Hexadec-9-enoic acid	C16 H30 O2	9.405
(9Z,11E,13S)-13-hydroperoxyoctadeca-9,11-dienoic acid	C18 H32 O4	9.406
Unknown	C37 H36 F2 N3 O2 P	9.431
Unknown	C12 H31 Cl N6	9.489
(3bS,5aS,7R,8R,10aR,10bS)-7-(Hydroxymethyl)-10b-methyl-3b,4,5,6,7,8,9,10,10a,10b,11,12-dodecahydro-5a,8-methanocyclohepta[5,6]naphtho[2,1-b]furan-7-ol	C20 H28 O3	9.489
N-{[(2R,4S,5R)-5-Vinyl-1-azabicyclo[2.2.2]oct-2-yl]methyl}cyclohexanecarboxamide	C17 H28 N2 O	9.489
(9Z)-Hexadec-9-enoic acid	C16 H30 O2	9.489
Unknown	C32 H62 O7	9.498
Unknown	C25 H49 N5 O3	9.515
Unknown	C24 H54 F N5 O5	9.557
Unknown	C12 H31 Cl N6	9.615
[(1E,4E)-5,9-Dimethyl-1,4,8-decatrien-1-yl]benzene	C18 H24	9.618
(3bS,5aS,7R,8R,10aR,10bS)-7-(Hydroxymethyl)-10b-methyl-3b,4,5,6,7,8,9,10,10a,10b,11,12-dodecahydro-5a,8-methanocyclohepta[5,6]naphtho[2,1-b]furan-7-ol	C20 H28 O3	9.618
N-{[(2R,4S,5R)-5-Vinyl-1-azabicyclo[2.2.2]oct-2-yl]methyl}cyclohexanecarboxamide	C17 H28 N2 O	9.619
Hexadecanoic acid	C16 H30 O2	9.619
(9Z)-Hexadec-9-enoic acid	C16 H30 O2	9.619
(4E)-1-(4-Hydroxy-3-methoxyphenyl)dec-4-en-3-one	C17 H24 O3	9.658
9-{3-[(4-Cyclohexylbutanoyl)amino]-3-deoxy-beta-D-xylofuranosyl}-9H-purin-6-amine	C20 H30 N6 O4	9.66
(Z)-N-hydroxy-11-methyldodec-2-enamide	C13 H25 N O2	9.696
(9Z)-N-(2-Hydroxyethyl)octadec-9-enamide	C20 H39 N O2	9.696
(5Z,9Z,12Z)-Octadeca-5,9,12-trienoic acid ethyl ester	C20 H34 O2	9.698
(Z)-N-hydroxy-11-methyldodec-2-enamide	C13 H25 N O2	9.748
Unknown	C20 H36 F3 N O	9.828
N-(9-oxodecyl)acetamide	C12 H23 N O2	9.828
Octamethyl-1,3,5,7,2,4,6,8-tetroxatetrasilocane	C20 H39 N O2	9.829
(5Z,9Z,12Z)-Octadeca-5,9,12-trienoic acid ethyl ester	C20 H34 O2	9.833
N-[(2S,3R,4E)-1-{[(5R)-5-Ethyl-alpha-L-arabinopyranosyl]oxy}-3-hydroxy-4-octadecen-2-yl]heptanamide	C32 H61 N O7	9.916
5-(but-3-en-1-yl)-3-propyldihydrofuran-2(3H)-one	C11 H18 O2	9.921
Unknown	C20 H36 F3 N O	9.922
3-methyl-2-[(2Z)-pent-2-en-1-yl]cyclopent-2-en-1-one	C11 H16 O	9.922
(9Z)-N-(2-Hydroxyethyl)octadec-9-enamide	C20 H39 N O2	9.924
(5Z,9Z,12Z)-Octadeca-5,9,12-trienoic acid	C20 H34 O2	9.928
NP-014287	C18 H32 O3	9.962
1,2,3,4,5-Pentamethylbenzene	C18 H30 O2	9.963
4-[(2-Methyl-4-quinolinyl)methoxy]-N-({2,4,6-trioxo-5-[4-(3-pyridinylcarbonyl)-1-piperazinyl]hexahydro-5-pyrimidinyl}methyl)benzamide	C33 H31 N7 O6	10.008
4-[(1R,3S,5S,8R,9S,10R,11R,13R,14S,17R)-1,5,11,14-tetrahydroxy-10-(hydroxymethyl)-13-methyl-3-((2R,3R,4R,5R,6S)-3,4,5-trihydroxy-6-methyltetrahydro-2H- pyran-2-yloxy)hexadecahydro-1H-cyclopenta[a]phenanthren-17-yl]furan-2(5H)-one	C29 H44 O12	10.008
Unknown	C38 H35 F2 N O5	10.017

Unknown	C27 H50 F2 N3 O P	10.078
(2E,4E)-11-Methoxy-3,7,11-trimethyl-2,4-dodecadienamide	C16 H29 N O2	10.132
(5alpha,7alpha)-3-Amino-20-methylpregnan-7-ol	C22 H39 N O	10.134
N-(8Z,11Z,14Z-eicosatrienoyl)-ethanolamine	C22 H39 N O2	10.136
Unknown	C22 H38 F3 N O	10.14
(9Z,12Z)-N-(2-hydroxyethyl)octadeca-9,12-dienamide	C20 H37 N O2	10.163
2-{2-[2-(Dodecyloxy)ethoxy]ethoxy}ethanol	C18 H38 O4	10.201
(2E,4E)-N-Isobutyl-2,4-octadecadienamide	C22 H41 N O	10.258
Unknown	C19 H39 O4 P3	10.266
Unknown	C19 H30 F2	10.317
OPEO	C29 H44 O12	10.322
4-[(2-Methyl-4-quinolinyl)methoxy]-N-({2,4,6-trioxo-5-[4-(3-pyridinylcarbonyl)-1-piperazinyl]hexahydro-5-pyrimidinyl}methyl)benzamide	C33 H31 N7 O6	10.323
Unknown	C30 H59 N O P2	10.418
Unknown	C28 H53 N O2 S	10.428
methyl (1R,15S,17R,18R,19S,20S)-6,18-dimethoxy-17-(3,4,5-trimethoxybenzoyl)oxy-1,3,11,12,14,15,16,17,18,19,20,21-dodecahydroyohimban-19-carboxylate	C19 H36 O3	10.431
Unknown	C20 H46 F N5 O3	10.435
2-(dimethylamino)ethyl 3-cyclohexylpropanoate	C13 H25 N O2	10.449
Unknown	C19 H41 N	10.476
N,S-Ditrityl-L-cysteine	C41 H35 N O2 S	10.52
Unknown	C31 H38 F3 N2 O5 P	10.525
(4S,5S)-4-Methyl-5-undecyl-1,3-oxazolidin-2-one	C15 H29 N O2	10.546
1-Methyl-2-propylpiperidine	C9 H19 N	10.59
1,4-Cyclohexadiene	C6 H8	10.59
1,2,7,7-tetramethylbicyclo[2.2.1]hept-2-ene	C11 H18	10.59
N-{2-[2-(2-Aminoethoxy)ethoxy]ethyl}octadecanamide	C24 H50 N2 O3	10.59
(5alpha,7alpha)-3-Amino-20-methylpregnan-7-ol	C22 H39 N O	10.591
(9Z)-1-(4-Morpholinyl)-9-octadecen-1-one	C22 H41 N O2	10.591
1-Dodecyl-5-hexyl-1H-imidazol-2-amine	C21 H41 N3	10.591
[(E)-hept-1-enyl]benzene	C13 H18	10.591
1-Oxa-4-azacyclohexadecan-16-one	C14 H27 N O2	10.591
(3,4-dimethylphenyl)methanol	C9 H12 O	10.591
4,10-dihydroxy-10-methyl-dodecan-4-olide	C13 H24 O3	10.591
(E)-4-(2,6,6-trimethylcyclohexen-1-yl)but-3-en-2-one	C13 H20 O	10.591
Unknown	C16 H38 F N5 O	10.591
Unknown	C50 H84 F2 O	10.591
Cyclohexanecarboxamide, N-ethyl-5-methyl-2-(1-methylethyl)-	C13 H25 N O	10.591
2-(dimethylamino)ethyl 3-cyclohexylpropanoate	C13 H25 N O2	10.591
Ethyl (8Z,11Z,14Z)-icosa-8,11,14-trienoate	C22 H38 O2	10.591
[(3E)-6-Methyl-3-hepten-1-yl]benzene	C14 H20	10.592
1-(3,3,5-Trimethyl-1-cyclohexen-1-yl)-4-penten-1-one	C14 H22 O	10.592
1-ethenyl-2,4-dimethylbenzene	C10 H12	10.592
(9Z)-Hexadec-9-enoic acid	C18 H37 N O2	10.635
octadec-9-ynoic acid	C8 H24 O4 Si4	10.644
2,2,4,4,6,6,8,8,10,10,12,12-dodecamethyl-1,3,5,7,9,11-hexaoxa-2,4,6,8,10,12-hexasilacyclododecane	C12 H36 O6 Si6	10.648
2-cyclohexylidenecyclohexan-1-one	C12 H18 O	10.658
Unknown	C45 H87 F2 O4 P	10.658
(9Z)-1-(4-Morpholinyl)-9-octadecen-1-one	C22 H41 N O2	10.659
1,4-Cyclohexadiene	C6 H8	10.661
[(E)-hept-1-enyl]benzene	C13 H18	10.661

4,10-dihydroxy-10-methyl-dodecan-4-olide	C13 H24 O3	10.661
Unknown	C16 H38 F N5 O	10.661
Cyclohexene	C6 H10	10.661
(2E)-3,7-Dimethylocta-2,6-dien-1-yl acetate	C12 H20 O2	10.661
N-[1-(1,4'-Bipiperidin-1'-yl)-3-methyl-1-oxo-2-butanyl]-4-methylcyclohexanecarboxamide	C23 H41 N3 O2	10.661
N,N-bis(2-hydroxyethyl)hexadecanamide	C10 H14	10.661
1-Methyl-2-propylpiperidine	C9 H19 N	10.662
(5alpha,7alpha)-3-Amino-20-methylpregnan-7-ol	C22 H39 N O	10.662
(9Z)-N-[(3R)-2-Oxo-3-azepanyl]-9-octadecenamide	C24 H44 N2 O2	10.662
(Z)-N-hydroxy-11-methyldodec-2-enamide	C13 H25 N O2	10.662
1-Dodecyl-5-hexyl-1H-imidazol-2-amine	C21 H41 N3	10.662
1,2,7,7-tetramethylbicyclo[2.2.1]hept-2-ene	C11 H18	10.662
(E)-4-(2,6,6-trimethylcyclohexen-1-yl)but-3-en-2-one	C13 H20 O	10.662
Cyclohexanecarboxamide, N-ethyl-5-methyl-2-(1-methylethyl)-	C13 H25 N O	10.662
Ethyl (8Z,11Z,14Z)-icosa-8,11,14-trienoate	C22 H38 O2	10.662
N-(9-oxodecyl)acetamide	C12 H23 N O2	10.662
N-{2-[2-(2-Aminoethoxy)ethoxy]ethyl}octadecanamide	C24 H50 N2 O3	10.662
1-methyl-4-propan-2-ylbenzene	C11 H16	10.663
(1,3-dimethyl-4-phenylpiperidin-4-yl) propanoate	C16 H23 N O2	10.664
Unknown	C29 H38 F4 O9	10.712
N,S-Ditrityl-L-cysteine	C41 H35 N O2 S	10.712
Unknown	C21 H42 F N O	10.726
Unknown	C16 H38 F N5 O2	10.741
(9Z)-1-(4-Morpholinyl)-9-octadecen-1-one	C22 H41 N O2	10.743
N-(2-hydroxyethyl)-N-methyloctadecanamide	C21 H43 N O2	10.744
8-Bromo-2-(3-pyridinylcarbonyl)-1,3,4,12a-tetrahydropyrazino[2,1-c][1,4]benzodiazepine-6,12(2H,11H)-dione	C18 H15 Br N4 O3	10.747
Unknown	C19 H40 O4	10.786
N-[(E,2S,3R)-1,3-dihydroxyoctadec-4-en-2-yl]acetamide	C20 H39 N O3	10.802
(2R,3R,4S)-1-Butyl-2-{[(9Z)-9-octadecen-1-yloxy]methyl}-3,4-pyrrolidinediol	C27 H53 N O3	10.854
N-(2-Hydroxyethyl)-9-octadecenamide	C20 H39 N O2	10.863
cis-N-Butyl-4-(4-octylphenyl)cyclohexanamine	C24 H41 N	10.911
(9Z)-1-(4-Morpholinyl)-9-octadecen-1-one	C22 H41 N O2	10.981
Unknown	C31 H57 N O6	11.026
(9Z)-1-(4-Morpholinyl)-9-octadecen-1-one	C22 H41 N O2	11.076
(Z)-N-(2-hydroxyethyl)icos-11-enamide	C22 H43 N O2	11.129
methyl (1R,15S,17R,18R,19S,20S)-6,18-dimethoxy-17-(3,4,5-trimethoxybenzoyl)oxy-1,3,11,12,14,15,16,17,18,19,20,21-dodecahydroyohimban-19-carboxylate	C33 H40 N2 O9	11.164
Unknown	C24 H41 F2 N6 O4 P3	11.165
Unknown	C32 H57 F3 N3 O3 P3	11.194
Unknown	C23 H46 F5 N10 O P S	11.196
Unknown	C27 H38 F2 N10 O4 S	11.197
Unknown	C33 H53 N O8 P2	11.197
N-[2-(Diethylamino)ethyl]-2-{6-[2-(2,3-difluorophenyl)ethyl]-2,3-dimethyl-4-oxo-1(4H)-pyridinyl}-N-{[4'-(trifluoromethyl)-4-biphenylyl]methyl}acetamide	C37 H40 F5 N3 O2	11.197
NP-018660	C28 H46 N4 O11	11.197
hexadecyl(trimethyl)azanium	C19 H41 N	11.208
Unknown	C24 H46 N2 O2	11.286
N-[(Benzyloxy)carbonyl]-L-valyl-N-{(2S)-4-[(4-benzyl-5-oxo-2,5-dihydro-3-furanyl)oxy]-1-carboxy-3-oxo-2-butanyl}-L-alaninamide	C32 H37 N3 O10	11.292
(Z)-N-(2-hydroxyethyl)icos-11-enamide	C22 H43 N O2	11.298
Unknown	C30 H51 F O8 P2	11.33

Unknown	C21 H45 N3 O5	11.361
Unknown	C36 H77 N7 O P2	11.361
Unknown	C45 H70 O5	11.362
(4S,5S)-4-Methyl-5-undecyl-1,3-oxazolidin-2-one	C15 H29 N O2	11.373
(1S,4S,5aS,9aS)-1,7,7-Tributyl-4-(hydroxymethyl)-5a-methyldecahydro-2H-3-benzazepin-2-one	C24 H45 N O2	11.391
(2E,4E)-N-(2-methylpropyl)icosa-2,4-dienamide	C24 H45 N O	11.393
Unknown	C19 H41 F6 N6 O3 P3	11.431
(5Z,9Z,12Z)-Octadeca-5,9,12-trienoic acid ethyl ester	C33 H40 N2 O9	11.431
Unknown	C25 H29 Cl N9 P S	11.543
(Z)-N-hydroxy-11-methyldodec-2-enamide	C13 H25 N O2	11.58
1,1,6-Trimethyl-1,2-dihydronaphthalene	C13 H16	11.58
1,3-Di(propan-2-yl)benzene	C12 H18	11.58
[(E)-hept-1-enyl]benzene	C13 H18	11.58
5,5-Bis(methylperoxy)nonane	C11 H24 O4	11.58
(E)-4-(2,6,6-trimethylcyclohexen-1-yl)but-3-en-2-one	C13 H20 O	11.58
Unknown	C44 H85 F2 N4 O2 P	11.58
(1RS,6SR,10SR)-6,10-dimethylbicyclo[4.4.0]decan-3-one	C12 H20 O	11.581
12-Aminododecyl 2,6-diamino-2,6-dideoxy-beta-D-glucopyranoside	C18 H39 N3 O4	11.581
Unknown	C19 H41 N3 O6	11.581
exo-1,7,7-Trimethylbicyclo(2.2.1)hept-2-yl acrylate	C13 H20 O2	11.581
Azacyclotridecan-2-one	C12 H23 N O	11.581
N-[(2S)-4-[(5-Chloro-2-pyridinyl)amino]-1,4-dioxo-1-(1-piperidinyl)-2-butanyl]-2'-{[(2-methoxyethyl)(methyl)amino]methyl}-4-biphenylcarboxamide	C32 H38 Cl N5 O4	11.619
Unknown	C20 H44 N6 O14	11.62
Unknown	C15 H39 F4 N10 O2 P3 S	11.622
Methyl 3-({3-[4-(methoxycarbonyl)-4-phenyl-1-piperidinyl]propyl}carbamoyl)-6-methyl-4-(4-nitrophenyl)-2-oxo-1,2,3,4-tetrahydro-5-pyrimidinecarboxylate	C30 H35 N5 O8	11.622
2-[[(5S,6R,9S)-14-[[(2-fluoroanilino)-oxomethyl]amino]-5-methoxy-3,6,9-trimethyl-2-oxo-11-oxa-3,8-diazabicyclo[10.4.0]hexadeca-1(12),13,15-trien-8-yl]methyl]benzoic acid	C32 H37 F N4 O6	11.623
Unknown	C25 H42 F N S	11.717
(1RS,6SR,10SR)-6,10-dimethylbicyclo[4.4.0]decan-3-one	C12 H20 O	11.718
(2Z)-2-Hepten-1-ylbenzene	C13 H18	11.718
(Z)-N-hydroxy-11-methyldodec-2-enamide	C13 H25 N O2	11.718
1,3-Di(propan-2-yl)benzene	C12 H18	11.718
hept-2-ynylbenzene	C13 H16	11.718
5,5-Bis(methylperoxy)nonane	C11 H24 O4	11.718
(E)-4-(2,6,6-trimethylcyclohexen-1-yl)but-3-en-2-one	C13 H20 O	11.718
Unknown	C53 H83 F O2	11.718
exo-1,7,7-Trimethylbicyclo(2.2.1)hept-2-yl acrylate	C13 H20 O2	11.718
Azacyclotridecan-2-one	C12 H23 N O	11.718
(3R,4E)-5-(4-methoxy-5-methyl-6-oxopyran-2-yl)-3-methylhex-4-enoate	C13 H24 O4	11.718
Unknown	C29 H57 N O3	11.753
3-Ethyl-N-[(2S,3R)-3-hydroxy-1-phenyl-4-{[3-(trifluoromethyl)benzyl]amino}-2-butanyl]-7-(2-oxo-1-pyrrolidinyl)-1H-indole-5-carboxamide	C33 H35 F3 N4 O3	11.797
N-[(2S)-4-[(5-Chloro-2-pyridinyl)amino]-1,4-dioxo-1-(1-piperidinyl)-2-butanyl]-2'-{[(2-methoxyethyl)(methyl)amino]methyl}-4-biphenylcarboxamide	C32 H38 Cl N5 O4	11.797
Unknown	C4 H5 F2 N O5	11.865

methyl (1R,15S,17R,18R,19S,20S)-6,18-dimethoxy-17-(3,4,5-trimethoxybenzoyl)oxy-1,3,11,12,14,15,16,17,18,19,20,21-dodecahydroyohimban-19-carboxylate	C33 H40 N2 O9	11.914
N-[(1-{[(4S)-3-{[(2-Methyl-2-propanyl)oxy]carbonyl}-1,3-thiazolidin-4-yl]carbonyl}-4-phenyl-4-piperidinyl)carbonyl]-L-leucine	C27 H39 N3 O6 S	11.94
1'-ethyl-5-[2'-methyl-4'-(5-methyl-1,3,4-oxadiazolyl-2-yl)biphenyl-4-carbonyl]-2,3,6,7-tetrahydrospiro(furo[2,3-f]indole-3,4'-piperidine)	C33 H34 N4 O3	11.94
octadecanamide	C18 H37 N O	11.981
N-(2-Hydroxyethyl)octadecanamide	C20 H41 N O2	12.043
Unknown	C21 H45 N	12.113
(1R,9S)-5-[(E)-2-(4-Chlorophenyl)vinyl]-11-(5-pyrimidinylcarbonyl)-7,11-diazatricyclo[7.3.1.02,7]trideca-2,4-dien-6-one	C24 H21 Cl N4 O2	12.16
Unknown	C10 H23 F2 N6 O7 P S2	12.16
(2E,4E)-N-Isobutyl-2,4-octadecadienamide	C22 H41 N O	12.184
N-[(2S,3R)-1,3-dihydroxyoctadecan-2-yl]dodecanamide	C30 H61 N O3	12.247
Unknown	C39 H81 F N9 O P	12.264
Unknown	C42 H85 F N2 O P2 S	12.265
Unknown	C42 H27 Cl F N3	12.292
Unknown	C23 H49 N	12.305
Unknown	C37 H43 Cl F N S4	12.5
Unknown	C4 H5 F2 N O5	12.661
Unknown	C29 H37 F N6	12.743
N,N-Dimethyloctadecanamide	C20 H41 N O	12.782
2-Octadecylfuran	C22 H40 O	12.802
(Z)-docos-13-enamide	C22 H43 N O	12.806
Unknown	C30 H67 N6 O P	12.841
Unknown	C34 H68 N2 O2	12.847
Unknown	C39 H58 F2 O3	12.847
NP-008563	C18 H32 O3	12.892
Unknown	C36 H59 F3 O3	12.897
Unknown	C43 H35 Cl F2 N O2 P	12.902
Unknown	C41 H70 F2 N7 P	12.932
Unknown	C23 H49 N	12.974
N,N-Dimethyloctadecanamide	C20 H41 N O	13.102
Unknown	C25 H53 N	13.104
NP-014287	C18 H32 O3	13.173
Lanosta-8,24-dien-3beta,23-diol	C29 H48 O2	13.18
Unknown	C32 H60 O5	13.243
Unknown	C30 H55 F N4 O	13.244
OPEO	C16 H26 O2	13.244
Unknown	C30 H58 F N5 O2	13.312
Unknown	C33 H54 N4 O	13.312
Unknown	C31 H56 F N O S	13.316
(10E,12E)-9-oxooctadeca-10,12-dienoic acid	C18 H30 O3	13.353
Unknown	C53 H33 Cl F3 N	13.424
(9Z,11E,13S)-13-hydroperoxyoctadeca-9,11-dienoic acid	C18 H32 O4	13.45
(9Z)-Hexadec-9-enoic acid	C16 H30 O2	13.456
2-cyclohexylidenecyclohexan-1-one	C12 H18 O	13.457
Unknown	C30 H57 N O5	13.473
(4aS,5S,6S,8aS)-2-(6,10-Dimethyl-2-undecanyl)-5,8a-dimethyldecahydro-6-isoquinolinol	C24 H47 N O	13.483
Unknown	C30 H59 F3 O2	13.488
Unknown	C27 H59 N8 P	13.489
Unknown	C32 H46 F2	13.495

Docosanamide	C22 H45 N O	13.498
Unknown	C32 H60 O5	13.538
Unknown	C32 H63 N O5	13.539
(2S,3R,4E,8E)-1-(β-D-glucopyranosyl)-3-hydroxy-2-[(R)-2′-hydroxyhexadecanoyl]amino-9-methyl-4,8-heptadecadiene	C40 H75 N O9	13.578
Beyer-15-en-19-yl stearate	C38 H66 O2	13.661
(5S)-4-(Cyclohexylmethyl)-1-{4-[(2S)-1-(cyclohexylmethyl)-5,6-dioxo-2-piperazinyl]butyl}-5-isobutyl-2,3-piperazinedione	C30 H50 N4 O4	13.793
10-Oxo-10-[(3-oxoolean-12-en-28-yl)oxy]decanoic acid	C40 H64 O5	13.793
Unknown	C30 H58 O5 S	13.793
N-{(2S)-1-[3-(Adamantan-1-yl)-2,4-dioxo-1-imidazolidinyl]-6-amino-2-hexanyl}dodecanamide	C31 H54 N4 O3	13.793
Unknown	C5 H2 F O2 P	13.888
1-(9Z-hexadecenoyl)-2-(9Z,12Z-octadecadienoyl)-sn-glycerol	C37 H66 O5	13.942
N-Dodecyl-N-[2-({[(11beta,16alpha,17alpha)-9-fluoro-11,17-dihydroxy-16-methyl-3-oxoandrosta-1,4-dien-17-yl]carbonyl}amino)ethyl]-N-methyl-1-dodecanaminium	C48 H84 F N2 O4	13.951
Unknown	C5 H F2 O P	13.956
Unknown	C23 H30 Cl N7 O2 S2	13.965
Unknown	C21 H26 Cl N9 O2 S2	13.966
Unknown	C21 H31 N2 O2 P S4	13.966
Unknown	C21 H35 Cl F N7 S3	13.966
N-(1,3-benzodioxol-5-yl)-7-chloroquinolin-4-amine	C16 H11 Cl N2 O2	13.966
Unknown	C16 H33 Cl F4 N4 O3 S3	13.967
Unknown	C6 H12 F3 N2 O5 P	13.967

(c) ethanol extract.

Compound Name	Formula	RT [min]
1-Methylpyrrolidin-2-one	C5 H9 N O	0.517
Lanosta-8,24-dien-3beta,23-diol	C29 H48 O2	0.528
N,N-Dimethyldodecan-1-amine N-oxide	C25 H53 N	0.531
2-Hydroxy-N,N,N-trimethylethan-1-aminium	C5 H13 N O	0.584
2-Hydroxy-N,N,N-trimethylethan-1-aminium	C5 H13 N O	0.585
1,6-Dimethyl-1,6-Dinitroso-Biurea	C4 H8 N6 O4	0.595
1-Methylpyridin-1-ium-3-carboxylate	C7 H7 N O2	0.608
1,1-dimethylpyrrolidin-1-ium-2-carboxylic acid	C7 H13 N O2	0.624
2,2,6,6-Tetramethyl-1,4-piperidinediol	C9 H19 N O2	0.627
2-Methylpropanamine	C4 H11 N	0.627
4-Hydroxyquinoline-2-carboxylic acid	C10 H7 N O3	0.627
2-(methylamino)-1-phenylbutan-1-one	C11 H15 N O	0.628
(2R)-4-methyl-2-[[(2S)-1-phenylmethoxycarbonylpyrrolidine-2-carbonyl]amino]pentanoic acid	C11 H20 N2 O3	0.628
2,3-dihydroxybutanedihydrazide	C11 H15 N O2	0.629
2-Hydroxy-2-methylpropanenitrile	C4 H7 N O	0.63
9H-Purin-6-amine	C5 H5 N5	0.63
2-Hydroxy-2-methylpropanenitrile	C4 H7 N O	0.893
2,6-di-tert-butyl-4-ethylphenol	C4 H10 N4 O4	2.912
4-Hydroxyquinoline-2-carboxylic acid	C10 H7 N O3	3.196
Unknown	C13 H29 F O P2	3.627
3,6,9,12,15,18-Hexaoxaicosane-1,20-diol	C14 H30 O8	3.863
Unknown	C19 H35 F N3 O2 P	4.068
Unknown	C23 H45 N5 O8	4.549
Unknown	C25 H49 N5 O9	4.678

(2R,3S,4S,5S,6R)-2-({7-hydroxy-4-[(3Z)-5-hydroxy-3-methylpent-3-en-1-yl]-4a,8,8-trimethyl-3-methylidene-decahydronaphthalen-2-yl}oxy)-6-(hydroxymethyl)oxane-3,4,5-triol	C26 H44 O8	4.824
(2R,3S,4S,5S,6R)-2-({7-hydroxy-4-[(3Z)-5-hydroxy-3-methylpent-3-en-1-yl]-4a,8,8-trimethyl-3-methylidene-decahydronaphthalen-2-yl}oxy)-6-(hydroxymethyl)oxane-3,4,5-triol	C26 H44 O8	4.898
Unknown	C26 H58 F N5 O12	4.901
(9*E*,12*Z*)-10-nitrooctadeca-9,12-dienoic acid	C18 H31 N O4	6.281
NP-018660	C13 H22 O3	6.41
(11E,15Z)-9,10,13-trihydroxyoctadeca-11,15-dienoic acid	C18 H32 O5	6.521
NP-017152	C13 H23 N O2	6.66
3-(1′-Hydroxypentyl)phenol	C11 H16 O2	6.773
chromen-2-one	C9 H6 O2	6.806
(9Z,11E,13S)-13-hydroperoxyoctadeca-9,11-dienoic acid	C18 H32 O4	6.841
(9Z)-Hexadec-9-enoic acid	C16 H30 O2	6.841
3-Methyl-1-phenyl-1,2-nonadiene	C16 H22	6.875
1-phenyldecan-1-one	C16 H24 O	6.875
2-Aminohexadecanoic acid	C19 H24 N4	6.876
OPEO	C16 H26 O2	6.876
(4Z,6E,8R,10Z)-8-hydroxyhexadeca-4,6,10-trienoic acid	C16 H26 O3	6.887
NP-020214	C16 H32 O4	6.985
2-[(3S)-1-(Cyclohexylmethyl)-3-pyrrolidinyl]-1H-benzimidazole-5-carbonitrile	C16 H26 O	6.986
6-cyclohexyl-5-(cyclohexylimino)-4-thia-6-azaspiro[2.4]heptan-7-one	C17 H26 N2 O S	7.011
NP-008563	C16 H28 O4	7.017
(4Z,6E,8R,10Z)-8-hydroxyhexadeca-4,6,10-trienoic acid	C16 H26 O3	7.018
1-[(2-Hydroxyethyl)amino]-2-dodecanol	C14 H31 N O2	7.021
N,N-Dimethyldodecan-1-amine N-oxide	C12 H27 N O	7.055
(2S,3S,4R)-2-aminotetradecane-1,3,4-triol	C14 H31 N O3	7.16
OPEO	C16 H26 O2	7.19
2-(dimethylamino)ethyl 3-cyclohexylpropanoate	C13 H25 N O2	7.236
exo-1,7,7-Trimethylbicyclo(2.2.1)hept-2-yl acrylate	C13 H20 O2	7.381
(2Z,4S,5S,6S,7R,8Z)-2,9-Diphenyldeca-2,8-diene-3,4,5,6,7,8-hexol	C22 H26 O6	7.809
(4Z,6E,8S,10Z)-8-hydroxyhexadeca-4,6,10-trienoic acid	C16 H26 O3	7.817
Apigenin 7- (3'',6''-di-p-coumarylglucoside)	C39 H32 O14	7.967
6-O-[(2E)-3-(4-Hydroxyphenyl)-2-propenoyl]-1-O-[(2E)-3-phenyl-2-propenoyl]-β-D-glucopyranose	C24 H24 O9	7.968
chromen-2-one	C9 H6 O2	7.969
chromen-2-one	C9 H6 O2	8.017
6-O-[(2E)-3-(4-Hydroxyphenyl)-2-propenoyl]-1-O-[(2E)-3-phenyl-2-propenoyl]-β-D-glucopyranose	C24 H24 O9	8.018
chromen-2-one	C9 H6 O2	8.068
6-O-[(2E)-3-(4-Hydroxyphenyl)-2-propenoyl]-1-O-[(2E)-3-phenyl-2-propenoyl]-β-D-glucopyranose	C24 H24 O9	8.133
chromen-2-one	C9 H6 O2	8.133
Hexadecanoic acid	C16 H32 O2	8.21
N,N-Dimethyldodecan-1-amine N-oxide	C14 H31 N O	8.254
(2S,3S,4R)-2-Aminooctadecane-1,3,4-triol	C18 H39 N O3	8.273
Hexadecanoic acid	C16 H32 O2	8.315
N,N-bis(2-hydroxyethyl)dodecan-1-amine oxide	C16 H35 N O3	8.317
1-Methylamino-1-(3,4-Methylenedioxyphenyl)Propane	C16 H33 N O2	8.439
Unknown	C32 H45 F O2 P2	8.478
1-[2,6-Bis(4-ethylphenyl)-4,4a,8,8a-tetrahydro-[1,3]dioxino[5,4-d][1,3]dioxin-4-yl]ethane-1,2-diol	C24 H30 O6	8.513

Hexadecanamide	C16 H33 N O	8.584
N,N-Dimethyldodecan-1-amine N-oxide	C14 H31 N O	8.771
Unknown	C36 H76 F2 N3 O7 P	8.835
N-(3-Methylbutanoyl)-D-valyl-N-[(3S)-1-{[(2R)-1-{[(2R)-1,2-dihydroxy-5-methyl-3-hexanyl]amino}-1-oxo-2-propanyl]amino}-3-hydroxy-6-methyl-1-oxo-4-heptanyl]-D-valinamide	C33 H63 N5 O8	9.077
(9Z)-Hexadec-9-enoic acid	C16 H30 O2	9.142
(9Z)-Hexadec-9-enoic acid	C16 H30 O2	9.214
Unknown	C39 H74 F3 N9 O4	9.242
(2S,3R)-2-aminooctadecane-1,3-diol	C18 H39 N O2	9.356
NP-004713	C15 H24 O2	9.37
Unknown	C32 H58 O7	9.625
(9Z,11E,13E)-Octadeca-9,11,13-trienoic acid	C18 H30 O2	9.66
Unknown	C32 H58 O7	9.698
(9Z,11E,13E)-Octadeca-9,11,13-trienoic acid	C18 H30 O2	9.719
2-cyclohexylidenecyclohexan-1-one	C12 H18 O	9.879
N-{[(2R,4S,5R)-5-Vinyl-1-azabicyclo[2.2.2]oct-2-yl]methyl}cyclohexanecarboxamide	C17 H28 N2 O	9.879
(9Z)-Hexadec-9-enoic acid	C16 H30 O2	9.879
(3bS,5aS,7R,8R,10aR,10bS)-7-(Hydroxymethyl)-10b-methyl-3b,4,5,6,7,8,9,10,10a,10b,11,12-dodecahydro-5a,8-methanocyclohepta[5,6]naphtho[2,1-b]furan-7-ol	C20 H28 O3	9.88
(9Z,11E,13S)-13-hydroperoxyoctadeca-9,11-dienoic acid	C18 H32 O4	9.951
(9Z)-Hexadec-9-enoic acid	C16 H30 O2	9.951
3-Hydroxy-2,2-bis[(nonanoyloxy)methyl]propyl nonanoate	C32 H60 O7	9.985
N-{[(2R,4S,5R)-5-Vinyl-1-azabicyclo[2.2.2]oct-2-yl]methyl}cyclohexanecarboxamide	C17 H28 N2 O	10.036
(3bS,5aS,7R,8R,10aR,10bS)-7-(Hydroxymethyl)-10b-methyl-3b,4,5,6,7,8,9,10,10a,10b,11,12-dodecahydro-5a,8-methanocyclohepta[5,6]naphtho[2,1-b]furan-7-ol	C20 H28 O3	10.039
(9Z)-Hexadec-9-enoic acid	C16 H30 O2	10.039
(10E,12E)-9-oxooctadeca-10,12-dienoic acid	C18 H30 O3	10.18
N-{[(2R,4S,5R)-5-Vinyl-1-azabicyclo[2.2.2]oct-2-yl]methyl}cyclohexanecarboxamide	C17 H28 N2 O	10.18
(9Z)-Hexadec-9-enoic acid	C16 H30 O2	10.18
(4E)-1-(4-Hydroxy-3-methoxyphenyl)dec-4-en-3-one	C17 H24 O3	10.23
(9Z,12Z)-N-(2-hydroxyethyl)octadeca-9,12-dienamide	C20 H37 N O2	10.763
1-(3-cyano-3,3-diphenylpropyl)-4-phenylpiperidine-4-carboxylic acid	C28 H28 N2 O2	10.877
3-[(3R,12Z,21S,22S)-16-ethenyl-11-ethyl-12-(hydroxymethylidene)-3-methoxycarbonyl-17,21,26-trimethyl-4-oxo-7,23,24,25-tetrazahexacyclo[18.2.1.15,8.110,13.115,18.02,6]hexacosa-1,5(26),6,8,10,13(25),14,16,18(24),19-decaen-22-yl]propanoic acid	C35 H34 N4 O6	11.168
3,5-dichloro-2'-O-methylnorstenosporic acid	C23 H26 Cl2 O7	11.295
Unknown	C13 H12 Cl2 N8 O4	11.399
cis-N-Butyl-4-(4-octylphenyl)cyclohexanamine	C24 H41 N	11.42
3-(4-tert-Butylphenyl)-1-(4-methoxyphenyl)propane-1,3-dione	C20 H22 O3	11.516
(9Z)-N-(2-Hydroxyethyl)octadec-9-enamide	C20 H39 N O2	11.523
cis-N-Butyl-4-(4-octylphenyl)cyclohexanamine	C24 H41 N	11.625
Unknown	C26 H36 F2 N8 O3 P2	11.861
methyl (1R,15S,17R,18R,19S,20S)-6,18-dimethoxy-17-(3,4,5-trimethoxybenzoyl)oxy-1,3,11,12,14,15,16,17,18,19,20,21-dodecahydroyohimban-19-carboxylate	C33 H40 N2 O9	11.862
5-hydroxy-3-methyl-N-{3-[(2R,5R)-5-{3-[(2E)-3-methyl-5-{[(2S,3R,4R,5S,6S)-3,4,5-trihydroxy-6-(hydroxymethyl)oxan-2-yl]oxy}pent-2-enamido]propyl}-3,6-dioxopiperazin-2-yl]propyl}pent-2-enamide	C28 H46 N4 O11	11.911

Unknown	C28 H52 F6 N3 O P3	11.912
methyl (1R,15S,17R,18R,19S,20S)-6,18-dimethoxy-17-(3,4,5-trimethoxybenzoyl)oxy-1,3,11,12,14,15,16,17,18,19,20,21-dodecahydroyohimban-19-carboxylate	C33 H40 N2 O9	12.152
Unknown	C32 H35 F2 N4 O4 P	12.153
Decamethylcyclopentasiloxane	C10 H30 O5 Si5	12.275
N-[(2R)-2,3-Dihydroxypropyl]-5-fluoro-2-[(2-{[4-(4-isopropyl-1-piperazinyl)-2-methoxyphenyl]amino}-1H-pyrrolo[2,3-d]pyrimidin-4-yl)amino]benzamide	C30 H37 F N8 O4	12.362
Unknown	C38 H35 F3 N2 O	12.363
Methyl 3-({3-[4-(methoxycarbonyl)-4-phenyl-1-piperidinyl]propyl}carbamoyl)-6-methyl-4-(4-nitrophenyl)-2-oxo-1,2,3,4-tetrahydro-5-pyrimidinecarboxylate	C30 H35 N5 O8	12.363
3-[(3S,4S,21R)-14-Ethyl-21-(methoxycarbonyl)-4,8,13,18-tetramethyl-20-oxo-9-vinyl-3-phorbinyl]propanoic acid	C35 H36 N4 O5	12.55
Unknown	C20 H46 F2 N10 O2 S3	12.55
1'-ethyl-5-[2'-methyl-4'-(5-methyl-1,3,4-oxadiazolyl-2-yl)biphenyl-4-carbonyl]-2,3,6,7-tetrahydrospiro(furo[2,3-f]indole-3,4'-piperidine)	C33 H34 N4 O3	12.706
(1R,9S)-5-[(E)-2-(4-Chlorophenyl)vinyl]-11-(5-pyrimidinylcarbonyl)-7,11-diazatricyclo[7.3.1.02,7]trideca-2,4-dien-6-one	C24 H21 Cl N4 O2	12.949
1-isothiocyanatooctane	C9 H17 N S	12.949
N-[(2S,3R)-1,3-dihydroxyoctadecan-2-yl]dodecanamide	C30 H61 N O3	13.035
Decamethylcyclopentasiloxane	C10 H30 O5 Si5	13.092
Unknown	C27 H47 Cl N O3 P S4	13.093
Unknown	C37 H28 Cl N5 S	13.469
(Z)-docos-13-enamide	C22 H43 N O	13.637
Unknown	C28 H48 Cl N3 O7 S4	13.758
Unknown	C39 H80 F3 N O P2 S	13.781

(d) ethyl acetate extract.

Compound Name	Formula	RT [min]
Unknown	C26 H49 F O	0.511
4,6-dimethylpyrimidin-2-amine	C6 H9 N3	0.525
N,N-Dimethyldodecan-1-amine N-oxide	C25 H53 N	0.529
Unknown	C6 H11 F3 O4	0.594
Unknown	C9 H17 F3 N4	0.626
11-Aminoundecanoic acid	C11 H23 N O2	0.627
1-Methylamino-1-(3,4-Methylenedioxyphenyl)Propane	C11 H15 N O2	0.627
2-Amino-6-({[(2-chlorobenzyl)oxy]carbonyl}amino)hexanoic acid	C14 H19 Cl N2 O4	0.627
Unknown	C12 H25 F2 N2 O P	0.627
2-Hydroxy-2-methylpropanenitrile	C4 H7 N O	0.628
pyridine-3-carboxamide	C6 H6 N2 O	0.628
2-Ethyl-2-oxazoline	C5 H9 N O	0.634
2-Hydroxy-2-methylpropanenitrile	C4 H7 N O	0.788
2-Hydroxy-2-methylpropanenitrile	C4 H7 N O	0.887
3,6,9,12,15-Pentaoxaheptadecan-1,17-diol	C12 H26 O7	3.631
3,6,9,12,15,18-Hexaoxaicosane-1,20-diol	C14 H30 O8	3.867
Unknown	C17 H33 N5 O5	4.072
Unknown	C22 H48 F2 N3 O6 P	4.551
Unknown	C25 H49 N5 O9	4.68
NP-018660	C13 H22 O3	6.41
3-(1′-Hydroxypentyl)phenol	C11 H16 O2	6.774
chromen-2-one	C9 H6 O2	6.806
(12Z)-9,10,11-trihydroxyoctadec-12-enoic acid	C18 H34 O5	6.831
Unknown	C16 H24 F4 O2	6.874

1-phenyldecan-1-one	C16 H24 O	6.874
1-Ethynyl-4-octylbenzene	C16 H22	6.875
OPEO	C16 H26 O2	6.875
6-cyclohexyl-5-(cyclohexylimino)-4-thia-6-azaspiro[2.4]heptan-7-one	C17 H26 N2 O S	7.012
NP-008563	C16 H28 O4	7.017
1-[(2-Hydroxyethyl)amino]-2-dodecanol	C14 H31 N O2	7.021
N,N-Dimethyldodecan-1-amine N-oxide	C12 H27 N O	7.055
2,2,6,6-Tetramethyl-4-piperidinol	C9 H19 N O	7.07
(2S,3S,4R)-2-aminotetradecane-1,3,4-triol	C14 H31 N O3	7.162
OPEO	C16 H26 O2	7.19
2-(dimethylamino)ethyl 3-cyclohexylpropanoate	C13 H25 N O2	7.24
exo-1,7,7-Trimethylbicyclo(2.2.1)hept-2-yl acrylate	C13 H20 O2	7.381
4-oxododecanedioic acid	C12 H20 O5	7.809
(2Z,4S,5S,6S,7R,8Z)-2,9-Diphenyldeca-2,8-diene-3,4,5,6,7,8-hexol	C22 H26 O6	7.809
Ethenylbenzene	C8 H8	7.809
3-(4-hydroxy-3-methoxyphenyl)-N-{4-[3-(4-hydroxy-3-methoxyphenyl)propanamido]butyl}propanamide	C24 H32 N2 O6	7.81
6-O-[(2E)-3-(4-Hydroxyphenyl)-2-propenoyl]-1-O-[(2E)-3-phenyl-2-propenoyl]-β-D-glucopyranose	C24 H24 O9	7.967
chromen-2-one	C9 H6 O2	7.967
6-O-[(2E)-3-(4-Hydroxyphenyl)-2-propenoyl]-1-O-[(2E)-3-phenyl-2-propenoyl]-β-D-glucopyranose	C24 H24 O9	8.015
(11E,15Z)-9,10,13-trihydroxyoctadeca-11,15-dienoic acid	C18 H32 O5	8.018
chromen-2-one	C9 H6 O2	8.018
chromen-2-one	C9 H6 O2	8.067
6-O-[(2E)-3-(4-Hydroxyphenyl)-2-propenoyl]-1-O-[(2E)-3-phenyl-2-propenoyl]-β-D-glucopyranose	C24 H24 O9	8.133
chromen-2-one	C9 H6 O2	8.135
Hexadecanoic acid	C16 H32 O2	8.208
N,N-Dimethyldodecan-1-amine N-oxide	C14 H31 N O	8.256
(2S,3S,4R)-2-Aminooctadecane-1,3,4-triol	C18 H39 N O3	8.275
N,N-bis(2-hydroxyethyl)dodecan-1-amine oxide	C16 H35 N O3	8.317
2-aminohexadecanoic acid	C16 H33 N O2	8.442
Ethyl hydrogen [(1s,4s)-dispiro[cyclohexane-1,3'-[1,2,4]trioxolane-5',2''-tricyclo[3.3.1.1~3,7~]decan]-4-ylmethyl]phosphonate	C19 H31 O6 P	8.465
methyl (4aS,4bR,5aR,6aS,7R,9aS,9bR,10R)-4a,6a-dimethyl-2,5'-dioxo-2,4,4',4a,5',5a,6,6a,8,9,9a,9b,10,11-tetradecahydro-3H,3'H-spiro[cyclopenta[7,8]phenanthro[4b,5-b]oxirene-7,2'-furan]-10-carboxylate	C24 H30 O6	8.512
Hexadecanamide	C16 H33 N O	8.584
N,N-Dimethyldodecan-1-amine N-oxide	C14 H31 N O	8.774
(9Z)-Hexadec-9-enoic acid	C16 H30 O2	9.143
(9Z)-Hexadec-9-enoic acid	C16 H30 O2	9.214
(2S,3R)-2-aminooctadecane-1,3-diol	C18 H39 N O2	9.359
NP-004713	C15 H24 O2	9.37
Unknown	C32 H58 O7	9.625
(9Z,12Z,15Z)-Octadeca-9,12,15-trienoic acid	C18 H30 O2	9.66
Unknown	C33 H56 F2 O4	9.699
NP-020521	C18 H32 O3	9.718
2-cyclohexylidenecyclohexan-1-one	C12 H18 O	9.879
(9Z)-Hexadec-9-enoic acid	C16 H30 O2	9.879
(9Z,11E,13S)-13-hydroperoxyoctadeca-9,11-dienoic acid	C18 H32 O4	9.88
(3bS,5aS,7R,8R,10aR,10bS)-7-(Hydroxymethyl)-10b-methyl-3b,4,5,6,7,8,9,10,10a,10b,11,12-dodecahydro-5a,8-methanocyclohepta[5,6]naphtho[2,1-b]furan-7-ol	C20 H28 O3	9.88

(9Z,11E,13S)-13-hydroperoxyoctadeca-9,11-dienoic acid	C18 H32 O4	9.951
(9Z)-Hexadec-9-enoic acid	C16 H30 O2	9.951
N-{[(2R,4S,5R)-5-Vinyl-1-azabicyclo[2.2.2]oct-2-yl]methyl}cyclohexanecarboxamide	C17 H28 N2 O	10.038
(9Z)-Hexadec-9-enoic acid	C16 H30 O2	10.038
(3bS,5aS,7R,8R,10aR,10bS)-7-(Hydroxymethyl)-10b-methyl-3b,4,5,6,7,8,9,10,10a,10b,11,12-dodecahydro-5a,8-methanocyclohepta[5,6]naphtho[2,1-b]furan-7-ol	C20 H28 O3	10.039
N-{[(2R,4S,5R)-5-Vinyl-1-azabicyclo[2.2.2]oct-2-yl]methyl}cyclohexanecarboxamide	C17 H28 N2 O	10.177
(10E,12E)-9-oxooctadeca-10,12-dienoic acid	C18 H30 O3	10.179
(9Z)-Hexadec-9-enoic acid	C16 H30 O2	10.18
(9Z,12Z)-N-(2-hydroxyethyl)octadeca-9,12-dienamide	C20 H37 N O2	10.762
Unknown	C17 H32 N2 O10	10.878
3-[(3R,12Z,21S,22S)-16-ethenyl-11-ethyl-12-(hydroxymethylidene)-3-methoxycarbonyl-17,21,26-trimethyl-4-oxo-7,23,24,25-tetrazahexacyclo[18.2.1.15,8.110,13.115,18.02,6]hexacosa-1,5(26),6,8,10,13(25),14,16,18(24),19-decaen-22-yl]propanoic acid	C35 H34 N4 O6	11.168
3,5-dichloro-2'-O-methylnorstenosporic acid	C23 H26 Cl2 O7	11.295
Unknown	C13 H12 Cl2 N8 O4	11.4
(9Z)-N-(2-Hydroxyethyl)octadec-9-enamide	C20 H39 N O2	11.523
Unknown	C24 H41 F2 N6 O4 P3	11.86
methyl (1R,15S,17R,18R,19S,20S)-6,18-dimethoxy-17-(3,4,5-trimethoxybenzoyl)oxy-1,3,11,12,14,15,16,17,18,19,20,21-dodecahydroyohimban-19-carboxylate	C33 H40 N2 O9	11.861
5-hydroxy-3-methyl-N-{3-[(2R,5R)-5-{3-[(2E)-3-methyl-5-{[(2S,3R,4R,5S,6S)-3,4,5-trihydroxy-6-(hydroxymethyl)oxan-2-yl]oxy}pent-2-enamido]propyl}-3,6-dioxopiperazin-2-yl]propyl}pent-2-enamide	C28 H46 N4 O11	11.91
Unknown	C32 H49 F N3 O8 P	11.911
Unknown	C36 H55 O11 P	11.911
methyl (1R,15S,17R,18R,19S,20S)-6,18-dimethoxy-17-(3,4,5-trimethoxybenzoyl)oxy-1,3,11,12,14,15,16,17,18,19,20,21-dodecahydroyohimban-19-carboxylate	C33 H40 N2 O9	12.152
Unknown	C19 H41 F6 N6 O3 P3	12.154
Decamethylcyclopentasiloxane	C10 H30 O5 Si5	12.275
Unknown	C21 H39 F6 N10 O P	12.363
2-(Allyl{4-[(2-ethyl-5,7-dimethyl-3H-imidazo[4,5-b]pyridin-3-yl)methyl]phenyl}amino)-2-phenyl-N-(phenylsulfonyl)acetamide	C34 H35 N5 O3 S	12.367
3-[(3S,4S,21R)-14-Ethyl-21-(methoxycarbonyl)-4,8,13,18-tetramethyl-20-oxo-9-vinyl-3-phorbinyl]propanoic acid	C35 H36 N4 O5	12.367
Unknown	C20 H44 N6 O14	12.549
Unknown	C35 H36 N4 O5	12.549
1'-ethyl-5-[2'-methyl-4'-(5-methyl-1,3,4-oxadiazolyl-2-yl)biphenyl-4-carbonyl]-2,3,6,7-tetrahydrospiro(furo[2,3-f]indole-3,4'-piperidine)	C33 H34 N4 O3	12.704
(1R,9S)-5-[(E)-2-(4-Chlorophenyl)vinyl]-11-(5-pyrimidinylcarbonyl)-7,11-diazatricyclo[7.3.1.02,7]trideca-2,4-dien-6-one	C24 H21 Cl N4 O2	12.948
1-isothiocyanatooctane	C9 H17 N S	12.948
Decamethylcyclopentasiloxane	C10 H30 O5 Si5	13.092
N-{4-[4-(4-Chlorophenyl)-1-piperazinyl]benzyl}-2-(dibenzo[b,d]thiophen-2-yl)imidazo[1,2-a]pyridine-3-carboxamide	C37 H30 Cl N5 O S	13.092
(Z)-docos-13-enamide	C22 H43 N O	13.639
Unknown	C42 H31 Cl F3 N3 O2	13.76

(e) methanol extract.

Compound Name	Formula	RT [min]
5,6-epoxy-24(R)-methylcholesta-7,22-dien-3β-ol	C28 H46 O2	0.514

Unknown	C27 H51 N O3	0.521
2,6-di-tert-butyl-4-ethylphenol	C9 H12 O	0.525
2-Amino-6-({[(2-chlorobenzyl)oxy]carbonyl}amino)hexanoic acid	C6 H9 N3	0.525
Lanosta-8,24-dien-3beta,23-diol	C29 H48 O2	0.526
NP-021293	C30 H48 O4	0.526
N,N-Dimethyldodecan-1-amine N-oxide	C25 H53 N	0.529
2-Hydroxy-N,N,N-trimethylethan-1-aminium	C5 H13 N O	0.583
2-Hydroxy-N,N,N-trimethylethan-1-aminium	C5 H13 N O	0.591
Unknown	C4 H10 F2 N2 O5	0.594
1,6-Dimethyl-1,6-Dinitroso-Biurea	C4 H8 N6 O4	0.596
1-Methylpyridin-1-ium-3-carboxylate	C7 H7 N O2	0.607
1-Methylpyridin-1-ium-3-carboxylate	C7 H7 N O2	0.607
1,1-dimethylpyrrolidin-1-ium-2-carboxylic acid	C7 H13 N O2	0.623
3-(1-hydroxyethyl)-2,3,6,7,8,8a-hexahydropyrrolo[1,2-a]pyrazine-1,4-dione	C9 H14 N2 O3	0.626
4-Hydroxyquinoline-2-carboxylic acid	C10 H7 N O3	0.626
(2R)-4-methyl-2-[[(2S)-1-phenylmethoxycarbonylpyrrolidine-2-carbonyl]amino]pentanoic acid	C11 H20 N2 O3	0.626
2,2,6,6-Tetramethyl-1,4-piperidinediol	C9 H19 N O2	0.627
2-(Allyl{4-[(2-ethyl-5,7-dimethyl-3H-imidazo[4,5-b]pyridin-3-yl)methyl]phenyl}amino)-2-phenyl-N-(phenylsulfonyl)acetamide	C11 H15 N O2	0.627
2-Ethyl-2-oxazoline	C5 H9 N O	0.627
2-Hydroxy-2-methylpropanenitrile	C4 H7 N O	0.627
2-(methylamino)-1-phenylbutan-1-one	C11 H15 N O	0.627
N-(tert-Butyl)-N'-(2-hydroxyethyl)urea	C7 H16 N2 O2	0.627
9*H*-Purin-6-amine	C5 H5 N5	0.628
2-Aminohexadecanoic acid	C14 H19 Cl N2 O4	0.633
2-Hydroxy-2-methylpropanenitrile	C4 H7 N O	0.895
2,3-dihydroxybutanedihydrazide	C4 H10 N4 O4	2.958
4-Hydroxyquinoline-2-carboxylic acid	C10 H7 N O3	3.216
3,6,9,12,15,18-Hexaoxaicosane-1,20-diol	C14 H30 O8	3.847
Unknown	C20 H39 N O2 P2	4.057
Unknown	C23 H45 N5 O8	4.542
Unknown	C25 H49 N5 O9	4.671
(2R,3S,4S,5S,6R)-2-({7-hydroxy-4-[(3Z)-5-hydroxy-3-methylpent-3-en-1-yl]-4a,8,8-trimethyl-3-methylidene-decahydronaphthalen-2-yl}oxy)-6-(hydroxymethyl)oxane-3,4,5-triol	C26 H44 O8	4.801
(2R,3S,4S,5S,6R)-2-({7-hydroxy-4-[(3Z)-5-hydroxy-3-methylpent-3-en-1-yl]-4a,8,8-trimethyl-3-methylidene-decahydronaphthalen-2-yl}oxy)-6-(hydroxymethyl)oxane-3,4,5-triol	C26 H44 O8	4.895
(9*E*,12*Z*)-10-nitrooctadeca-9,12-dienoic acid	C18 H31 N O4	6.279
NP-018660	C13 H22 O3	6.408
N-Dodecyl-3,4,5-trihydroxybenzamide	C19 H31 N O4	6.411
8-(4-oxo-5-pent-2-enylcyclopent-2-en-1-yl)octanoic acid	C18 H28 O3	6.518
(11E,15Z)-9,10,13-trihydroxyoctadeca-11,15-dienoic acid	C18 H32 O5	6.519
NP-017152	C13 H23 N O2	6.658
3-Butyl-3a,4,5,6-tetrahydro-2-benzofuran-1(3H)-one	C12 H18 O2	6.834
Unknown	C17 H36 F O P3	6.835
(9Z)-Hexadec-9-enoic acid	C16 H30 O2	6.838
(9Z,11E,13S)-13-hydroperoxyoctadeca-9,11-dienoic acid	C18 H32 O4	6.839
Hexadecanedioic acid	C16 H30 O4	6.859
1-phenyldecan-1-one	C16 H24 O	6.873
(S)-1-[3-(Dimethylamino)propyl]-1-(4-fluorophenyl)-1,3-dihydroisobenzofuran-5-carbonitrile	C20 H21 F N2 O	6.873
OPEO	C16 H26 O2	6.873

Hexadecanedioic acid	C16 H30 O4	6.873
3-Methyl-1-phenyl-1,2-nonadiene	C16 H22	6.874
Hexadecanedioic acid	C16 H30 O4	6.897
2-Amino-4,6-dimethylpyrimidine	C16 H26 O	6.985
6-cyclohexyl-5-(cyclohexylimino)-4-thia-6-azaspiro[2.4]heptan-7-one	C17 H26 N2 O S	7.001
NP-008563	C16 H28 O4	7.016
(4Z,6E,8R,10Z)-8-hydroxyhexadeca-4,6,10-trienoic acid	C16 H26 O3	7.016
1-[(2-Hydroxyethyl)amino]-2-dodecanol	C14 H31 N O2	7.022
N,N-Dimethyldodecan-1-amine N-oxide	C12 H27 N O	7.054
2,3,6-Trimethylphenol	C9 H19 N O	7.067
(2S,3S,4R)-2-aminotetradecane-1,3,4-triol	C14 H31 N O3	7.161
OPEO	C16 H26 O2	7.189
Hexadecanedioic acid	C16 H30 O4	7.189
Hexadecanedioic acid	C16 H30 O4	7.189
2-(dimethylamino)ethyl 3-cyclohexylpropanoate	C13 H25 N O2	7.241
exo-1,7,7-Trimethylbicyclo(2.2.1)hept-2-yl acrylate	C13 H20 O2	7.379
(2Z,4S,5S,6S,7R,8Z)-2,9-Diphenyldeca-2,8-diene-3,4,5,6,7,8-hexol	C22 H26 O6	7.808
(4Z,6E,8S,10Z)-8-hydroxyhexadeca-4,6,10-trienoic acid	C16 H26 O3	7.816
6-O-[(2E)-3-(4-Hydroxyphenyl)-2-propenoyl]-1-O-[(2E)-3-phenyl-2-propenoyl]-β-D-glucopyranose	C24 H24 O9	7.966
chromen-2-one	C9 H6 O2	7.966
chromen-2-one	C9 H6 O2	8.015
6-O-[(2E)-3-(4-Hydroxyphenyl)-2-propenoyl]-1-O-[(2E)-3-phenyl-2-propenoyl]-β-D-glucopyranose	C24 H24 O9	8.131
chromen-2-one	C9 H6 O2	8.131
Hexadecanoic acid	C16 H32 O2	8.206
N,N-Dimethyldodecan-1-amine N-oxide	C14 H31 N O	8.252
(2S,3S,4R)-2-Aminooctadecane-1,3,4-triol	C18 H39 N O3	8.273
N,N-bis(2-hydroxyethyl)dodecan-1-amine oxide	C16 H35 N O3	8.315
1-Methylamino-1-(3,4-Methylenedioxyphenyl)Propane	C16 H33 N O2	8.437
methyl (4aS,4bR,5aR,6aS,7R,9aS,9bR,10R)-4a,6a-dimethyl-2,5'-dioxo-2,4,4',4a,5',5a,6,6a,8,9,9a,9b,10,11-tetradecahydro-3H,3'H-spiro[cyclopenta[7,8]phenanthro[4b,5-b]oxirene-7,2'-furan]-10-carboxylate	C24 H30 O6	8.512
Hexadecanamide	C16 H33 N O	8.582
N,N-Dimethyldodecan-1-amine N-oxide	C14 H31 N O	8.77
(9Z)-Hexadec-9-enoic acid	C16 H30 O2	9.142
(9Z)-Hexadec-9-enoic acid	C16 H30 O2	9.213
(2S,3R)-2-aminooctadecane-1,3-diol	C18 H39 N O2	9.358
NP-004713	C15 H24 O2	9.368
Unknown	C31 H53 F3 N4 O	9.625
(9Z,11E,13E)-Octadeca-9,11,13-trienoic acid	C18 H30 O2	9.659
Unknown	C33 H56 F2 O4	9.697
(9Z,12Z,15Z)-Octadeca-9,12,15-trienoic acid	C18 H30 O2	9.714
N-{[(2R,4S,5R)-5-Vinyl-1-azabicyclo[2.2.2]oct-2-yl]methyl}cyclohexanecarboxamide	C17 H28 N2 O	9.877
2-cyclohexylidenecyclohexan-1-one	C12 H18 O	9.878
(9Z)-Hexadec-9-enoic acid	C16 H30 O2	9.878
(3bS,5aS,7R,8R,10aR,10bS)-7-(Hydroxymethyl)-10b-methyl-3b,4,5,6,7,8,9,10,10a,10b,11,12-dodecahydro-5a,8-methanocyclohepta[5,6]naphtho[2,1-b]furan-7-ol	C20 H28 O3	9.879
(9Z)-Hexadec-9-enoic acid	C16 H30 O2	9.949
(9Z,11E,13S)-13-hydroperoxyoctadeca-9,11-dienoic acid	C18 H32 O4	9.95

(3bS,5aS,7R,8R,10aR,10bS)-7-(Hydroxymethyl)-10b-methyl-3b,4,5,6,7,8,9,10,10a,10b,11,12-dodecahydro-5a,8-methanocyclohepta[5,6]naphtho[2,1-b]furan-7-ol	C20 H28 O3	10.037
N-{[(2R,4S,5R)-5-Vinyl-1-azabicyclo[2.2.2]oct-2-yl]methyl}cyclohexanecarboxamide	C17 H28 N2 O	10.037
(9Z)-Hexadec-9-enoic acid	C16 H30 O2	10.037
N-{[(2R,4S,5R)-5-Vinyl-1-azabicyclo[2.2.2]oct-2-yl]methyl}cyclohexanecarboxamide	C17 H28 N2 O	10.178
(9Z)-Hexadec-9-enoic acid	C16 H30 O2	10.178
(10E,12E)-9-oxooctadeca-10,12-dienoic acid	C18 H30 O3	10.179
(9Z,12Z)-N-(2-hydroxyethyl)octadeca-9,12-dienamide	C20 H37 N O2	10.761
Unknown	C20 H31 F3 O6	10.875
3-[(3R,12Z,21S,22S)-16-ethenyl-11-ethyl-12-(hydroxymethylidene)-3-methoxycarbonyl-17,21,26-trimethyl-4-oxo-7,23,24,25-tetrazahexacyclo[18.2.1.15,8.110,13.115,18.02,6]hexacosa-1,5(26),6,8,10,13(25),14,16,18(24),19-decaen-22-yl]propanoic acid	C35 H34 N4 O6	11.167
(4R,4'S)-2'-(3-Chlorophenyl)-5'-phenyl-4'-(2-thienyl)-2,2',3,4'-tetrahydro-5H-spiro[1-benzothiepine-4,3'-pyrazol]-5-one	C28 H21 Cl N2 O S2	11.293
2,2,4,4,6,6,8,8,10,10,12,12-dodecamethyl-1,3,5,7,9,11-hexaoxa-2,4,6,8,10,12-hexasilacyclododecane	C12 H36 O6 Si6	11.293
Decamethylcyclopentasiloxane	C10 H30 O5 Si5	11.294
8-Bromo-2-(3-pyridinylcarbonyl)-1,3,4,12a-tetrahydropyrazino[2,1-c][1,4]benzodiazepine-6,12(2H,11H)-dione	C18 H15 Br N4 O3	11.398
Unknown	C21 H42 F N O	11.42
(9Z)-N-(2-Hydroxyethyl)octadec-9-enamide	C20 H39 N O2	11.521
Unknown	C21 H42 F N O	11.623
methyl (1R,15S,17R,18R,19S,20S)-6,18-dimethoxy-17-(3,4,5-trimethoxybenzoyl)oxy-1,3,11,12,14,15,16,17,18,19,20,21-dodecahydroyohimban-19-carboxylate	C33 H40 N2 O9	11.859
Unknown	C33 H53 N O8 P2	11.908
methyl (1R,15S,17R,18R,19S,20S)-6,18-dimethoxy-17-(3,4,5-trimethoxybenzoyl)oxy-1,3,11,12,14,15,16,17,18,19,20,21-dodecahydroyohimban-19-carboxylate	C33 H40 N2 O9	12.151
5'-O-({1-[(2e)-4-(4-Hydroxy-6-Methoxy-7-Methyl-3-Oxo-1,3-Dihydro-2-Benzofuran-5-Yl)-2-Methylbut-2-En-1-Yl]-1h-1,2,3-Triazol-4-Yl}methyl)adenosine	C28 H32 N8 O8	12.154
Unknown	C31 H33 Cl F N O S2	12.273
2,2,6,6-Tetramethyl-4-piperidinol	C34 H35 N5 O3 S	12.36
3-[(3S,4S,21R)-14-Ethyl-21-(methoxycarbonyl)-4,8,13,18-tetramethyl-20-oxo-9-vinyl-3-phorbinyl]propanoic acid (Pheophorbide A)	C35 H36 N4 O5	12.36
Unknown	C20 H42 F4 N10 O2 P2	12.36
3-[(3S,4S,21R)-14-Ethyl-21-(methoxycarbonyl)-4,8,13,18-tetramethyl-20-oxo-9-vinyl-3-phorbinyl]propanoic acid	C35 H36 N4 O5	12.548
Unknown	C20 H44 N6 O14	12.549
1'-ethyl-5-[2'-methyl-4'-(5-methyl-1,3,4-oxadiazolyl-2-yl)biphenyl-4-carbonyl]-2,3,6,7-tetrahydrospiro(furo[2,3-f]indole-3,4'-piperidine)	C33 H34 N4 O3	12.702
(1R,9S)-5-[(E)-2-(4-Chlorophenyl)vinyl]-11-(5-pyrimidinylcarbonyl)-7,11-diazatricyclo[7.3.1.02,7]trideca-2,4-dien-6-one	C24 H21 Cl N4 O2	12.946
1-isothiocyanatooctane	C9 H17 N S	12.947
N-[(2S,3R)-1,3-dihydroxyoctadecan-2-yl]dodecanamide	C30 H61 N O3	13.034
Decamethylcyclopentasiloxane	C10 H30 O5 Si5	13.091
N-{4-[4-(4-Chlorophenyl)-1-piperazinyl]benzyl}-2-(dibenzo[b,d]thiophen-2-yl)imidazo[1,2-a]pyridine-3-carboxamide	C37 H30 Cl N5 O S	13.091
(Z)-docos-13-enamide	C22 H43 N O	13.638
Unknown	C28 H48 Cl N3 O7 S4	13.759

Table S2. Common and unique compounds identified in different *E. foeminea* crude extracts via Venn diagram analysis using positive ion mode LC–MS.

Occurrence	Total	Compound Name
ACE DCM/MeOH EA EtOH MeOH	29	2-Aminohexadecanoic Acid
		1-Phenyldecan-1-one
		(1R,9S)-5-[(E)-2-(4-Chlorophenyl)Vinyl]-11-(5-Pyrimidinylcarbonyl)-7,11-Diazatricyclo[7.3.1.02,7]Trideca-2,4-Dien-6-one
		(2S,3S,4R)-2-Aminotetradecane-1,3,4-triol
		(2S,3R)-2-Aminooctadecane-1,3-diol
		(10E,12E)-9-Oxooctadeca-10,12-Dienoic Acid
		Hexadecanamide
		(9Z,11E,13S)-13-Hydroperoxyoctadeca-9,11-Dienoic Acid
		NP-018660
		(11E,15Z)-9,10,13-Trihydroxyoctadeca-11,15-Dienoic Acid
		(9Z,12Z)-N-(2-Hydroxyethyl)Octadeca-9,12-Dienamide
		Hexadecanoic Acid
		N-{[(2R,4S,5R)-5-Vinyl-1-Azabicyclo[2.2.2]Oct-2-yl]Methyl}Cyclohexanecarboxamide
		Methyl (1R,15S,17R,18R,19S,20S)-6,18-Dimethoxy-17-(3,4,5-Trimethoxybenzoyl)Oxy-1,3,11,12,14,15,16,17,18,19,20,21-Dodecahydroyohimban-19-Carboxylate
		6-Cyclohexyl-5-(Cyclohexylimino)-4-Thia-6-Azaspiro[2.4]Heptan-7-one
		(Z)-Docos-13-enamide
		Exo-1,7,7-Trimethylbicyclo(2.2.1)Hept-2-yl Acrylate
		N,N-Dimethyldodecan-1-Amine N-Oxide
		1'-Ethyl-5-[2'-Methyl-4'-(5-Methyl-1,3,4-Oxadiazolyl-2-yl)Biphenyl-4-Carbonyl]-2,3,6,7-Tetrahydrospiro(Furo[2,3-F]Indole-3,4'-Piperidine)
		(3BS,5AS,7R,8R,10AR,10BS)-7-(Hydroxymethyl)-10b-Methyl-3b,4,5,6,7,8,9,10,10a,10b,11,12-Dodecahydro-5a,8-Methanocyclohepta[5,6]Naphtho[2,1-B]Furan-7-ol
		Chromen-2-one
		(2Z,4S,5S,6S,7R,8Z)-2,9-Diphenyldeca-2,8-Diene-3,4,5,6,7,8-hexol
		(9Z)-Hexadec-9-Enoic Acid
		OPEO
		6-O-[(2E)-3-(4-Hydroxyphenyl)-2-Propenoyl]-1-O-[(2E)-3-Phenyl-2-Propenoyl]-Î’-D-Glucopyranose
		(9Z)-N-(2-Hydroxyethyl)Octadec-9-enamide
		1-[(2-Hydroxyethyl)Amino]-2-Dodecanol
		2-(Dimethylamino)Ethyl 3-Cyclohexylpropanoate
		NP-008563
DCM/MeOH EA EtOH MeOH	1	2-Cyclohexylidenecyclohexan-1-one
ACE DCM/MeOH EtOH MeOH	7	2,3-Dihydroxybutanedihydrazide
		Lanosta-8,24-Dien-3beta,23-diol
		4-Hydroxyquinoline-2-Carboxylic Acid
		(4Z,6E,8R,10Z)-8-Hydroxyhexadeca-4,6,10-Trienoic Acid
		2,6-Di-Tert-Butyl-4-Ethylphenol
		N-[(2S,3R)-1,3-Dihydroxyoctadecan-2-yl]Dodecanamide
		(2R,3S,4S,5S,6R)-2-({7-Hydroxy-4-[(3z)-5-Hydroxy-3-Methylpent-3-en-1-yl]-4a,8,8-Trimethyl-3-Methylidene-Decahydronaphthalen-2-yl}Oxy)-6-(Hydroxymethyl)Oxane-3,4,5-triol

ACE DCM/MeOH EA MeOH	1	C20 H44 N6 O13
ACE DCM/MeOH EA EtOH	1	5-hydroxy-3-methyl-N-{3-[(2R,5R)-5-{3-[(2E)-3-methyl-5-{[(2S,3R,4R,5S,6S)-3,4,5-trihydroxy-6-(hydroxymethyl)oxan-2-yl]oxy}pent-2-enamido]propyl}-3,6-dioxopiperazin-2-yl]propyl}pent-2-enamide
ACE EA EtOH MeOH	11	1-Isothiocyanatooctane
		C25 H49 N5 O8
		3-[(3S,4S,21R)-14-Ethyl-21-(methoxycarbonyl)-4,8,13,18-tetramethyl-20-oxo-9-vinyl-3-phorbinyl]propanoic acid
		2-Hydroxy-2-methylpropanenitrile
		3,6,9,12,15,18-Hexaoxaicosane-1,20-diol
		1-Methylamino-1-(3,4-Methylenedioxyphenyl)Propane
		Decamethylcyclopentasiloxane
		(2S,3S,4R)-2-Aminooctadecane-1,3,4-triol
		N,N-Bis(2-Hydroxyethyl)Dodecan-1-Amine Oxide
		NP-004713
		3-[(3R,21S,22S)-16-ethenyl-11-ethyl-12-(hydroxymethylidene)-3-methoxycarbonyl-17,21,26-trimethyl-4-oxo-7,23,24,25-tetrazahexacyclo[18.2.1.15,8.110,13.115,18.02,6]hexacosa-1,5(26),6,8,10,13(25),14,16,18(24),19-decaen-22-yl]propanoic acid
ACE DCM/MeOH MeOH	1	C21 H42 F N O
ACE DCM/MeOH EtOH	2	Cis-N-Butyl-4-(4-Octylphenyl)Cyclohexanamine
		NP-020214
ACE DCM/MeOH EA	2	Ethenylbenzene
		C24 H41 F2 N6 O4 P2
ACE EtOH MeOH	8	2-(Methylamino)-1-Phenylbutan-1-one
		C23 H45 N5 O7
		2-Hydroxy-N,N,N-Trimethylethan-1-Aminium
		9H-Purin-6-Amine
		(4Z,6E,8S,10Z)-8-hydroxyhexadeca-4,6,10-trienoic acid
		3-Methyl-1-Phenyl-1,2-Nonadiene
		(9E,11Z,13E)-octadeca-9,11,13-trienoic acid
		NP-017152
ACE EA MeOH	5	2-Ethyl-2-Oxazoline
		Methyl (4aS,4bS,5aS,6aS,7R,9aS,9bR,10R)-4a,6a-dimethyl-2,5′-dioxo-2,4,4a,4′,5a,5′,6,6a,8,9,9a,9b,10,11-tetradecahydro-3H,3′H-spiro[cyclopenta[1,2]phenanthro[4,4a-b]oxirene-7,2′-furan]-10-carboxylate
		2-(Allyl{4-[(2-Ethyl-5,7-Dimethyl-3H-Imidazo[4,5-B]Pyridin-3-yl)Methyl]Phenyl}Amino)-2-Phenyl-N-(Phenylsulfonyl)Acetamide
		2-Amino-6-({[(2-Chlorobenzyl)Oxy]Carbonyl}Amino)Hexanoic Acid
		N-{4-[4-(4-Chlorophenyl)-1-Piperazinyl]Benzyl}-2-(Dibenzo[B,D]Thiophen-2-yl)Imidazo[1,2-A]Pyridine-3-Carboxamide
ACE EA EtOH	1	3-(2-Hydroxypentyl)phenol
DCM/MeOH MeOH	6	2,2,4,4,6,6,8,8,10,10,12,12-Dodecamethyl-1,3,5,7,9,11-Hexaoxa-2,4,6,8,10,12-Hexasilacyclododecane
		3-Butyl-3a,4,5,6-Tetrahydro-2-Benzofuran-1(3H)-one
		8-(4-Oxo-5-Pent-2-Enylcyclopent-2-en-1-yl)Octanoic Acid
		C33 H53 N O8 P1
		8-Bromo-2-(3-pyridinylcarbonyl)-1,3,4,12a-tetrahydropyrazino[2,1-c][1,4]benzodiazepine-6,12(2H,11H)-dione
		(S)-1-[3-(Dimethylamino)Propyl]-1-(4-Fluorophenyl)-1,3-Dihydroisobenzofuran-5-Carbonitrile

DCM/MeOH EtOH	3	1-[2,6-Bis(4-Ethylphenyl)-4,4a,8,8a-Tetrahydro-[1,3]Dioxino[5,4-D][1,3]Dioxin-4-yl]Ethane-1,2-diol
		(4E)-1-(4-Hydroxy-3-methoxyphenyl)dec-4-en-3-one
		Methyl 3-({3-[4-(Methoxycarbonyl)-4-Phenyl-1-Piperidinyl]Propyl}Carbamoyl)-6-Methyl-4-(4-Nitrophenyl)-2-Oxo-1,2,3,4-Tetrahydro-5-Pyrimidinecarboxylate
DCM/MeOH EA	4	C17 H33 N5 O4
		C19 H41 F6 N6 O3 P2
		NP-020521
		(12Z)-9,10,11-trihydroxyoctadec-12-enoic acid
ACE DCM/MeOH	8	N,N-Bis(2-Hydroxyethyl)Hexadecanamide
		(3E,4S)-3-[2-[(1S,4aS,5R,6R,8aS)-6-hydroxy-5-(hydroxymethyl)-5,8a-dimethyl-2-methylidene-3,4,4a,6,7,8-hexahydro-1H-naphthalen-1-yl]ethylidene]-4-hydroxyoxolan-2-one
		NP-014287
		C13 H25 F N8 O4 P1
		4-((1R,3S,5S,8R,10R,11R,13R,14S,17R)-1,5,11,14-tetrahydroxy-10-(hydroxymethyl)-13-methyl-3-((2R,3R,4R,5R,6S)-3,4,5-trihydroxy-6-methyl-tetrahydro-2H-pyran-2-yloxy)-hexadecahydro-1H-cyclopenta[a]phenanthren-17-yl)furan-2(5H)-one
		2-[1-(2H-1,3-benzodioxol-5-yl)propan-2-yl]-6-methoxy-4-(prop-2-en-1-yl)phenol
		NP-020014
		C32 H62 O6
EtOH MeOH	7	1,1-Dimethylpyrrolidin-1-ium-2-Carboxylic Acid
		(9E,12Z)-10-Nitrooctadeca-9,12-dienoic acid
		2,2,6,6-Tetramethyl-1,4-Piperidinediol
		(2R)-4-methyl-2-[[(2S)-1-phenylmethoxycarbonylpyrrolidine-2-carbonyl]amino]pentanoic acid
		1,6-Dimethyl-1,6-Dinitroso-Biurea
		1-Methylpyridin-1-Ium-3-Carboxylate
		C28 H48 Cl N3 O7 S3
EA MeOH	3	(9Z,12Z,15Z)-octadeca-9,12,15-trienoic acid
		2,2,6,6-Tetramethyl-4-Piperidinol
		C33 H56 F2 O3
ACE MeOH	5	C4 H10 F2 N2 O4
		NP-021293
		C20 H31 F3 O5
		C31 H53 F3 N4 O
		5'-O-({1-[(2E)-4-(4-Hydroxy-6-Methoxy-7-Methyl-3-Oxo-1,3-Dihydro-2-Benzofuran-5-yl)-2-Methylbut-2-en-1-yl]-1h-1,2,3-Triazol-4-yl}Methyl)Adenosine
EA EtOH	3	C32 H58 O6
		3,5-Dichloro-2'-O-Methylnorstenosporic Acid
		C13 H12 Cl2 N8 O3
ACE EtOH	5	C32 H45 F O2 P1
		1-Methylpyrrolidin-2-one
		C19 H35 F N3 O2 P
		2-[(3S)-1-(Cyclohexylmethyl)-3-pyrrolidinyl]-1H-benzimidazole-5-carbonitrile
		Apigenin 7- (3'',6''-Di-P-Coumarylglucoside)
ACE EA	6	C32 H49 F N3 O8 P
		C26 H49 F O
		4,6-Dimethylpyrimidin-2-Amine
		C9 H17 F3 N3
		Pyridine-3-Carboxamide
		3,6,9,12,15-Pentaoxaheptadecan-1,17-diol

DCM/MeOH	225	5,5-Bis(Methylperoxy)Nonane
		C32 H63 N O4
		3-Ethyl-N-[(2S,3R)-3-Hydroxy-1-Phenyl-4-{[3-(Trifluoromethyl)Benzyl]Amino}-2-Butanyl]-7-(2-Oxo-1-Pyrrolidinyl)-1h-Indole-5-Carboxamide
		(9aR,9bS)-9a-Hydroxy-6,9-dimethyl-3-methylene-3,3a,4,5,9a,9b-hexahydroazuleno[4,5-b]furan-2,7-dione
		C38 H35 F2 N O4
		C33 H59 F O3 S
		4-Methylpyridine-2,3-Diamine
		(3,4-Dimethylphenyl)Methanol
		C23 H30 Cl N7 O2 S1
		4,4-Difluoropregn-5-Ene-3,20-dione
		2-Aminoethyl (Allylamino)Methanedithioate Hydrochloride
		(4aR,9aS)-7-Ethynyl-4a,9a-Dimethyl-1-Methylene-2,3,4,4a,5,6,9,9a-Octahydro-1H-Benzo[7]Annulene
		1-{[4-(2,5-Dimethyl-1h-Pyrrol-1-yl)Benzoyl]Oxy}Dihydro-1h-Pyrrole-2,5-dione
		Azacyclotridecan-2-one
		C45 H70 O4
		3-Hydroxy-N-(1-Hydroxy-4-Methylpentan-2-yl)-5-Oxo-6-Phenylhexanamide
		C53 H33 Cl F3 N
		(1S,4S,5aS,9aS)-1,7,7-Tributyl-4-(Hydroxymethyl)-5a-Methyldecahydro-2H-3-Benzazepin-2-one
		4,10-Dihydroxy-10-Methyl-Dodecan-4-olide
		C21 H26 Cl N9 O2 S1
		C44 H85 F2 N4 O2 P
		C19 H40 O3
		N-(9-Oxodecyl)Acetamide
		(Z)-N-Hydroxy-11-Methyldodec-2-enamide
		C30 H59 N O P1
		C28 H62 F N5 O7
		N-{2-[2-(2-Aminoethoxy)Ethoxy]Ethyl}Octadecanamide
		C37 H36 F2 N3 O2 P
		C27 H59 N8 P
		C3 H F4 P
		4-Isopropylbenzaldehyde
		(E)-4-(2,6,6-Trimethylcyclohexen-1-yl)But-3-en-2-one
		C21 H45 N
		N-[(Benzyloxy)Carbonyl]-L-Valyl-N-{(2s)-4-[(4-Benzyl-5-Oxo-2,5-Dihydro-3-Furanyl)Oxy]-1-Carboxy-3-Oxo-2-Butanyl}-L-Alaninamide
		C22 H38 F3 N O
		3-[2-Carboxyethyl(Dodecyl)Amino]Propanoic Acid
		Hexadecyl(Trimethyl)Azanium
		C28 H53 N O2 S
		3,5,7-Trimethyl-1-Azatricyclo[3.3.1.1~3,7~]Decane-2,8-diol
		(2E,4E)-N-(2-methylpropyl)icosa-2,4-dienamide
		(2Z)-2-Hepten-1-ylbenzene
		(2R,3R,4S)-1-Butyl-2-{[(9Z)-9-Octadecen-1-yloxy]Methyl}-3,4-Pyrrolidinediol
		C38 H71 N O5
		C41 H70 F2 N7 P
		N-(8Z,11Z,14Z-eicosatrienoyl)-ethanolamine

C51 H80 F2 O

(4aS,5S,6S,8aS)-2-(6,10-dimethyl-2-undecanyl)-5,8a-dimethyldecahydro-6-isoquinolinol

C18 H29 F3 O2

(1RS,6SR,10sSR)-6,10-dimethylbicyclo[4.4.0]decan-3-one

C43 H35 Cl F2 N O2 P

C31 H31 Cl F N S1

(1S,2S,4R,8S,9S,11S,12R,13S,19S)-12,19-difluoro-11-hydroxy-8-(2-hydroxyacetyl)-6,6,9,13-tetramethyl-5,7-dioxapentacyclo[10.8.0.02,9.04,8.013,18]icosa-14,17-dien-16-one

(5alpha,7alpha)-3-Amino-20-Methylpregnan-7-ol

C18 H29 F3 O3

1-Phenylethanone

(2E)-3,7-Dimethylocta-2,6-Dien-1-yl Acetate

N-{(2S)-1-[3-(adamantan-1-yl)-2,4-dioxo-1-imidazolidinyl]-6-amino-2-hexanyl}dodecanamide

(2R,3S,4R,2′R,3′S,4′R)-5,5′-(Cyclododecylimino)Di(1,2,3,4-Pentanetetrol)

C23 H49 N

1-Methyl-2-Propylpiperidine

C24 H54 F N5 O4

C20 H36 F3 N O

C20 H46 F N5 O2

C30 H57 N O4

5,7-Diisopropyl-1,1-Dimethyl-1,2,3,4-Tetrahydronaphthalene

1-Methyl-4-Propan-2-ylbenzene

C3 H10 F2 N3 O2 P

C25 H53 F3 N4 O3

Ethyl (8Z,11Z,14Z)-8,11,14-icosatrienoate

C16 H38 F N5 O

C21 H31 F O S

(1R,4aS,10aS)-7-(2-hydroxypropan-2-yl)-1,4a-dimethyl-9-oxo-3,4,10,10a-tetrahydro-2H-phenanthrene-1-carboxylic acid

C4 H5 F2 N O4

[(1E,4E)-5,9-Dimethyl-1,4,8-Decatrien-1-yl]Benzene

N,N-Dimethyloctadecanamide

Octamethyl-1,3,5,7,2,4,6,8-Tetroxatetrasilocane

C32 H60 O6

1,2,3,4,5-Pentamethylbenzene

C10 H23 F N8 O

N-[(2s,3s)-3-Fluoro-1-Hydroxy-2-Dodecanyl]Hexanamide

3-{(4r,7s,8as)-7-[(Isopropylcarbamoyl)Amino]-1-Oxooctahydropyrrolo[1,2-A]Pyrazin-4-yl}-N-(4-Methoxybenzyl)Propanamide

(Rs)-1-[2-(Allyloxy)Phenoxy]-3-(Isopropylamino)Propan-2-ol

C27 H38 F2 N10 O4 S

C21 H37 F4 N O8

C27 H50 F2 N3 O P

N-[(1-{[(4s)-3-{[(2-Methyl-2-Propanyl)Oxy]Carbonyl}-1,3-Thiazolidin-4-yl]Carbonyl}-4-Phenyl-4-Piperidinyl)Carbonyl]-L-Leucine

(6s)-6-[(1z)-But-1-en-1-yl]Cyclohepta-1,4-Diene

N-(1,3-Benzodioxol-5-yl)-7-Chloroquinolin-4-Amine

1-Benzyl-4-Propylamino-4-Piperidinecarboxamide

N,N-Bis(2-Hydroxyethyl)Dodecanamide

C12 H31 Cl N5

1,2,7,7-Tetramethylbicyclo[2.2.1]Hept-2-Ene

C22 H28 F5 N4

3,5-Di-Tert-Butyl-4-Hydroxybenzaldehyde

3-Dodecoxypropan-1-Amine

C21 H35 Cl F N7 S2

C31 H38 F3 N2 O5 P

C25 H42 F N S

C16 (2S,3S,4R)-2-Aminooctadecane-1,3,4-triol

C30 H67 N6 O P

C29 H38 F4 O8

(3r,4e)-5-(4-Methoxy-5-Methyl-6-Oxopyran-2-yl)-3-Methylhex-4-Enoate

N-(2-Hydroxyethyl)-9-Octadecenamide

1,1,6-Trimethyl-1,2-Dihydronaphthalene

C18 H38 F N O

(5z,9Z,12Z)-Octadeca-5,9,12-Trienoic Acid

1-Hydroxy-2,2,6,6-Tetramethylpiperidin-4-ol

(2S, 3R, 4E, 8E)1-(β-D-glucopyranosyl)-3-hydroxy-2-[(R)-2'-hydroxyhexadecanoyl]amino-9-methyl-4, 8-heptadecadiene

5-(But-3-en-1-yl)-3-Propyldihydrofuran-2(3H)-one

C5 H F2 O P

Cyclohexene

(E)-3-(2-Hydroxyphenyl)Prop-2-Enoic Acid

C15 H39 F4 N10 O2 P3 S

C45 H87 F2 O4 P

(9Z)-1-(4-Morpholinyl)-9-Octadecen-1-one

C32 H46 F1

C30 H55 F N4 O

1-Oxa-4-Azacyclohexadecan-16-one

C23 H46 F5 N10 O P S

C15 H30 N2 O7 S

C15 H34 N2 O1

Octadec-9-Ynoic Acid

2-[[(5S,6R,9S)-14-[[(2-Fluoroanilino)-Oxomethyl]Amino]-5-Methoxy-3,6,9-Trimethyl-2-Oxo-11-Oxa-3,8-Diazabicyclo[10.4.0]Hexadeca-1(12),13,15-Trien-8-yl]Methyl]Benzoic Acid

8-[(1S,5R)-4-Oxo-5-[(Z)-pent-2-enyl]cyclopent-2-en-1-yl]octanoic acid

(2E,4E)-11-methoxy-3,7,11-trimethyl-2,4-dodecadienamide

(1,3-Dimethyl-4-Phenylpiperidin-4-yl) Propanoate

1-(3,3,5-Trimethyl-1-Cyclohexen-1-yl)-4-Penten-1-one

C12 H12 Cl F N10 S1

C31 H56 F N O S

1,3-Di(Propan-2-yl)Benzene

C25 H49 N5 O2

C30 H58 F N5 O1

[(3E)-6-Methyl-3-Hepten-1-yl]Benzene

C39 H31 F2 N5 O1

C5 H2 F O2 P

C6 H12 F3 N2 O5 P

C42 H27 Cl F N2

C53 H83 F O1

1-(9Z-Hexadecenoyl)-2-(9Z,12Z-Octadecadienoyl)-Sn-Glycerol

C39 H81 F N9 O P

N-Dodecyl-N-[2-({[(11beta,16alpha,17alpha)-9-Fluoro-11,17-Dihydroxy-16-Methyl-3-Oxoandrosta-1,4-Dien-17-yl]Carbonyl}Amino)Ethyl]-N-Methyl-1-Dodecanaminium

C39 H58 F2 O2

(4S,5S)-4-Methyl-5-Undecyl-1,3-Oxazolidin-2-one

1-Dodecyl-5-Hexyl-1h-Imidazol-2-Amine

1-Ethenyl-2,4-Dimethylbenzene

2-Octadecylfuran

N-[2-(Diethylamino)Ethyl]-2-{6-[2-(2,3-Difluorophenyl)Ethyl]-2,3-Dimethyl-4-Oxo-1(4H)-Pyridinyl}-N-{[4'-(Trifluoromethyl)-4-Biphenylyl]Methyl}Acetamide

12-Aminododecyl 2,6-Diamino-2,6-Dideoxy-Beta-D-Glucopyranoside

N-[(2S,3R,4E)-1-{[(5R)-5-Ethyl-Alpha-L-Arabinopyranosyl]Oxy}-3-Hydroxy-4-Octadecen-2-yl]Heptanamide

Docosanamide

2-Acetamido-3-Sulfanylpropanoic Acid

C21 H31 N2 O2 P S3

Hept-2-Ynylbenzene

2-(Hydroxymethyl)-2-(Octylamino)-1,3-Propanediol

C30 H51 F O8 P1

C32 H57 F3 N3 O3 P2

2-{2-[2-(Dodecyloxy)Ethoxy]Ethoxy}Ethanol

C24 H46 N2 O1

C25 H29 Cl N9 P S

C36 H59 F3 O2

C40 H75 N O5

4-[(2-Methyl-4-Quinolinyl)Methoxy]-N-({2,4,6-Trioxo-5-[4-(3-Pyridinylcarbonyl)-1-Piperazinyl]Hexahydro-5-Pyrimidinyl}Methyl)Benzamide

C16 H33 N O5

N-[1-(1,4'-Bipiperidin-1'-yl)-3-Methyl-1-Oxo-2-Butanyl]-4-Methylcyclohexanecarboxamide

N-Methyl-N-Octyloctan-1-Amine

1-Hydroxy-2,2,6,6-Tetramethylpiperidine (Tempo)

(9Z)-Tetradec-9-Enoic Acid

C18 H41 F3 N7 O4 P

C15 H29 N5 O3

7-[(2E)-3,7-Dimethyl-2,6-Octadien-1-yl]-6-(Methoxyamino)-9-Methyl-9H-Purin-7-Ium

Octadecanamide

N,S-Ditrityl-L-Cysteine

C32 H60 O4

C50 H84 F2 O

C30 H58 O5 S

(2E,4e)-N-Isobutyl-2,4-Octadecadienamide

C29 H57 N O2

C21 H45 N3 O4

10-Oxo-10-[(3-Oxoolean-12-en-28-yl)Oxy]Decanoic Acid

C16 H38 F N5 O1

C37 H43 Cl F N S3

		NP-006888
		C19 H30 F1
		C19 H41 N
		C19 H41 N3 O5
		C16 H27 F3 O1
		[(E)-Hept-1-Enyl]Benzene
		2-(Dibutylamino)Ethanol
		N,N'-(2-Hydroxy-1,3-Propanediyl)Dioctanamide
		C16 H33 Cl F4 N4 O3 S2
		C12 H34 N9 O4 P S
		Cyclohexanecarboxamide, N-Ethyl-5-Methyl-2-(1-Methylethyl)-
		5-(2-Ethoxyphenyl)-1-Methyl-3-Propyl-1,6-Dihydro-7h-Pyrazolo[4,3-D]Pyrimidin-7-one
		(9Z)-N-[(3R)-2-Oxo-3-Azepanyl]-9-Octadecenamide
		3-Methyl-2-[(2Z)-Pent-2-en-1-yl]Cyclopent-2-en-1-one
		C30 H59 F3 O1
		C13 H25 F N6 O4
		1,4-Cyclohexadiene
		C33 H54 N4 O
		N-[(2S)-4-[(5-Chloro-2-Pyridinyl)Amino]-1,4-Dioxo-1-(1-Piperidinyl)-2-Butanyl]-2'-{[(2-Methoxyethyl)(Methyl)Amino]Methyl}-4-Biphenylcarboxamide
		Beyer-15-en-19-yl Stearate
		(5Z,9Z,12Z)-Octadeca-5,9,12-Trienoic Acid Ethyl Ester
		C31 H57 N O5
		C10 H23 F2 N6 O7 P S1
		N-(2-Hydroxyethyl)-N-Methyloctadecanamide
		C25 H53 N
		N-[(E,2S,3R)-1,3-Dihydroxyoctadec-4-en-2-yl]Acetamide
		C16 H29 F N2 O3 S1
		C34 H68 N2 O1
		NP-016596
		C19 H39 O4 P2
		C12 H27 F3 N4 S
		N-(2-Hydroxyethyl)Octadecanamide
		(E)-3-(2-Hydroxyphenyl)Prop-2-Enoic Acid (O-Coumaric Acid)
		N-(6-Amino-1-Isobutyl-2,4-Dioxo-1,2,3,4-Tetrahydro-5-Pyrimidinyl)-3-Cyclohexyl-N-Isobutylpropanamide
		(Z)-N-(2-Hydroxyethyl)Icos-11-enamide
		C29 H37 F N5
		C42 H85 F N2 O P2 S
		(5S)-4-(Cyclohexylmethyl)-1-{4-[(2S)-1-(Cyclohexylmethyl)-5,6-Dioxo-2-Piperazinyl]Butyl}-5-Isobutyl-2,3-Piperazinedione
		9-{3-[(4-Cyclohexylbutanoyl)Amino]-3-Deoxy-Beta-D-Xylofuranosyl}-9H-Purin-6-Amine
		C32 H34 N3 P
		C36 H77 N7 O P1
		C38 H65 F3 O3
		(9Z,12Z,15Z)-N-(2-Hydroxyethyl)Octadeca-9,12,15-Trienamide
		[(3S,4S,6S)-3,4-Dihydroxy-6-[5-Hydroxy-2-(4-Hydroxyphenyl)-4-Oxochromen-7-yl]Oxy-5-[(E)-3-(4-Hydroxyphenyl)Prop-2-Enoyl]Oxyoxan-2-yl]Methyl (E)-3-(4-Hydroxyphenyl)Prop-2-Enoate
MeOH	14	C17 H36 F O P2

		C20 H39 N O2 P1
		(4R,4'S)-2'-(3-Chlorophenyl)-5'-Phenyl-4'-(2-Thienyl)-2,2',3,4'-Tetrahydro-5h-Spiro[1-Benzothiepine-4,3'-Pyrazol]-5-one
		N-Dodecyl-3,4,5-Trihydroxybenzamide
		C31 H33 Cl F N O S1
		C27 H51 N O2
		3-[(3S,4S,21R)-14-Ethyl-21-(methoxycarbonyl)-4,8,13,18-tetramethyl-20-oxo-9-vinyl-3-phorbinyl]propanoic acid (Pheophorbide A)
		Hexadecanedioic Acid
		5,6-Epoxy-24(R)-Methylcholesta-7,22-Dien-3î’-ol
		2-Amino-4,6-Dimethylpyrimidine
		N-(Tert-Butyl)-N'-(2-Hydroxyethyl)Urea
		3-(1-Hydroxyethyl)-2,3,6,7,8,8a-Hexahydropyrrolo[1,2-A]Pyrazine-1,4-dione
		2,3,6-Trimethylphenol
		C20 H42 F4 N10 O2 P1
EtOH	18	C26 H36 F2 N8 O3 P1
		N-(3-Methylbutanoyl)-D-Valyl-N-[(3s)-1-{[(2r)-1-{[(2r)-1,2-Dihydroxy-5-Methyl-3-Hexanyl]Amino}-1-Oxo-2-Propanyl]Amino}-3-Hydroxy-6-Methyl-1-Oxo-4-Heptanyl]-D-Valinamide
		3-(4-Tert-Butylphenyl)-1-(4-Methoxyphenyl)Propane-1,3-dione
		C13 H29 F O P1
		C38 H35 F3 N2 O
		C27 H47 Cl N O3 P S3
		N-[(2R)-2,3-Dihydroxypropyl]-5-Fluoro-2-[(2-{[4-(4-Isopropyl-1-Piperazinyl)-2-Methoxyphenyl]Amino}-1h-Pyrrolo[2,3-D]Pyrimidin-4-yl)Amino]Benzamide
		C39 H80 F3 N O P2 S
		C32 H35 F2 N4 O4 P
		C39 H74 F3 N9 O3
		C20 H46 F2 N10 O2 S2
		C28 H52 F6 N3 O P2
		C37 H28 Cl N5 S
		C36 H76 F2 N3 O7 P
		C26 H58 F N5 O11
		3-Hydroxy-2,2-Bis[(Nonanoyloxy)Methyl]Propyl Nonanoate
		1-(3-Cyano-3,3-Diphenylpropyl)-4-Phenylpiperidine-4-Carboxylic Acid
		2-Methylpropanamine
EA	14	C22 H48 F2 N3 O6 P
		C36 H55 O11 P
		C35 H36 N4 O4
		C42 H31 Cl F3 N3 O1
		4-Oxododecanedioic Acid
		1-Ethynyl-4-Octylbenzene
		C12 H25 F2 N2 O P
		3-(4-Hydroxy-3-Methoxyphenyl)-N-{4-[3-(4-Hydroxy-3-Methoxyphenyl)Propanamido]Butyl}Propanamide
		C6 H11 F3 O3
		11-Aminoundecanoic Acid
		C16 H24 F4 O1
		C17 H32 N2 O9

		C21 H39 F6 N10 O P
		Ethyl Hydrogen [(1S,4S)-Dispiro[Cyclohexane-1,3'-[1,2,4]Trioxolane-5',2''-Tricyclo[3.3.1.1~3,7~]Decan]-4-ylmethyl]Phosphonate
ACE	29	C34 H61 N O4
		C25 H39 F2 N
		3-(4-Hydroxyphenyl)-1-(2,4,6-Trihydroxyphenyl)Propan-1-one
		N-(2-Aminoethyl)-N'-(1,5-Dicyclohexyl-3-Pentanyl)-1,2-Ethanediamine
		1-{2-[(11AS)-5-(4-Isopropylphenyl)-1,3-Dioxo-5,6,11,11A-Tetrahydro-1h-Imidazo[1',5':1,6]Pyrido[3,4-B]Indol-2(3H)-yl]Benzoyl}-4-Piperidinecarboxylic Acid
		(3BS,5AS,7R,8R,10AR,10BS)-3b,4,5,6,7,8,9,10,10a,10b-Decahydro-7-Hydroxy-10b-Methyl-5a,8-Methano-5ah-Cyclohepta(5,6)Naphtho(2,1-B)Furan-7-Methanol
		C34 H57 F3 O2
		C40 H50 F3 N2 O3 P
		2-[1-(Aminomethyl)Cyclohexyl]Acetic Acid
		3-Hydroxy-3,4-Bis[(4-Hydroxy-3-Methoxyphenyl)Methyl]Oxolan-2-one
		C44 H35 Cl2 F2 N O
		C17 H43 F5 N10 O3 P1
		N-[2-(3,4-Dimethoxyphenyl)Ethyl]-2-({2-[2-(1-Piperidinyl)Ethyl][1,2,4]Triazolo[1,5-C]Quinazolin-5-yl}Sulfanyl)Acetamide
		C12 H15 Cl2 F2 N6 O2 P
		C26 H33 Cl F N7 O3 S1
		Bicyclohexyliden-2-one
		C34 H54 F2 N3
		3,5,7-Trihydroxy-2-(4-Hydroxyphenyl)-4H-1-Benzopyran-4-one
		3-(Dimethylamino)Propyl Acrylate
		C30 H57 F3 O4
		NP-015468
		C41 H78 F N O4 P1
		4-(Hydroxyamino)-1-({1-Hydroxy-1-[2-(Hydroxymethyl)-1-Pyrrolidinyl]-3-Methyl-2-Butanyl}Amino)-2-Pentyl-1,4-Butanediol
		(2E,4S)-4-[(2-Oxohexadecanoyl)Amino]-2-Octenoic Acid
		C33 H56 N4 O2
		C36 H57 F3 O2
		(5alpha,17alpha)-3,3-Difluoropregn-20-Yn-17-ol
		5,7-Dihydroxy-2-(4-Hydroxyphenyl)-8-[3,4,5-Trihydroxy-6-(Hydroxymethyl)Oxan-2-yl]-6-(3,4,5-Trihydroxyoxan-2-yl)Chromen-4-one
		4-Oxo-3,4-Dihydroquinazoline-2-Carbohydrazide

Table S3. Identified metabolites in *E. foeminea* crude extracts analyzed by negative mode LC–MS. **(a)** acetone.

Compound Name	Formula	RT [min]
(2R,3S,4S,5R)-hexane-1,2,3,4,5,6-hexol	C6 H14 O6	0.596
Unknown	C2 H N3 O5 P2	0.661
Unknown	C3 H4 N2 O8 P2 S3	0.664
Unknown	C3 H N3 O6 P2	0.669
9,10,13-trihydroxy-11-octadecenoic acid	C18 H34 O5	7.418
Hexadecanedioic acid	C16 H30 O4	7.543
10,16-Dihydroxyhexadecanoic acid	C16 H32 O4	7.684
dihexyl (E)-but-2-enedioate	C16 H28 O4	7.732

[(2S,3R,4S,5S,6R)-2-[5,7-dihydroxy-2-(4-hydroxyphenyl)-4-oxochromen-8-yl]-4,5-dihydroxy-6-(hydroxymethyl)oxan-3-yl] (E)-3-(4-hydroxyphenyl)prop-2-enoate	C30 H26 O12	7.798
Unknown	C36 H27 N2 O6 P S	7.798
[(2S,3R,4S,5S,6R)-2-[5,7-dihydroxy-2-(4-hydroxyphenyl)-4-oxochromen-8-yl]-4,5-dihydroxy-6-(hydroxymethyl)oxan-3-yl] (E)-3-(4-hydroxyphenyl)prop-2-enoate	C30 H26 O12	7.887
Hexadecanedioic acid	C16 H30 O4	7.92
(3-hydroxy-9-methyldecyl) 4-hydroxybenzoate	C18 H28 O4	8.196
dipentyl benzene-1,2-dicarboxylate	C18 H26 O4	8.64
exo-1,7,7-Trimethylbicyclo(2.2.1)hept-2-yl acrylate	C13 H20 O2	8.754
8-Hydroxyluteolin 4'-methyl ether 7- (6'''-acetylallosyl) (1->2) (6''-acetylglucoside)	C32 H36 O19	9.171
6-Hydroxyluteolin 6-glucoside-7- [6'''- (3-hydroxy-3-methylglutaryl) glucoside]	C33 H38 O21	9.176
Unknown	C42 H31 F6 O7 P	9.176
Unknown	C29 H29 Cl N10 O13	9.178
11β,17,20R,21-tetrahydroxy-pregna-1,4-dien-3-one	C21 H30 O5	9.998
(9Z)-(13S)-12,13-Epoxyoctadeca-9,11-dienoic acid	C18 H30 O3	10
(9S,10S)-9,10-Dihydroxyoctadecanoate	C18 H36 O4	10.016
(9Z)-(13S)-12,13-Epoxyoctadeca-9,11-dienoic acid	C18 H30 O3	10.078
13S-hydroperoxy-9Z,11E,14Z-octadecatrienoic acid	C18 H30 O4	10.266
(6R,10R,11S,13S,17R)-6,11,17-trihydroxy-17-(2-hydroxyacetyl)-10,13-dimethyl-2,6,7,8,9,11,12,14,15,16-decahydro-1H-cyclopenta[a]phenanthren-3-one	C21 H30 O6	10.267
Unknown	C14 H25 F3 O10	10.271
(9Z,15Z)-(13S)-12,13-Epoxyoctadeca-9,11,15-trienoic acid	C18 H28 O3	10.274
(8S,9S,10R,11S,13S,14S,17R)-17-[(1S)-1,2-dihydroxyethyl]-11,17-dihydroxy-10,13-dimethyl-2,6,7,8,9,11,12,14,15,16-decahydro-1H-cyclopenta[a]phenanthren-3-one	C21 H32 O5	10.575
(2E)-3-(3,4-Dihydroxyphenyl)prop-2-enoic acid	C18 H32 O4 Si3	10.578
12R,13S-epoxy-9Z-octadecenoic acid	C18 H32 O3	10.58
(9Z)-(13S)-12,13-Epoxyoctadeca-9,11-dienoic acid	C18 H30 O3	10.81
Unknown	C35 H63 F O9	10.812
(7S,8S,9Z,12Z)-7,8-dihydroxyoctadeca-9,12-dienoic acid	C18 H32 O4	10.813
Unknown	C17 H26 F2 O9	10.813
2-Oxooctadecanoic acid	C18 H34 O3	11.148
12R,13S-epoxy-9Z-octadecenoic acid	C18 H32 O3	11.356
12,13-dihydroxy-9Z-octadecenoic acid	C18 H34 O4	11.358
12R,13S-epoxy-9Z-octadecenoic acid	C18 H32 O3	11.432
12,13-dihydroxy-9Z-octadecenoic acid	C18 H34 O4	11.435
(8S,10S,13S,14S,17S)-17-acetyl-10,13-dimethyl-1,2,6,7,8,12,14,15,16,17-decahydrocyclopenta[a]phenanthren-3-one	C21 H28 O2	11.457
Unknown	C14 H25 F3 O8	12.412
9Z,11E-octadecadienoic acid	C18 H32 O2	13.071
Unknown	C6 H4 Fe S	13.075
Unknown	C17 H26 F2 O7	13.078
Unknown	C6 H4 Fe S	13.743
Unknown	C15 H26 F2 O7	13.748

(b) dichloromethane/methanol extract.

Compound Name	Formula	RT [min]
(2R,3S,4S,5R)-hexane-1,2,3,4,5,6-hexol	C6 H14 O6	0.602
Unknown	C3 H4 N2 O8 P2 S3	0.666
Unknown	C3 H N3 O6 P2	0.672
Tetrachloro(hydrido)iron	H Cl4 Fe	1.471

1(2H)-Isoquinolinone	C9 H7 N O	5.355
Nonanedioic acid	C9 H16 O4	5.845
9,10,13-trihydroxy-11-octadecenoic acid	C18 H34 O5	7.424
Hexadecanedioic acid	C16 H30 O4	7.544
10,16-Dihydroxyhexadecanoic acid	C16 H32 O4	7.686
dihexyl (E)-but-2-enedioate	C16 H28 O4	7.732
[(2S,3R,4S,5S,6R)-2-[5,7-dihydroxy-2-(4-hydroxyphenyl)-4-oxochromen-8-yl]-4,5-dihydroxy-6-(hydroxymethyl)oxan-3-yl] (E)-3-(4-hydroxyphenyl)prop-2-enoate	C30 H26 O12	7.801
Hexadecanedioic acid	C16 H30 O4	7.927
(3-hydroxy-9-methyldecyl) 4-hydroxybenzoate	C18 H28 O4	8.2
dipentyl benzene-1,2-dicarboxylate	C18 H26 O4	8.644
exo-1,7,7-Trimethylbicyclo(2.2.1)hept-2-yl acrylate	C13 H20 O2	8.758
8-Hydroxyluteolin 4'-methyl ether 7- (6'''-acetylallosyl) (1->2) (6''-acetylglucoside)	C32 H36 O19	9.18
Unknown	C30 H32 Cl F N10 O7 P2	9.18
Unknown	C36 H34 F2 O18	9.18
6-Hydroxyluteolin 6-glucoside-7- [6'''- (3-hydroxy-3-methylglutaryl) glucoside]	C33 H38 O21	9.182
(9Z)-(13S)-12,13-Epoxyoctadeca-9,11-dienoic acid	C18 H30 O3	10.004
(9S,10S)-9,10-Dihydroxyoctadecanoate	C18 H36 O4	10.021
(9Z)-(13S)-12,13-Epoxyoctadeca-9,11-dienoic acid	C18 H30 O3	10.083
13S-hydroperoxy-9Z,11E,14Z-octadecatrienoic acid	C18 H30 O4	10.267
(6R,10R,11S,13S,17R)-6,11,17-trihydroxy-17-(2-hydroxyacetyl)-10,13-dimethyl-2,6,7,8,9,11,12,14,15,16-decahydro-1H-cyclopenta[a]phenanthren-3-one	C21 H30 O6	10.272
(9Z,15Z)-(13S)-12,13-Epoxyoctadeca-9,11,15-trienoic acid	C18 H28 O3	10.279
(8S,9S,10R,11S,13S,14S,17R)-17-[(1S)-1,2-dihydroxyethyl]-11,17-dihydroxy-10,13-dimethyl-2,6,7,8,9,11,12,14,15,16-decahydro-1H-cyclopenta[a]phenanthren-3-one	C21 H32 O5	10.581
12R,13S-epoxy-9Z-octadecenoic acid	C18 H32 O3	10.583
(2E)-3-(3,4-Dihydroxyphenyl)prop-2-enoic acid	C18 H32 O4 Si3	10.584
(9Z)-(13S)-12,13-Epoxyoctadeca-9,11-dienoic acid	C18 H30 O3	10.812
(7S,8S,9Z,12Z)-7,8-dihydroxyoctadeca-9,12-dienoic acid	C18 H32 O4	10.814
Unknown	C12 H24 N6 O10	10.817
(9Z)-(13S)-12,13-Epoxyoctadeca-9,11-dienoic acid	C18 H30 O3	10.921
(9Z)-(13S)-12,13-Epoxyoctadeca-9,11-dienoic acid	C18 H30 O3	11.053
2-Oxooctadecanoic acid	C18 H34 O3	11.151
12R,13S-epoxy-9Z-octadecenoic acid	C18 H32 O3	11.436
(8S,10S,13S,14S,17S)-17-acetyl-10,13-dimethyl-1,2,6,7,8,12,14,15,16,17-decahydrocyclopenta[a]phenanthren-3-one	C21 H28 O2	11.46
Unknown	C14 H25 F3 O8	12.415
Unknown	C6 H4 Fe S	13.08
Unknown	C17 H26 F2 O7	13.082
Unknown	C6 H4 Fe S	13.746
Unknown	C15 H26 F2 O7	13.75

(c) ethanol extract.

Compound Name	Formula	RT [min]
(2R,3S,4S,5R)-hexane-1,2,3,4,5,6-hexol	C6 H14 O6	0.597
Unknown	C4 H6 N O P3 S	0.662
Unknown	C5 H F4 N2 O2 P3 S2	0.668
Unknown	C3 H N3 O6 P2	0.671
Tetrachloro(hydrido)iron	H Cl4 Fe	1.464
1(2H)-Isoquinolinone	C9 H7 N O	5.413
9,10,13-trihydroxy-11-octadecenoicacid	C18 H34 O5	7.418
Hexadecanedioic acid	C16 H30 O4	7.543
10,16-Dihydroxyhexadecanoicacid	C16 H32 O4	7.685

[(2S,3R,4S,5S,6R)-2-[5,7-dihydroxy-2-(4-hydroxyphenyl)-4-oxochromen-8-yl]-4,5-dihydroxy-6-(hydroxymethyl)oxan-3-yl] (E)-3-(4-hydroxyphenyl)prop-2-enoate	C30 H26 O12	7.8
Unknown	C30 H27 F O15	7.8
[(2S,3R,4S,5S,6R)-2-[5,7-dihydroxy-2-(4-hydroxyphenyl)-4-oxochromen-8-yl]-4,5-dihydroxy-6-(hydroxymethyl)oxan-3-yl] (E)-3-(4-hydroxyphenyl)prop-2-enoate	C30 H26 O12	7.889
Hexadecanedioic acid	C16 H30 O4	7.924
(3-hydroxy-9-methyldecyl) 4-hydroxybenzoate	C18 H28 O4	8.198
dipentyl benzene-1,2-dicarboxylate	C18 H26 O4	8.642
exo-1,7,7-Trimethylbicyclo(2.2.1)hept-2-yl acrylate	C13 H20 O2	8.755
8-Hydroxyluteolin 4'-methyl ether 7- (6'''-acetylallosyl) (1->2) (6''-acetylglucoside)	C32 H36 O19	9.179
Unknown	C46 H27 Cl F6 O2	9.181
(9Z)-(13S)-12,13-Epoxyoctadeca-9,11-dienoicacid	C18 H30 O3	10.008
(9Z)-(13S)-12,13-Epoxyoctadeca-9,11-dienoicacid	C18 H30 O3	10.075
13S-hydroperoxy-9Z,11E,14Z-octadecatrienoicacid	C18 H30 O4	10.265
(6R,10R,11S,13S,17R)-6,11,17-trihydroxy-17-(2-hydroxyacetyl)-10,13-dimethyl-2,6,7,8,9,11,12,14,15,16-decahydro-1H-cyclopenta[a]phenanthren-3-one	C21 H30 O6	10.266
(9Z,15Z)-(13S)-12,13-Epoxyoctadeca-9,11,15-trienoicacid	C18 H28 O3	10.277
12R,13S-epoxy-9Z-octadecenoicacid	C18 H32 O3	10.578
(2E)-3-(3,4-Dihydroxyphenyl)prop-2-enoic acid	C18 H32 O4 Si3	10.58
(8S,9S,10R,11S,13S,14S,17R)-17-[(1S)-1,2-dihydroxyethyl]-11,17-dihydroxy-10,13-dimethyl-2,6,7,8,9,11,12,14,15,16-decahydro-1H-cyclopenta[a]phenanthren-3-one	C21 H32 O5	10.581
(9Z)-(13S)-12,13-Epoxyoctadeca-9,11-dienoicacid	C18 H30 O3	10.809
Unknown	C16 H28 O12	10.809
(7S,8S,9Z,12Z)-7,8-dihydroxyoctadeca-9,12-dienoic acid	C18 H32 O4	10.811
(9Z)-(13S)-12,13-Epoxyoctadeca-9,11-dienoicacid	C18 H30 O3	11.049
2-Oxooctadecanoic acid	C18 H34 O3	11.153
12R,13S-epoxy-9Z-octadecenoicacid	C18 H32 O3	11.35
12,13-dihydroxy-9Z-octadecenoic acid	C18 H34 O4	11.425
12R,13S-epoxy-9Z-octadecenoicacid	C18 H32 O3	11.432
(8S,10S,13S,14S,17S)-17-acetyl-10,13-dimethyl-1,2,6,7,8,12,14,15,16,17-decahydrocyclopenta[a]phenanthren-3-one	C21 H28 O2	11.455
Unknown	C14 H25 F3 O8	12.405
Unknown	C17 H26 F2 O7	13.069
(9Z),(11E)-octadecadienoic acid	C18 H32 O2	13.07
Unknown	C6 H4 Fe S	13.739
Unknown	C15 H26 F2 O7	13.742

(d) ethyl acetate extract.

Compound Name	Formula	RT [min]
(2R,3S,4S,5R)-hexane-1,2,3,4,5,6-hexol	C6 H14 O6	0.591
Unknown	C2 H N3 O5 P2	0.657
Unknown	C3 H4 N2 O8 P2 S3	0.66
Unknown	C5 H6 N O2 P3 S	0.665
9,10,13-trihydroxy-11-octadecenoic acid	C18 H34 O5	7.407
Unknown	C18 H25 F3 N4	7.527
Hexadecanedioic acid	C16 H30 O4	7.527
10,16-Dihydroxyhexadecanoic acid	C16 H32 O4	7.668
dihexyl (E)-but-2-enedioate	C16 H28 O4	7.713
[(2S,3R,4S,5S,6R)-2-[5,7-dihydroxy-2-(4-hydroxyphenyl)-4-oxochromen-8-yl]-4,5-dihydroxy-6-(hydroxymethyl)oxan-3-yl] (E)-3-(4-hydroxyphenyl)prop-2-enoate	C30 H26 O12	7.781
Unknown	C34 H22 Cl F3 N2 O4	7.781
Unknown	C25 H32 N2 O14 P2	7.785
[(2S,3R,4S,5S,6R)-2-[5,7-dihydroxy-2-(4-hydroxyphenyl)-4-oxochromen-8-yl]-4,5-dihydroxy-6-(hydroxymethyl)oxan-3-yl] (E)-3-(4-hydroxyphenyl)prop-2-enoate	C30 H26 O12	7.872

Hexadecanedioic acid	C16 H30 O4	7.907
(3-hydroxy-9-methyldecyl) 4-hydroxybenzoate	C18 H28 O4	8.184
dipentyl benzene-1,2-dicarboxylate	C18 H26 O4	8.622
exo-1,7,7-Trimethylbicyclo(2.2.1)hept-2-yl acrylate	C13 H20 O2	8.733
8-Hydroxyluteolin 4'-methyl ether 7- (6'''-acetylallosyl) (1->2) (6''-acetylglucoside)	C32 H36 O19	9.158
Unknown	C41 H32 F2 N4 O7 P2	9.159
Unknown	C46 H27 Cl F6 O2	9.16
6-Hydroxyluteolin 6-glucoside-7- [6'''- (3-hydroxy-3-methylglutaryl) glucoside]	C33 H38 O21	9.162
6Z,9Z,12Z,15Z-octadecatetraenoic acid	C18 H28 O2	9.977
11β,17,20R,21-tetrahydroxy-pregna-1,4-dien-3-one	C21 H30 O5	9.988
(9Z)-(13S)-12,13-Epoxyoctadeca-9,11-dienoic acid	C18 H30 O3	9.992
(9S,10S)-9,10-Dihydroxyoctadecanoate	C18 H36 O4	10.001
(9Z)-(13S)-12,13-Epoxyoctadeca-9,11-dienoic acid	C18 H30 O3	10.068
11β,17,20R,21-tetrahydroxy-pregna-1,4-dien-3-one	C21 H30 O5	10.072
13S-hydroperoxy-9Z,11E,14Z-octadecatrienoic acid	C18 H30 O4	10.249
(6R,10R,11S,13S,17R)-6,11,17-trihydroxy-17-(2-hydroxyacetyl)-10,13-dimethyl-2,6,7,8,9,11,12,14,15,16-decahydro-1H-cyclopenta[a]phenanthren-3-one	C21 H30 O6	10.252
(9Z,15Z)-(13S)-12,13-Epoxyoctadeca-9,11,15-trienoic acid	C18 H28 O3	10.261
Unknown	C15 H21 F3 N4 O6	10.264
Unknown	C31 H51 F N8	10.555
(8S,9S,10R,11S,13S,14S,17R)-17-[(1S)-1,2-dihydroxyethyl]-11,17-dihydroxy-10,13-dimethyl-2,6,7,8,9,11,12,14,15,16-decahydro-1H-cyclopenta[a]phenanthren-3-one	C21 H32 O5	10.556
12R,13S-epoxy-9Z-octadecenoic acid	C18 H32 O3	10.557
(2E)-3-(3,4-Dihydroxyphenyl)prop-2-enoic acid	C18 H32 O4 Si3	10.562
Unknown	C42 H62 O S	10.563
(9Z)-(13S)-12,13-Epoxyoctadeca-9,11-dienoic acid	C18 H30 O3	10.741
(7S,8S,9Z,12Z)-7,8-dihydroxyoctadeca-9,12-dienoic acid	C18 H32 O4	10.742
1-Cyclohexyl-3-[3-(1H-imidazol-1-yl)propyl]thiourea	C13 H22 N4 S	10.787
(9Z)-(13S)-12,13-Epoxyoctadeca-9,11-dienoic acid	C18 H30 O3	10.788
Unknown	C35 H62 F3 N2 O3 P	10.788
Unknown	C16 H28 O12	10.789
(7S,8S,9Z,12Z)-7,8-dihydroxyoctadeca-9,12-dienoic acid	C18 H32 O4	10.795
(9Z)-(13S)-12,13-Epoxyoctadeca-9,11-dienoic acid	C18 H30 O3	11.03
(9Z)-(13S)-12,13-Epoxyoctadeca-9,11-dienoic acid	C18 H30 O3	11.088
2-Oxooctadecanoic acid	C18 H34 O3	11.142
12,13-dihydroxy-9Z-octadecenoic acid	C18 H34 O4	11.329
12R,13S-epoxy-9Z-octadecenoic acid	C18 H32 O3	11.332
12R,13S-epoxy-9Z-octadecenoic acid	C18 H32 O3	11.407
12,13-dihydroxy-9Z-octadecenoic acid	C18 H34 O4	11.412
Unknown	C21 H34 O6	11.413
(8S,10S,13S,14S,17S)-17-acetyl-10,13-dimethyl-1,2,6,7,8,12,14,15,16,17-decahydrocyclopenta[a]phenanthren-3-one	C21 H28 O2	11.439
Unknown	C14 H25 F3 O8	12.382
9Z-Octadecen-12-ynoic acid	C18 H30 O2	12.383
(2Z,4E)-5-[(1S,4S,6R)-4-Hydroxy-2,2,6-trimethyl-7-oxabicyclo[4.1.0]heptan-1-yl]-3-methylpenta-2,4-dienal	C15 H22 O3	12.952
9Z,11E-octadecadienoic acid	C18 H32 O2	13.045
Unknown	C14 H27 F3 O8	13.05
Unknown	C6 H4 Fe S	13.052
2,2'-Methylenebis(6-tert-butyl-4-ethylphenol)	C25 H36 O2	13.19
Unknown	C34 H37 N6 O2 P	13.396
Unknown	C15 H26 F2 O7	13.711

Unknown	C6 H4 Fe S	13.719

(e) methanol extract.

Compound Name	Formula	RT [min]
(2R,3S,4S,5R)-hexane-1,2,3,4,5,6-hexol	C6 H14 O6	0.598
Unknown	C2 H N3 O5 P2	0.66
Unknown	C3 H4 N2 O8 P2 S3	0.667
Unknown	C3 H N3 O6 P2	0.669
Tetrachloro(hydrido)iron	H Cl4 Fe	1.469
1(2H)-Isoquinolinone	C9 H7 N O	5.351
9,10,13-trihydroxy-11-octadecenoic acid	C18 H34 O5	7.416
Hexadecanedioic acid	C16 H30 O4	7.541
10,16-Dihydroxyhexadecanoic acid	C16 H32 O4	7.681
[(2S,3R,4S,5S,6R)-2-[5,7-dihydroxy-2-(4-hydroxyphenyl)-4-oxochromen-8-yl]-4,5-dihydroxy-6-(hydroxymethyl)oxan-3-yl] (E)-3-(4-hydroxyphenyl)prop-2-enoate	C30 H26 O12	7.799
Hexadecanedioic acid	C16 H30 O4	7.922
(3-hydroxy-9-methyldecyl) 4-hydroxybenzoate	C18 H28 O4	8.196
dipentyl benzene-1,2-dicarboxylate	C18 H26 O4	8.638
exo-1,7,7-Trimethylbicyclo(2.2.1)hept-2-yl acrylate	C13 H20 O2	8.752
8-Hydroxyluteolin 4'-methyl ether 7- (6'''-acetylallosyl) (1->2) (6''-acetylglucoside)	C32 H36 O19	9.172
Unknown	C46 H26 F5 N4 O2 P	9.173
6Z,9Z,12Z,15Z-octadecatetraenoic acid	C18 H28 O2	9.995
11β,17,20R,21-tetrahydroxy-pregna-1,4-dien-3-one	C21 H30 O5	9.999
(9Z)-(13S)-12,13-Epoxyoctadeca-9,11-dienoic acid	C18 H30 O3	10.003
(9S,10S)-9,10-Dihydroxyoctadecanoate	C18 H36 O4	10.013
(9Z)-(13S)-12,13-Epoxyoctadeca-9,11-dienoic acid	C18 H30 O3	10.084
13S-hydroperoxy-9Z,11E,14Z-octadecatrienoic acid	C18 H30 O4	10.262
(6R,10R,11S,13S,17R)-6,11,17-trihydroxy-17-(2-hydroxyacetyl)-10,13-dimethyl-2,6,7,8,9,11,12,14,15,16-decahydro-1H-cyclopenta[a]phenanthren-3-one	C21 H30 O6	10.265
(9Z,15Z)-(13S)-12,13-Epoxyoctadeca-9,11,15-trienoic acid	C18 H28 O3	10.274
(8S,9S,10R,11S,13S,14S,17R)-17-[(1S)-1,2-dihydroxyethyl]-11,17-dihydroxy-10,13-dimethyl-2,6,7,8,9,11,12,14,15,16-decahydro-1H-cyclopenta[a]phenanthren-3-one	C21 H32 O5	10.572
12R,13S-epoxy-9Z-octadecenoic acid	C18 H32 O3	10.576
(2E)-3-(3,4-Dihydroxyphenyl)prop-2-enoic acid	C18 H32 O4 Si3	10.577
Unknown	C38 H62 O6	10.578
(7S,8S,9Z,12Z)-7,8-dihydroxyoctadeca-9,12-dienoic acid	C18 H32 O4	10.804
Unknown	C16 H28 O12	10.804
(9Z)-(13S)-12,13-Epoxyoctadeca-9,11-dienoic acid	C18 H30 O3	10.805
(9Z)-(13S)-12,13-Epoxyoctadeca-9,11-dienoic acid	C18 H30 O3	10.917
(9Z)-(13S)-12,13-Epoxyoctadeca-9,11-dienoic acid	C18 H30 O3	11.047
2-Oxooctadecanoic acid	C18 H34 O3	11.149
12R,13S-epoxy-9Z-octadecenoic acid	C18 H32 O3	11.431
(8S,10S,13S,14S,17S)-17-acetyl-10,13-dimethyl-1,2,6,7,8,12,14,15,16,17-decahydrocyclopenta[a]phenanthren-3-one	C21 H28 O2	11.455
9Z-Octadecen-12-ynoic acid	C18 H30 O2	12.406
Unknown	C17 H24 F2 O7	12.406
Unknown	C17 H26 F2 O7	13.071
Unknown	C6 H4 Fe S	13.071
2,2'-Methylenebis(6-tert-butyl-4-ethylphenol)	C25 H36 O2	13.207
Unknown	C6 H4 Fe S	13.739
Unknown	C15 H26 F2 O7	13.741

Table S4. Common and unique compounds identified in different *E. foeminea* crude extracts via Venn diagram analysis using negative ion mode LC–MS.

Occurrence	Total	Compound Name
ACE DCM/MeOH EA EtOH MeOH	15	2-Oxooctadecanoic Acid
		(7S,8S,9Z,12Z)-7,8-dihydroxyoctadeca-9,12-dienoic acid
		(8S,10S,13S,14S,17S)-17-Acetyl-10,13-dimethyl-1,2,6,7,8,12,14,15,16,17-decahydrocyclopenta[a]phenanthren-3-one
		8-Hydroxyluteolin 4'-Methyl Ether 7- (6'''-Acetylallosyl) (1->2) (6''-Acetylglucoside)
		(2E)-3-(3,4-Dihydroxyphenyl)Prop-2-Enoic Acid
		Dipentyl Benzene-1,2-Dicarboxylate
		C6 H4 Fe S
		(3-Hydroxy-9-Methyldecyl) 4-Hydroxybenzoate
		C15 H26 F2 O6
		(8S,9S,10R,11S,13S,14S,17R)-17-[(1S)-1,2-Dihydroxyethyl]-11,17-Dihydroxy-10,13-Dimethyl-2,6,7,8,9,11,12,14,15,16-Decahydro-1H-Cyclopenta[a]phenanthren-3-one
		Exo-1,7,7-Trimethylbicyclo(2.2.1)Hept-2-yl Acrylate
		[(2S,3R,4S,5S,6R)-2-[5,7-dihydroxy-2-(4-hydroxyphenyl)-4-oxochromen-8-yl]-4,5-dihydroxy-6-(hydroxymethyl)oxan-3-yl] (E)-3-(4-hydroxyphenyl)prop-2-enoate
		(2R,3R,4R,5R)-hexane-1,2,3,4,5,6-hexol
		Hexadecanedioic Acid
		(6R,8S,10R,11S,13S,17R)-6,11,17-trihydroxy-17-(2-hydroxyacetyl)-10,13-dimethyl-2,6,7,8,9,11,12,14,15,16-decahydro-1H-cyclopenta[a]phenanthren-3-one.
ACE DCM/MeOH EtOH MeOH	2	C3 H N3 O6 P1
		C17 H26 F2 O6
ACE DCM/MeOH EA MeOH	6	13S-hydroperoxy-9Z,11E,14Z-octadecatrienoic acid
		(9Z,13S)-12,13-epoxyoctadeca-9,11-dienoic acid
		10,16-Dihydroxyhexadecanoic Acid
		C3 H4 N2 O8 P2 S2
		(9S,10S)-9,10-Dihydroxyoctadecanoate
		12(R),13(S)-Epoxy-9(Z)-octadecenoic acid
DCM/MeOH EtOH MeOH	2	1(2H)-Isoquinolinone
		Tetrachloro(Hydrido)Iron
ACE DCM/MeOH EA	5	C14 H25 F3 O7
		(9Z,15Z)-(13S)-12,13-Epoxyoctadeca-9,11,15-Trienoic Acid
		6-Hydroxyluteolin 6-Glucoside-7- [6'''- (3-Hydroxy-3-Methylglutaryl) Glucoside
		9,10,13-Trihydroxy-11-Octadecenoic Acid
		Dihexyl (E)-But-2-Enedioate
ACE EA MeOH	2	11î’,17,20r,21-Tetrahydroxy-Pregna-1,4-Dien-3-one
		C2 H N3 O5 P1
ACE EA EtOH	1	12,13-Dihydroxy-9Z-Octadecenoic Acid
EA MeOH	4	6Z,9Z,12Z,15Z-Octadecatetraenoic Acid
		9Z-Octadecen-12-Ynoic Acid
		C16 H28 O11
		2,2'-Methylenebis(6-Tert-Butyl-4-Ethylphenol)
ACE EA	1	9Z,11E-Octadecadienoic Acid
DCM/MeOH	4	Nonanedioic Acid

		C36 H34 F2 O17
		C12 H24 N6 O9
		C30 H32 Cl F N10 O7 P1
MeOH	6	C46 H26 F5 N4 O2 P
		(9Z)-(13S)-12,13-Epoxyoctadeca-9,11-Dienoic Acid
		9,10,13-Trihydroxy-11-Octadecenoic Acid
		(9Z,15Z)-(13S)-12,13-Epoxyoctadeca-9,11,15-Trienoic Acid
		C38 H62 O5
		C17 H24 F2 O6
EtOH	14	C4 H6 N O P3 S
		C14 H25 F3 O8
		12(R),13(S)-Epoxy-9(Z)-octadecenoic acid
		C30 H27 F O15
		Hexadecanedioicacid
		(9Z,15Z)-(13S)-12,13-Epoxyoctadeca-9,11,15-Trienoicacid
		(9Z),(11E)-Octadecadienoicacid
		(9Z)-(13S)-12,13-Epoxyoctadeca-9,11-Dienoicacid
		C5 H F4 N2 O2 P3 S1
		13S-Hydroperoxy-9Z,11E,14z-Octadecatrienoicacid
		10,16-Dihydroxyhexadecanoicacid
		9,10,13-Trihydroxy-11-Octadecenoicacid
		C46 H27 Cl F6 O2
		C16 H28 O12
EA	15	C42 H62 O S
		C35 H62 F3 N2 O3 P
		1-Cyclohexyl-3-[3-(1h-Imidazol-1-yl)Propyl]Thiourea
		(2Z,4E)-5-[(1S,4S,6R)-4-hydroxy-2,2,6-trimethyl-7-oxabicyclo[4.1.0]hept-1-yl]-3-methylpenta-2,4-dienal
		C41 H32 F2 N4 O7 P1
		C15 H21 F3 N4 O5
		C5 H6 N O2 P3 S
		C46 H27 Cl F6 O1
		C31 H51 F N7
		C34 H22 Cl F3 N2 O3
		C25 H32 N2 O14 P1
		C34 H37 N6 O2 P
		C18 H25 F3 N3
		C14 H27 F3 O7
		C21 H34 O5
ACE	6	C35 H63 F O8
		C14 H25 F3 O9
		C29 H29 Cl N10 O12
		C36 H27 N2 O6 P S
		C17 H26 F2 O8
		C42 H31 F6 O7 P

Table S5. Identified metabolites in *E. foeminea* crude extracts analyzed by GC–MS. **(a)** acetone.

Compound Name	RT(min)

Boric Acid	8.190467
1,2-Xylene	8.374233
Pyridine	8.4141
Pyridine	8.418533
Pyridine	8.859117
O-Methylhydroxylamine	8.960967
O-Methylhydroxylamine	8.9632
Propan-1-amine	9.19345
1H-pyridin-2-one	9.556583
2-Hydroxypropanoic acid	10.15885
2-Hydroxypropanoic acid	10.16107
Hydroxyacetic acid	10.25185
Hydroxyacetic acid	10.6039
2-Methylpropan-1-amine	11.11098
1-Butyl-ethylhydrazine	11.9768
Oxalic Acid	12.03657
1,3-diazinane-2,4-dione	12.30452
Pyrrolidin-2-one	12.39528
3-Hydroxypropionic Acid	12.43515
(2S)-2-(methylamino)propanoic acid	13.18135
Benzoic Acid	15.0768
Propane-1,2,3-triol	15.69238
Propane-1,2,3-triol	15.69903
(2S,3R)-butane-1,2,3-triol	16.08435
2-oxohexanoic acid	16.5826
Butanedioic acid	16.6446
Pentanedioic acid	18.49585
(2R,3S)-Butane-1,2,3,4-tetrol	20.73677
(2R,3R)-Butane-1,2,3,4-tetrol	20.90947
5-OxoProline	21.18407
(2R,3S,4S)-Pentane-1,2,3,4,5-pentol	25.1477
(3R,4S,5R)-3,4,5-Trihydroxycyclohex-1-ene-1-carboxylic acid	26.66452
(3S,4R,5R)-1,3,4,5,6-Pentahydroxyhexan-2-one	27.74062
(3S,4R,5S)-1,3,4,5,6-Pentahydroxyhexan-2-one	27.90448
(2R,3S,4R,5R)-2,3,4,5,6-Pentahydroxyhexanal	28.16353
(3S,4S,5S,6R)-6-(hydroxymethyl)oxane-2,3,4,5-tetrol	28.16797
(2R,3S,4S,5R)-Hexane-1,2,3,4,5,6-hexol	28.84115
(2S,3R,4R,5R)-Hexane-1,2,3,4,5,6-hexol	28.84778
Hexadecanoic acid	30.65027
Hexadecanoic acid	30.66133
Hexadecanoic acid	30.66577
Hexadecanoic acid	30.67018
(9Z,12Z)-Octadeca-9,12-dienoic acid	33.16796
(9Z,12Z)-Octadeca-9,12-dienoic acid	33.17019
(9Z,12Z,15Z)-Octadeca-9,12,15-trienoic acid	33.25875
Octadecanoic acid	33.64403
Octadecanoic acid	33.64847

(b) dichloromethane/methanol extract.

Compound Name	RT(min)
Pyridine	8.633533
1H-pyridin-2-one	9.604883
2-Hydroxypropanoic acid	9.963717
Hydroxyacetic acid	10.4896

2-Methylpropan-1-amine	10.85463
Oxalic Acid	11.88372
1,3-diazinane-2,4-dione	12.26318
3-Hydroxypropionic Acid	12.30442
Pyrrolidin-2-one	12.67152
Valine	14.01618
2-Aminoethan-1-ol	15.21648
Benzoic Acid	15.2433
Propane-1,2,3-triol	15.33815
Propane-1,2,3-triol	15.3464
Aminoacetic acid	16.20847
Butanedioic acid	16.59207
2,3-Dihydroxypropanoic acid	16.71995
2-Hydroxybutanedioic acid	20.23425
(2R,3S)-Butane-1,2,3,4-tetrol	20.26312
(2R,3R)-Butane-1,2,3,4-tetrol	20.41987
(2R,3S)-Butane-1,2,3,4-tetrol	20.42192
4-Aminobutanoic acid	21.17055
4-Aminobutanoic acid	21.17262
4-Aminobutanoic acid	21.18912
5-OxoProline	21.25717
Xylose	23.48662
(2R,4R)-Pentane-1,2,3,4,5-pentol	24.19607
(2R,3s,4S)-Pentane-1,2,3,4,5-pentol	24.19813
(2R,4R)-Pentane-1,2,3,4,5-pentol	24.2002
(2R,3s,4S)-Pentane-1,2,3,4,5-pentol	24.46212
(2R,3R,4S)-Pentane-1,2,3,4,5-pentol	24.54667
(3R,4S,5R)-3,4,5-Trihydroxycyclohex-1-ene-1-carboxylic acid	26.343
3,4-DihydroxyBenzoic Acid	26.72248
(3R,4R,5R)-2-(hydroxymethyl)oxane-2,3,4,5-tetrol	27.14115
(3S,4R,5S)-1,3,4,5,6-Pentahydroxyhexan-2-one	27.1494
(3R,4R,5R)-2-(hydroxymethyl)oxane-2,3,4,5-tetrol	27.15352
(3S,4R,5S)-1,3,4,5,6-Pentahydroxyhexan-2-one	27.32057
(3S,4R,5R)-1,3,4,5,6-Pentahydroxyhexan-2-one	27.32675
(3S,4S,5S,6R)-6-(hydroxymethyl)oxane-2,3,4,5-tetrol	27.58867
(3S,4S,5S,6R)-6-(hydroxymethyl)oxane-2,3,4,5-tetrol	27.6031
(2R,3R,4S,5S,6R)-6-(hydroxymethyl)oxane-2,3,4,5-tetrol	27.9331
(2R)-2-[(1S)-1,2-dihydroxyethyl]-3,4-dihydroxy-2H-furan-5-one	28.14138
(2S,3R,4S,5R,6R)-6-(hydroxymethyl)oxane-2,3,4,5-tetrol	28.14552
(2R,3R,4R,5R)-Hexane-1,2,3,4,5,6-hexol	28.1517
(2R,3R,4R,5R)-Hexane-1,2,3,4,5,6-hexol	28.19087
(2R,3R,4R,5R)-Hexane-1,2,3,4,5,6-hexol	28.19498
(2R,3S,4S,5R)-Hexane-1,2,3,4,5,6-hexol	28.1991
(2R,3s,4S)-Pentane-1,2,3,4,5-pentol	28.20323
(2S,3R,4S,5R,6R)-2-[(2R,3S,4S,5R)-4,5-dihydroxy-2,5-bis(hydroxymethyl)oxolan-3-yl]oxy-6-(hydroxymethyl)oxane-3,4,5-triol	30.03877
Hexadecanoic acid	30.58942
Tetradecanoic acid	30.5956
(1R,2S,3r,4R,5S,6s)-Cyclohexane-1,2,3,4,5,6-hexol	30.73583
(9Z,12Z)-Octadeca-9,12-dienoic acid	33.1241
(9Z)-Octadec-9-enoic acid	33.1901
(9Z,12Z,15Z)-Octadeca-9,12,15-trienoic acid	33.24373
Octadecanoic acid	33.55927

Tetradecanoic acid	33.56133
Acetic acid	37.59743

(c) ethanol extract.

Compound Name	RT(min)
Boric Acid	8.179383
Pyridine	8.400817
Pyridine	8.845866
Propan-1-amine	9.186883
1H-pyridin-2-one	9.567717
2-Hydroxypropanoic acid	10.15008
Hydroxyacetic acid	10.59735
2-Aminopropanoic acid	11.31922
Butan-1-amine	11.4255
Oxalic Acid	12.03222
Pyrrolidin-2-one	12.39093
3-Hydroxypropionic Acid	12.42857
1,3-diazinane-2,4-dione	12.51492
3-Hydroxybutanoic acid	12.84485
Valine	14.25537
Valine	14.27085
4-Hydroxybutanoic acid	14.77795
Urea	14.84882
Benzoic Acid	15.0769
2-Aminoethan-1-ol	15.52642
Propane-1,2,3-triol	15.66812
Propane-1,2,3-triol	15.69248
Propane-1,2,3-triol	15.70575
(2S,3R)-butane-1,2,3-triol	16.08218
Isoleucine	16.15748
Proline	16.25712
2-oxohexanoic acid	16.58262
Butanedioic acid	16.64462
Nonanoic acid	17.75618
2,3-Bis(phosphonooxy)propanoic acid	18.79242
2-Hydroxybutanedioic acid	20.4975
(2R,3S)-Butane-1,2,3,4-tetrol	20.7477
(2R,3S)-Butane-1,2,3,4-tetrol	20.91155
5-OxoProline	21.18392
4-Aminobutanoic acid	21.3943
4-Hydroxybenzoic Acid	23.35617
Dodecanoic acid	23.84555
(2R,3R,4S,5R)-5-(hydroxymethyl)oxolane-2,3,4-triol	23.97398
(2R,4R)-Pentane-1,2,3,4,5-pentol	24.6405
ethenyl icosyl carbonate	24.82428
(2R,3s,4S)-Pentane-1,2,3,4,5-pentol	25.05457
(3R,4S,5R)-3,4,5-Trihydroxycyclohex-1-ene-1-carboxylic acid	26.66872
3,4-DihydroxyBenzoic Acid	26.86578
Tetradecanoic acid	27.41272
(3S,4R,5R)-1,3,4,5,6-Pentahydroxyhexan-2-one	27.74263
(3S,4R,5S)-1,3,4,5,6-Pentahydroxyhexan-2-one	27.74707
(3S,4R,5R)-1,3,4,5,6-Pentahydroxyhexan-2-one	27.9065
(2R,3S,4R,5R)-2,3,4,5,6-Pentahydroxyhexanal	27.98398
(2R,3R,4S,5S,6R)-6-(hydroxymethyl)oxane-2,3,4,5-tetrol	28.1722

(2R,3R,4S,5S,6R)-6-(hydroxymethyl)oxane-2,3,4,5-tetrol	28.50215
(2S,3R,4R,5R)-Hexane-1,2,3,4,5,6-hexol	28.82983
(2R,3S,4S,5R)-Hexane-1,2,3,4,5,6-hexol	28.83205
(2S,3R,4R,5R)-Hexane-1,2,3,4,5,6-hexol	28.84312
(2S,3R,4S,5R,6R)-6-(hydroxymethyl)oxane-2,3,4,5-tetrol	28.85197
(3R,4R,5R,6R)-3-amino-6-(hydroxymethyl)oxane-2,4,5-triol	28.86745
Hexadecanoic acid	30.66323
Hexadecanoic acid	30.66767
Hexadecanoic acid	30.67208
Hexadecanoic acid	30.68315
(1R,2S,3r,4R,5S,6s)-Cyclohexane-1,2,3,4,5,6-hexol	31.332
(9Z,12Z)-Octadeca-9,12-dienoic acid	33.16767
(9Z,12Z)-Octadeca-9,12-dienoic acid	33.16988
(9Z,12Z)-Octadeca-9,12-dienoic acid	33.17208
(9Z)-Octadec-9-enoic acid	33.26067
Octadecanoic acid	33.63928
Octadecanoic acid	33.64594

(d) ethyl acetate extract.

Compound Name	RT(min)
Boric Acid	8.192683
Pyridine	8.42075
Pyridine	8.863584
O-Methylhydroxylamine	8.965433
Propan-1-amine	9.1935
1H-pyridin-2-one	9.556633
2-Hydroxypropanoic acid	10.15888
Hydroxyacetic acid	10.60397
Oxalic Acid	11.98125
Methylpropanedioic acid	12.03662
Oxalic Acid	12.03882
Oxalic Acid	12.31118
1,3-diazinane-2,4-dione	12.33112
3-Hydroxypropionic Acid	12.43518
Benzoic Acid	15.07902
Propane-1,2,3-triol	15.69238
3-Hydroxybutan-2-one	16.58472
Butanedioic acid	16.64673
(2R,3S)-Butane-1,2,3,4-tetrol	20.9137
(3S,4R,5R)-1,3,4,5,6-Pentahydroxyhexan-2-one	27.7426
(2S,3R,4R,5R)-Hexane-1,2,3,4,5,6-hexol	28.84083
(2R,3S,4S,5R)-Hexane-1,2,3,4,5,6-hexol	28.84305
1-O-(2-ethylhexyl) 2-O-propan-2-yl benzene-1,2-dicarboxylate	29.26375
Hexadecanoic acid	30.66537
(9Z,12Z)-Octadeca-9,12-dienoic acid	33.15873
(9Z,12Z)-Octadeca-9,12-dienoic acid	33.1698
(9Z,12Z)-Octadeca-9,12-dienoic acid	33.172
(9Z)-Octadec-9-enoic acid	33.26058
Octadecanoic acid	33.62597
Octadecanoic acid	33.63925
Octadecanoic acid	33.65032
Octadecanoic acid	33.65918

(e) methanol extract.

Compound Name	RT(min)

Pyridine	8.429584
Propan-1-amine	9.193533
Pyridine	9.386167
1H-pyridin-2-one	9.578816
Butane-2,3-diol	9.689533
2-Hydroxypropanoic acid	10.15895
Hydroxyacetic acid	10.60405
2-Methylpropan-1-amine	11.11338
2-Aminopropanoic acid	11.32372
Butan-1-amine	11.43222
Oxalic Acid	12.03897
Pyrrolidin-2-one	12.3888
1,3-diazinane-2,4-dione	12.41537
3-Hydroxypropionic Acid	12.43308
Pyridin-4(1H)-one	12.67002
3-Hydroxybutanoic acid	12.8494
(2S)-2-(methylamino)propanoic acid	13.1793
Propanedioic acid	13.9499
Valine	14.25768
Urea	14.8489
Benzoic Acid	15.07697
2-Aminoethan-1-ol	15.52648
phosphono dihydrogen phosphate	15.5774
Leucine	15.63718
Leucine	15.63942
Propane-1,2,3-triol	15.69697
Cyclohexene-4-carboxylic acid ester	15.7014
Propane-1,2,3-triol	15.70805
(2S,3R)-butane-1,2,3-triol	16.0823
Isoleucine	16.15758
2-DEOXY(2R,3S)-Butane-1,2,3,4-tetrol	16.17087
Proline	16.24172
Aminoacetic acid	16.46095
2-oxohexanoic acid	16.58273
Butanedioic acid	16.64472
2,3-Dihydroxypropanoic acid	17.0012
Serine	17.70095
Threonine	18.2789
Pentanedioic acid	18.4959
3-Aminopropanoic acid	19.23105
3,4-dihydroxybutanoic acid	19.313
2-Hydroxybutanedioic acid	20.49762
2-Hydroxybutanedioic acid	20.50868
4-Hydroxybenzaldehyde	20.66368
(2R,3S)-Butane-1,2,3,4-tetrol	20.74563
(2R,3S)-Butane-1,2,3,4-tetrol	20.90947
2-HydroxyBenzoic Acid	20.95817
5-OxoProline	21.18403
5-OxoProline	21.20175
4-Aminobutanoic acid	21.39438
(2R,3S)-2,3,4-Trihydroxybutanoic acid	21.62025
(2R,3S)-2,3,4-Trihydroxybutanoic acid	21.97895
(2S)-2-aminopentanedioic acid	23.16802

(S)-2-Amino-3-phenylpropanoic acid	23.31417
(2R,3R,4S,5R)-5-(hydroxymethyl)oxolane-2,3,4-triol	23.9696
Asparagine	24.1069
(2R,3R,4S)-Pentane-1,2,3,4,5-pentol	24.63833
(2R,4R)-Pentane-1,2,3,4,5-pentol	24.7933
Ethenyl icosyl carbonate	24.81767
Ethenyl icosyl carbonate	24.81987
(2R,3s,4S)-Pentane-1,2,3,4,5-pentol	25.05237
(2R,4R)-Pentane-1,2,3,4,5-pentol	25.1498
3,6-ANHYDRO-(2S,3R,4S,5R,6R)-6-(hydroxymethyl)oxane-2,3,4,5-tetrol	25.65688
(2S,3R,4R,5R)-hexane-1,2,3,4,5-pentol	25.8916
(2R,3s,4S)-Pentane-1,2,3,4,5-pentol	26.03332
(3R,4S,5R)-3,4,5-Trihydroxycyclohex-1-ene-1-carboxylic acid	26.6467
(3R,4S,5R)-3,4,5-Trihydroxycyclohex-1-ene-1-carboxylic acid	26.66662
[(2R,3R,4R,5S,6R)-3-acetamido-4,5-dihydroxy-6-(hydroxymethyl)oxan-2-yl] [[(2R,3S,4R,5R)-5-(2,4-dioxopyrimidin-1-yl)-3,4-dihydroxyoxolan-2-yl]methoxy-hydroxyphosphoryl] hydrogen phosphate	26.81718
Citric Acid	26.82162
3,4-DihydroxyBenzoic Acid	26.86368
(3S,4R,5R)-1,3,4,5,6-Pentahydroxyhexan-2-one	27.47042
(3S,4R,5R)-1,3,4,5,6-Pentahydroxyhexan-2-one	27.72727
(3S,4R,5S)-1,3,4,5,6-Pentahydroxyhexan-2-one	27.72948
(3S,4R,5R)-1,3,4,5,6-Pentahydroxyhexan-2-one	27.74055
(2R)-2-[(1S)-1,2-dihydroxyethyl]-3,4-dihydroxy-2H-furan-5-one	27.7671
(3S,4R,5R)-1,3,4,5,6-Pentahydroxyhexan-2-one	27.89993
(2S,3R,4S,5R,6R)-6-(hydroxymethyl)oxane-2,3,4,5-tetrol	27.90215
(3S,4S,5R)-1,3,4,5,6-Pentahydroxy-hexan-2-one	27.90658
(2,5-Dioxo-4-imidazolidinyl) Urea	27.97077
(2R,3R,4S,5S,6R)-6-(hydroxymethyl)oxane-2,3,4,5-tetrol	28.0682
(3S,4S,5S,6R)-6-(hydroxymethyl)oxane-2,3,4,5-tetrol	28.17227
(2R,3R,4S,5S,6R)-6-(hydroxymethyl)oxane-2,3,4,5-tetrol	28.17668
(2R,3R,4S,5S,6R)-6-(hydroxymethyl)oxane-2,3,4,5-tetrol	28.5
(2R,3S,4S,5R)-Hexane-1,2,3,4,5,6-hexol	28.73695
3-[(2R)-2,4-Dihydroxy-3,3-dimethylbutanamido]propanoic acid	28.82328
(2R,3R,4R,5R)-Hexane-1,2,3,4,5,6-hexol	28.8255
(2S,3R,4R,5R)-Hexane-1,2,3,4,5,6-hexol	28.82993
(2S,3R,4R,5R)-Hexane-1,2,3,4,5,6-hexol	28.83435
(2R,3S,4R,5S)-hexane-1,2,3,4,5,6-hexol	28.89853
(2R,3R,4R,5R)-Hexane-1,2,3,4,5,6-hexol	28.90073
(2R,3R,4R,5R)-Hexane-1,2,3,4,5,6-hexol	28.90295
(1R,2S,3r,4R,5S,6s)-Cyclohexane-1,2,3,4,5,6-hexol	29.17088
4-Methylpentanoic acid	29.86622
Hexadecanoic acid	30.66782
4-Hydroxyquinoline-2-carboxylic acid	30.9756
4-Hydroxyquinoline-2-carboxylic acid	30.97782
(1R,2S,3r,4R,5S,6s)-Cyclohexane-1,2,3,4,5,6-hexol	31.33213
2-(Acetylamino)-2-deoxy-β-D-mannopyranose	31.64437
N-[2,4,5-trihydroxy-6-(hydroxymethyl)oxan-3-yl]acetamide	31.83258
2-(Acetylamino)-2-deoxy-D-galactose	31.94773
N-[2,4,5-trihydroxy-6-(hydroxymethyl)oxan-3-yl]acetamide	32.0341
(9E,12E)-Octadeca-9,12-dienoic acid	33.16567
(9Z,12Z)-Octadeca-9,12-dienoic acid	33.16788
(9Z,12Z)-Octadeca-9,12-dienoic acid	33.1701
(9Z,12Z)-Octadeca-9,12-dienoic acid	33.17232

(9Z)-Octadec-9-enoic acid	33.25868
(9Z,12Z,15Z)-Octadeca-9,12,15-trienoic acid	33.28082
Hexadecanoic acid	33.61742
Octadecanoic acid	33.63957
Octadecanoic acid	33.64178
Propane-1,2,3-triol-3-galactoside	34.3504
(2R,3R,4S,5R,6S)-2-()-6-{[(2R,3S,4R,5R,6R)-4,5,6-trihydroxy-2-(hydroxymethyl)oxan-3-yl]oxy}oxane-3,4,5-triol	37.33541
(2R,3R,4S,5S,6R)-2-{[(2S,3S,4S,5R)-3,4-Dihydroxy-2,5-bis(hydroxymethyl)oxolan-2-yl]oxy}-6-(hydroxymethyl)oxane-3,4,5-triol	38.82132

Table S6. Common and unique compounds identified in different *E. foeminea* crude extracts via Venn diagram analysis using GC–MS.

Occurrence	Total	Compound Name
ACE DCM/MeOH EA EtOH MeOH	16	Oxalic Acid
		Hexadecanoic Acid
		3-Hydroxypropionic Acid
		Pyridine
		(3S,4R,5R)-1,3,4,5,6-Pentahydroxyhexan-2-one
		Benzoic Acid
		1,3-Diazinane-2,4-dione
		(9Z,12Z)-Octadeca-9,12-Dienoic Acid
		(2R,3S)-Butane-1,2,3,4-Tetrol
		1h-Pyridin-2-one
		Hydroxyacetic Acid
		(2R,3R,4R,5R)-hexane-1,2,3,4,5,6-hexol
		2-Hydroxypropanoic Acid
		Propane-1,2,3-triol
		Butanedioic Acid
		Octadecanoic Acid
DCM/MeOH EA EtOH MeOH	1	(9Z)-Octadec-9-Enoic Acid
ACE DCM/MeOH EtOH MeOH	5	Pyrrolidin-2-one
		(3S,4R,5S)-1,3,4,5,6-Pentahydroxyhexan-2-one
		5-Oxoproline
		(2R,3S,4S)-Pentane-1,2,3,4,5-Pentol
		(3R,4S,5R)-3,4,5-Trihydroxycyclohex-1-Ene-1-Carboxylic Acid
ACE EA EtOH MeOH	2	Propan-1-Amine
		(2S,3R,4R,5R)-Hexane-1,2,3,4,5,6-hexol
DCM/MeOH EtOH MeOH	9	(2S,3R,4S,5R,6R)-6-(Hydroxymethyl)Oxane-2,3,4,5-Tetrol
		(1R,2S,3R,4R,5S,6S)-Cyclohexane-1,2,3,4,5,6-hexol
		2-Aminoethan-1-ol
		4-Aminobutanoic Acid
		Valine
		2-Hydroxybutanedioic Acid
		3,4-Dihydroxybenzoic Acid
		(2R,3R,4S,5S,6R)-6-(Hydroxymethyl)Oxane-2,3,4,5-Tetrol

		(2R,4R)-Pentane-1,2,3,4,5-Pentol
ACE DCM/MeOH MeOH	3	(9Z,12Z,15Z)-octadeca-9,12,15-trienoic acid
		(3S,4S,5S,6R)-6-(Hydroxymethyl)Oxane-2,3,4,5-Tetrol
		2-Methylpropan-1-Amine
ACE EtOH MeOH	2	(2S,3R)-Butane-1,2,3-triol
		2-Oxohexanoic Acid
ACE EA EtOH	1	Boric Acid
DCM/MeOH MeOH	5	(2R)-2-[(1S)-1,2-Dihydroxyethyl]-3,4-Dihydroxy-2H-Furan-5-one
		Aminoacetic Acid
		(2R,3R,4S)-Pentane-1,2,3,4,5-Pentol
		2,3-Dihydroxypropanoic Acid
		(2R,3R,4R,5R)-Hexane-1,2,3,4,5,6-hexol
DCM/MeOH EtOH	1	Tetradecanoic Acid
ACE DCM/MeOH	1	(2R,3R)-Butane-1,2,3,4-Tetrol
EtOH MeOH	8	Isoleucine
		Proline
		Urea
		3-Hydroxybutanoic Acid
		Butan-1-Amine
		Ethenyl Icosyl Carbonate
		(2R,3R,4S,5R)-5-(Hydroxymethyl)Oxolane-2,3,4-triol
		2-Aminopropanoic Acid
ACE MeOH	2	Pentanedioic Acid
		(2S)-2-(Methylamino)Propanoic Acid
ACE EtOH	1	(2R,3S,4R,5R)-2,3,4,5,6-Pentahydroxyhexanal
ACE EA	1	O-Methylhydroxylamine
DCM/MeOH	4	Xylose
		(3R,4R,5R)-2-(Hydroxymethyl)Oxane-2,3,4,5-Tetrol
		Acetic Acid
		(2S,3R,4S,5R,6R)-2-[(2R,3S,4S,5R)-4,5-Dihydroxy-2,5-Bis(Hydroxymethyl)Oxolan-3-yl]Oxy-6-(Hydroxymethyl)Oxane-3,4,5-triol
MeOH	34	(2R,3R,4S,5R,6S)-2-()-6-{[(2R,3S,4R,5R,6R)-4,5,6-Trihydroxy-2-(Hydroxymethyl)Oxan-3-yl]Oxy}Oxane-3,4,5-triol
		Pyridin-4(1H)-one
		(2R,3S,4R,5S)-Hexane-1,2,3,4,5,6-hexol
		3-Aminopropanoic Acid
		Propanedioic Acid
		[(2R,3R,4R,5S,6R)-3-Acetamido-4,5-Dihydroxy-6-(Hydroxymethyl)Oxan-2-yl] [[(2R,3S,4R,5R)-5-(2,4-Dioxopyrimidin-1-yl)-3,4-Dihydroxyoxolan-2-yl]Methoxy-Hydroxyphosphoryl] Hydrogen Phosphate
		2-Hydroxybenzoic Acid
		2-Deoxy(2R,3S)-Butane-1,2,3,4-Tetrol
		3-[(2R)-2,4-Dihydroxy-3,3-Dimethylbutanamido]Propanoic Acid
		N-[2,4,5-Trihydroxy-6-(Hydroxymethyl)Oxan-3-yl]Acetamide
		(3S,4S,5R)-1,3,4,5,6-Pentahydroxy-Hexan-2-one
		Propane-1,2,3-triol-3-Galactoside
		Serine
		3,6-Anhydro-(2S,3R,4S,5R,6R)-6-(Hydroxymethyl)Oxane-2,3,4,5-Tetrol

		Phosphono Dihydrogen Phosphate
		(2,5-Dioxo-4-Imidazolidinyl) Urea
		Leucine
		(2R,3S)-2,3,4-Trihydroxybutanoic Acid
		(2S,3R,4R,5R)-Hexane-1,2,3,4,5-Pentol
		Cyclohexene-4-Carboxylic Acid Ester
		(2R,3R,4S,5S,6R)-2-{[(2S,3S,4S,5R)-3,4-Dihydroxy-2,5-Bis(Hydroxymethyl)Oxolan-2-yl]Oxy}-6-(Hydroxymethyl)Oxane-3,4,5-triol
		4-Hydroxyquinoline-2-Carboxylic Acid
		4-Methylpentanoic Acid
		Threonine
		(9E,12E)-Octadeca-9,12-Dienoic Acid
		4-Hydroxybenzaldehyde
		2-(Acetylamino)-2-Deoxy-Î’-D-Mannopyranose
		Citric Acid
		(S)-2-Amino-3-Phenylpropanoic Acid
		Asparagine
		2-(Acetylamino)-2-Deoxy-D-Galactose
		(2S)-2-Aminopentanedioic Acid
		Butane-2,3-diol
		3,4-Dihydroxybutanoic Acid
EtOH	6	Nonanoic Acid
		4-Hydroxybenzoic Acid
		4-Hydroxybutanoic Acid
		(3R,4R,5R,6R)-3-Amino-6-(Hydroxymethyl)Oxane-2,4,5-triol
		2,3-Bis(Phosphonooxy)Propanoic Acid
		Dodecanoic Acid
EA	3	Methylpropanedioic Acid
		1-O-(2-Ethylhexyl) 2-O-Propan-2-yl Benzene-1,2-Dicarboxylate
		3-Hydroxybutan-2-one
ACE	2	1-Buty1-Ethylhydrazine
		1,2-Xylene

Table S7. Result from Over Representation Analysis (ORA) of all five *E. foeminea* crude extract samples.

Class group	Total	Expected	Hits	Raw p	Holm p	FDR[1]
Amino acids	277	0.13	10	1.73E-16	1.81E-13	1.81E-13
Sugar alcohols	12	0.00562	5	1.59E-14	1.67E-11	8.34E-12
Saturated Fatty Acids	38	0.0178	6	2.44E-14	2.55E-11	8.51E-12
Monosaccharides	18	0.00843	4	1.37E-10	1.43E-07	3.59E-08
Dicarboxylic acids	140	0.0656	5	8.01E-09	8.35E-06	1.68E-06
1,2-aminoalcohols	3	0.0014	2	6.51E-07	0.000677	9.72E-05
Pyridines	3	0.0014	2	6.51E-07	0.000677	9.72E-05
Hydroxybenzoic acids	6	0.00281	2	3.25E-06	0.00338	0.000425
Disaccharides	9	0.00421	2	7.79E-06	0.00809	0.000807
TCA acids	9	0.00421	2	7.79E-06	0.00809	0.000807
Unsaturated Fatty Acids	267	0.125	4	8.49E-06	0.00879	0.000807
Sugar acids	23	0.0108	2	5.45E-05	0.0564	0.00439
Organic dicarboxylic acids	23	0.0108	2	5.45E-05	0.0564	0.00439

Hydroxy Fatty Acids	274	0.128	3	0.000308	0.318	0.023
Alkali metal pyrophosphates	1	0.000468	1	0.000468	0.483	0.0288
Pyrroline carboxylic acids	1	0.000468	1	0.000468	0.483	0.0288
o-Xylenes	1	0.000468	1	0.000468	0.483	0.0288
Beta hydroxy acids	3	0.0014	1	0.0014	1	0.0734
Salicylic acids	3	0.0014	1	0.0014	1	0.0734
Hydroxybenzaldehydes	3	0.0014	1	0.0014	1	0.0734
Butenolides	4	0.00187	1	0.00187	1	0.089
Cyclitols	4	0.00187	1	0.00187	1	0.089
Acylaminosugars	6	0.00281	1	0.00281	1	0.117
Ureas	6	0.00281	1	0.00281	1	0.117
Straight chain Fatty Acids	6	0.00281	1	0.00281	1	0.117
Short-chain acids and derivatives	7	0.00328	1	0.00327	1	0.132
Quinoline carboxylic acids	8	0.00375	1	0.00374	1	0.145
Pyrimidines	9	0.00421	1	0.00421	1	0.157
Pyrimidine ribonucleoside diphosphates	10	0.00468	1	0.00467	1	0.169
Monoalkylamines	14	0.00656	1	0.00654	1	0.228
Benzoic acids	24	0.0112	1	0.0112	1	0.377
Branched Fatty Acids	38	0.0178	1	0.0176	1	0.577
Oxo Fatty Acids	43	0.0201	1	0.0199	1	0.628
Amino Fatty Acids	44	0.0206	1	0.0204	1	0.628
Hydroxy Fatty Acids	293	0.137	1	0.128	1	1
Hippuric acids	1470	0.687	2	0.151	1	1

[1] FDR: False Discovery Rate

Table S8. Result from Over Representation Analysis (ORA) of different *E. foeminea* crude extract samples.

(a) acetone extract.

**Class group**	**Total**	**Expected**	**Hits**	**Raw p**	**Holm p**	**FDR[1]**
Sugar alcohols	12	0.00187	3	7.57E-10	7.92E-07	7.92E-07
Pyridines	3	0.000468	2	7.07E-08	7.39E-05	3.70E-05
Dicarboxylic acids	140	0.0219	3	1.52E-06	0.00159	0.00053
Monosaccharides	18	0.00281	2	3.60E-06	0.00376	0.000942
Saturated Fatty Acids	38	0.00593	2	1.65E-05	0.0172	0.00346
Pyrroline carboxylic acids	1	0.000156	1	0.000156	0.162	0.0233
o-Xylenes	1	0.000156	1	0.000156	0.162	0.0233
Cyclitols	4	0.000624	1	0.000624	0.649	0.0816
Unsaturated Fatty Acids	267	0.0417	2	0.000816	0.847	0.0898
Hydroxy Fatty Acids	274	0.0428	2	0.000859	0.891	0.0898
TCA acids	9	0.0014	1	0.0014	1	0.122
Pyrimidines	9	0.0014	1	0.0014	1	0.122
Benzoic acids	24	0.00375	1	0.00374	1	0.301
Oxo Fatty Acids	43	0.00671	1	0.00669	1	0.5
Hippuric acids	1470	0.229	1	0.205	1	1

(b) dichloromethane/methanol extract.

**Class group**	**Total**	**Expected**	**Hits**	**Raw p**	**Holm p**	**FDR[1]**
Saturated Fatty Acids	38	0.00842	4	1.54E-10	1.61E-07	1.61E-07
Sugar alcohols	12	0.00266	3	2.23E-09	2.33E-06	1.17E-06
Pyridines	3	0.000665	2	1.44E-07	0.00015	5.02E-05

Monosaccharides	18	0.00399	2	7.32E-06	0.00764	0.00192
Unsaturated Fatty Acids	267	0.0592	3	3.06E-05	0.0318	0.00639
Pyrroline carboxylic acids	1	0.000222	1	0.000222	0.231	0.0386
Dicarboxylic acids	140	0.031	2	0.000458	0.476	0.0684
1,2-aminoalcohols	3	0.000665	1	0.000665	0.69	0.0869
Butenolides	4	0.000886	1	0.000886	0.92	0.0927
Cyclitols	4	0.000886	1	0.000886	0.92	0.0927
Hydroxybenzoic acids	6	0.00133	1	0.00133	1	0.116
Straight chain Fatty Acids	6	0.00133	1	0.00133	1	0.116
Hydroxy Fatty Acids	274	0.0607	2	0.00173	1	0.123
Amino acids	277	0.0614	2	0.00176	1	0.123
Disaccharides	9	0.00199	1	0.00199	1	0.123
TCA acids	9	0.00199	1	0.00199	1	0.123
Pyrimidines	9	0.00199	1	0.00199	1	0.123
Sugar acids	23	0.0051	1	0.00508	1	0.292
Benzoic acids	24	0.00532	1	0.0053	1	0.292
Amino Fatty Acids	44	0.00975	1	0.0097	1	0.507
Hippuric acids	1470	0.325	2	0.0421	1	1

(c) ethanol extract.

Class group	Total	Expected	Hits	Raw p	Holm p	FDR[1]
Saturated Fatty Acids	38	0.00976	5	4.55E-13	4.76E-10	4.76E-10
Sugar alcohols	12	0.00308	3	3.50E-09	3.66E-06	1.83E-06
1,2-aminoalcohols	3	0.00077	2	1.94E-07	0.000202	5.07E-05
Pyridines	3	0.00077	2	1.94E-07	0.000202	5.07E-05
Amino acids	277	0.0711	4	8.79E-07	0.000916	0.000169
Hydroxybenzoic acids	6	0.00154	2	9.69E-07	0.00101	0.000169
Monosaccharides	18	0.00462	2	9.87E-06	0.0103	0.00147
Hydroxy Fatty Acids	274	0.0704	3	5.15E-05	0.0535	0.00673
Pyrroline carboxylic acids	1	0.000257	1	0.000257	0.267	0.0298
Dicarboxylic acids	140	0.036	2	0.000615	0.638	0.0643
Beta hydroxy acids	3	0.00077	1	0.00077	0.798	0.0732
Cyclitols	4	0.00103	1	0.00103	1	0.0826
1,2-diols	4	0.00103	1	0.00103	1	0.0826
Ureas	6	0.00154	1	0.00154	1	0.115
Short-chain acids and derivatives	7	0.0018	1	0.0018	1	0.125
Unsaturated Fatty Acids	267	0.0686	2	0.0022	1	0.134
TCA acids	9	0.00231	1	0.00231	1	0.134
Pyrimidines	9	0.00231	1	0.00231	1	0.134
Monoalkylamines	14	0.0036	1	0.00359	1	0.198
Benzoic acids	24	0.00616	1	0.00615	1	0.321
Hippuric acids	1470	0.377	3	0.00645	1	0.321
Oxo Fatty Acids	43	0.011	1	0.011	1	0.511
Amino Fatty Acids	44	0.0113	1	0.0112	1	0.511

(d) ethyl acetate extract.

Class group	Total	Expected	Hits	Raw p	Holm p	FDR[1]
Sugar alcohols	12	0.00133	3	2.59E-10	2.71E-07	2.71E-07
Pyridines	3	0.000332	2	3.51E-08	3.67E-05	1.84E-05

Dicarboxylic acids	140	0.0155	3	5.23E-07	0.000546	0.000182
Saturated Fatty Acids	38	0.00421	2	8.22E-06	0.00857	0.00215
Unsaturated Fatty Acids	267	0.0296	2	0.000409	0.426	0.075
Hydroxy Fatty Acids	274	0.0304	2	0.00043	0.448	0.075
TCA acids	9	0.000997	1	0.000997	1	0.13
Pyrimidines	9	0.000997	1	0.000997	1	0.13
Organic dicarboxylic acids	23	0.00255	1	0.00254	1	0.278
Benzoic acids	24	0.00266	1	0.00266	1	0.278

(e) methanol extract.

**Class group**	**Total**	**Expected**	**Hits**	**Raw p**	**Holm p**	**FDR[1]**
Amino acids	277	0.107	10	2.39E-17	2.50E-14	2.50E-14
Sugar alcohols	12	0.00465	5	6.07E-15	6.34E-12	3.17E-12
Dicarboxylic acids	140	0.0543	4	3.08E-07	0.000321	0.000107
Pyridines	3	0.00116	2	4.45E-07	0.000464	0.000116
Unsaturated Fatty Acids	267	0.104	4	4.00E-06	0.00417	0.000837
TCA acids	9	0.00349	2	5.33E-06	0.00555	0.00093
Monosaccharides	18	0.00698	2	2.26E-05	0.0235	0.00338
Sugar acids	23	0.00892	2	3.73E-05	0.0388	0.00488
Saturated Fatty Acids	38	0.0147	2	0.000103	0.107	0.012
Alkali metal pyrophosphates	1	0.000388	1	0.000388	0.402	0.0369
Pyrroline carboxylic acids	1	0.000388	1	0.000388	0.402	0.0369
1,2-aminoalcohols	3	0.00116	1	0.00116	1	0.0811
Beta hydroxy acids	3	0.00116	1	0.00116	1	0.0811
Salicylic acids	3	0.00116	1	0.00116	1	0.0811
Hydroxybenzaldehydes	3	0.00116	1	0.00116	1	0.0811
Butenolides	4	0.00155	1	0.00155	1	0.0954
Cyclitols	4	0.00155	1	0.00155	1	0.0954
Hydroxybenzoic acids	6	0.00233	1	0.00232	1	0.122
Acylaminosugars	6	0.00233	1	0.00232	1	0.122
Ureas	6	0.00233	1	0.00232	1	0.122
Quinoline carboxylic acids	8	0.0031	1	0.0031	1	0.154
Disaccharides	9	0.00349	1	0.00348	1	0.158
Pyrimidines	9	0.00349	1	0.00348	1	0.158
Pyrimidine ribonucleoside diphosphates	10	0.00388	1	0.00387	1	0.169
Hydroxy Fatty Acids	274	0.106	2	0.00518	1	0.217
Monoalkylamines	14	0.00543	1	0.00541	1	0.218
Organic dicarboxylic acids	23	0.00892	1	0.00888	1	0.344
Benzoic acids	24	0.00931	1	0.00926	1	0.346
Branched Fatty Acids	38	0.0147	1	0.0146	1	0.528
Oxo Fatty Acids	43	0.0167	1	0.0165	1	0.571
Amino Fatty Acids	44	0.0171	1	0.0169	1	0.571
Hydroxy Fatty Acids	293	0.114	1	0.107	1	1
Hippuric acids	1470	0.569	2	0.111	1	1

[1] FDR: False Discovery Rate
